# Combining Chalcones with Donepezil to Inhibit Both Cholinesterases and Aβ Fibril Assembly

**DOI:** 10.3390/molecules25010077

**Published:** 2019-12-24

**Authors:** Nishad Thamban Chandrika, Marina Y. Fosso, Oleg V. Tsodikov, Harry LeVine, Sylvie Garneau-Tsodikova

**Affiliations:** 1 Department of Pharmaceutical Sciences, College of Pharmacy, University of Kentucky, Lexington, KY 40536-0596, USA; nishad.tc@uky.edu (N.T.C.); fossoyatchang@gmail.com (M.Y.F.); oleg.tsodikov@uky.edu (O.V.T.); 2 Center on Aging, School of Medicine, University of Kentucky, Lexington, KY 40536-0230, USA; harry.levine@uky.edu; 3 Department of Molecular and Cellular Biochemistry, School of Medicine, University of Kentucky, Lexington, KY 40536-0230, USA

**Keywords:** Alzheimer’s disease, acetylcholinesterase, butyrylcholinesterase, ^3^H-PIB binding, Aβ assembly, Aβ dissociation

## Abstract

The fact that the number of people with Alzheimer’s disease is increasing, combined with the limited availability of drugs for its treatment, emphasize the need for the development of novel effective therapeutics for treating this brain disorder. Herein, we focus on generating 12 chalcone-donepezil hybrids, with the goal of simultaneously targeting amyloid-β (Aβ) peptides as well as cholinesterases (i.e., acetylcholinesterase (AChE) and butyrylcholinesterase (BChE)). We present the design, synthesis, and biochemical evaluation of these two series of novel 1,3-chalcone-donepezil (**15a**–**15f**) or 1,4-chalcone-donepezil (**16a**–**16f**) hybrids. We evaluate the relationship between their structures and their ability to inhibit AChE/BChE activity as well as their ability to bind Aβ peptides. We show that several of these novel chalcone-donepezil hybrids can successfully inhibit AChE/BChE as well as the assembly of *N*-biotinylated Aβ_(1–42)_ oligomers. We also demonstrate that the Aβ binding site of these hybrids differs from that of Pittsburgh Compound B (PIB).

## 1. Introduction

Alzheimer’s disease (AD) is a progressive neurodegenerative disorder that causes brain cells to waste away, resulting in deterioration of memory, cognitive, and executive functions [1,2,3,4,5,6]. Even though the estimates vary, there are as many as 5.5 million Americans age 60 and older with AD [7]. There are no medications available that cure AD or stop the disease progression in the brain. In the advanced stages, complications from the disease can cause dehydration, malnutrition, or infections that ultimately may lead to death [8,9,10,11,12,13]. Since increasing age is the most common risk factor for the development of AD, barring the implementation of an effective treatment or prevention of AD, the number of people with AD will increase significantly if the current population lifespan trends continue. The few medications that are approved for AD (donepezil, tacrine, rivastigmine, galantamine (acetylcholinesterase inhibitors (AChEIs)) and memantine (*N*-methyl-d-aspartate (NMDA) receptor antagonist) provide only limited symptomatic relief rather than affecting disease progression or providing a cure [14,15,16,17]. Since the number of AD-related drugs used in AD patients is limited, there is an urgent need for novel therapeutic candidates to treat this brain disorder.

Despite extensive research efforts, the causative factors of AD remain unclear [18]. However, various characteristics, such as low levels of acetylcholine (ACh), accumulation of amyloid-β (Aβ) deposits, tau (τ) protein aggregation, oxidative stress, inflammation, metal ion imbalance, and breakdown of homeostatic systems, play crucial roles in AD progression [19,20,21,22,23,24,25]. Currently, most of the primary therapeutic options for treating AD are based on AChEIs [26,27,28,29,30,31]. Low levels of acetylcholine (ACh) can lead to cognitive and memory deficits. The two types of cholinesterases present in the central nervous system, acetylcholinesterase (AChE) and butyrylcholinesterase (BChE), are capable of hydrolyzing ACh [32,33,34,35]. In healthy brains, BChE plays a secondary role to AChE, but as AD progresses the activity and expression of BChE in the brain increases [36]. Although developing inhibitors against these enzymes alone will not lead to a cure, combining their effect with another hallmark of AD progression, such as Aβ peptide accumulation, could potentially be beneficial. The accumulation of Aβ peptides in the cerebral cortex of the brain has been suggested as a part of AD pathogenesis [37,38]. Thus, additionally preventing the aggregation of Aβ and/or disrupting the existing Aβ plaques are potential therapeutic approaches for treating AD [22,39,40,41].

There are currently reports in the literature investigating new scaffolds with two or more pharmacophores connected to each other through a linker aimed at interacting with more than one biological target [42,43,44,45,46,47,48,49,50,51]. Previously, our group as well as others studied donepezil, the current drug of choice for treating AD with its AChE and BChE inhibiting capacity [52,53,54,55,56,57]. In addition, we explored the use of chalcone derivatives as AChE inhibitors as well as Aβ peptide and metal–Aβ complex-targeting compounds [58,59]. Here, we hypothesized that coupling various chalcones and donepezil with linkers of different lengths could result in chalcone-donepezil hybrids capable of inhibiting cholinesterases as well as targeting Aβ peptides. We report the design and synthesis of 12 such hybrids and evaluate their structure–activity relationship (SAR) for their ability to inhibit AChE and BChE as well as to prevent and/or disrupt Aβ peptide oligomerization. We also investigate the binding site of these hybrids by testing their ability to compete with Pittsburgh Compound B (PIB) for binding to natural and synthetic fibrils of Aβ.

## 2. Results and Discussion

### 2.1. Design and Synthesis of 1,3-Chalcone-Donepezil (***15a***–***15f***) and 1,4-Chalcone-Donepezil (***16a***–***16f***) Hybrids

**Scheme 1:**
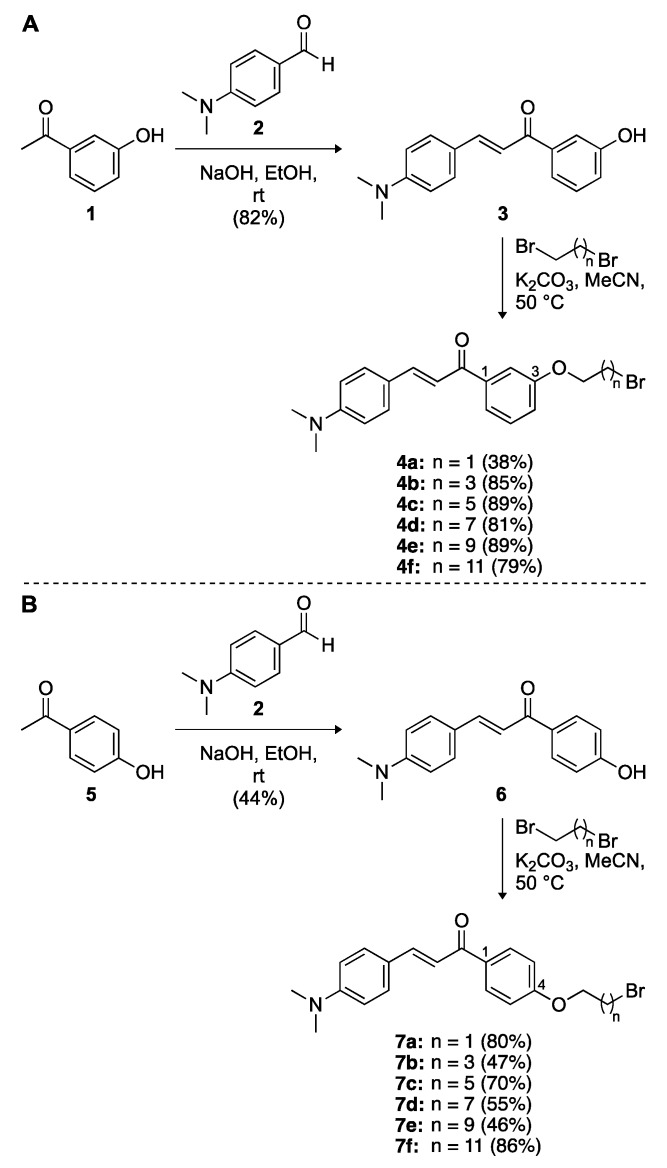
Synthetic schemes for the preparation of (**A**) 1,3-chalcones **4a**–**4f** and (**B**) 1,4-chalcones **7a**–**7f**.

In order to develop multitarget-directed scaffolds for AD, we designed a series of 1,3- and 1,4-chalcone-donepezil hybrids in a way that the chalcone and donepezil scaffolds were attached to each other through linkers consisting of 2, 4, 6, 8, 10, and 12 carbons. Overall, an electrophilic center was introduced on the chalcone scaffold in the form of a brominated linker, which was then reacted with a donepezil nucleophile to generate the target molecules (Scheme 1). We first performed a two-step synthesis to add linkers of varied lengths to a series of 1,3- and 1,4-chalcones (Scheme 1). Briefly, the condensation of 4-(dimethylamino)benzaldehyde (**2**) with 3-hydroxyacetophenone (**1**) or 4-hydroxyacetophenone (**5**) in the presence of NaOH resulted in 1,3-chalcone **3** and 1,4-chalcone **6** in 82% and 44% yields, respectively. The free hydroxyl group of compounds **3** and **6** was reacted with *n*-alkyl dibromides with 2, 4, 6, 8, 10, and 12 carbons to generate compounds **4a**–**4f** (38–89%) and **7a**–**7f** (46–86%), respectively.

**Scheme 2:**
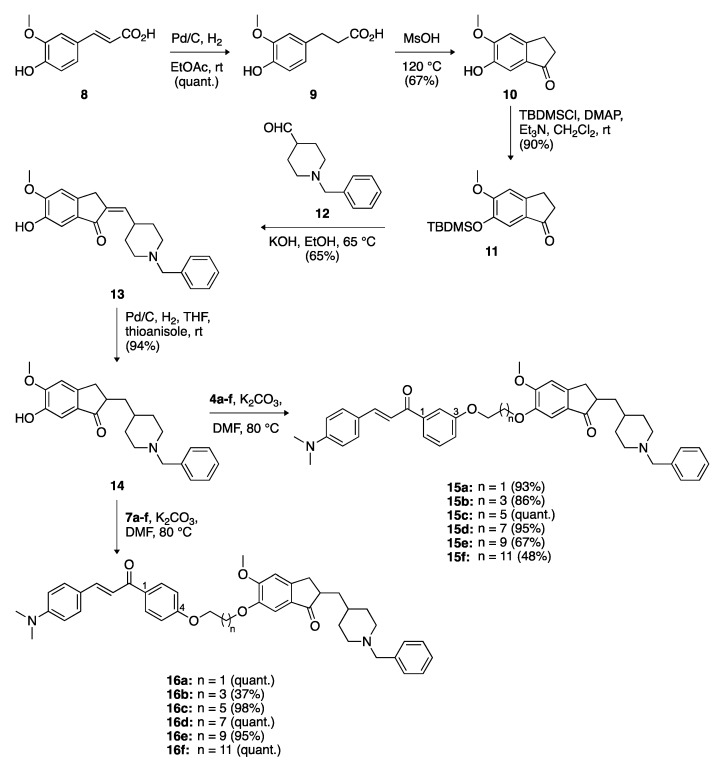
Synthetic scheme for the preparation of 1,3-chalcone-donepezil hybrids **15a**–**15f** and 1,4-chalcone-donepezil hybrids **16a**–**16f**.

We next synthesized the donepezil nucleophile **14** in five steps (Scheme 2) consisting of the hydrogenation of ferulic acid **8** in the presence of Pd/C, followed by cyclization in the presence of methanesulfonic acid to result in formation of compound **10** in a 67% yield. Since the condensation of compound **10** with aldehyde **12** was found to be problematic, we had to protect the free hydroxyl group of compound **10** with a *tert*-butyldimethylsilyl (TBDMS) group to afford the protected compound **11** in 90% yield. Compound **11** was then successfully condensed with aldehyde **12** in the presence of KOH to give compound **13** in 65% yield. The controlled deactivation of the palladium catalyst with thioanisole was essential for selective reduction of the double bond in the presence of ketone and benzyl groups in compound **13** to provide the 6-*O*-desmethyl donepezil adduct **14** in 94% yield. With the donepezil nucleophile **14** in hand, we finally reacted its free hydroxyl group with two sets of electrophilic alkylated 1,3- and 1,4-chalcones, **4a**–**4f** and **7a**–**7f**, to afford the desired 1,3- and 1,4-chalcone-donepezil hybrids **15a**–**15f** and **16a**–**16f** in 48% to 100% and 37% to 100% yields, respectively.

**Scheme 3:**
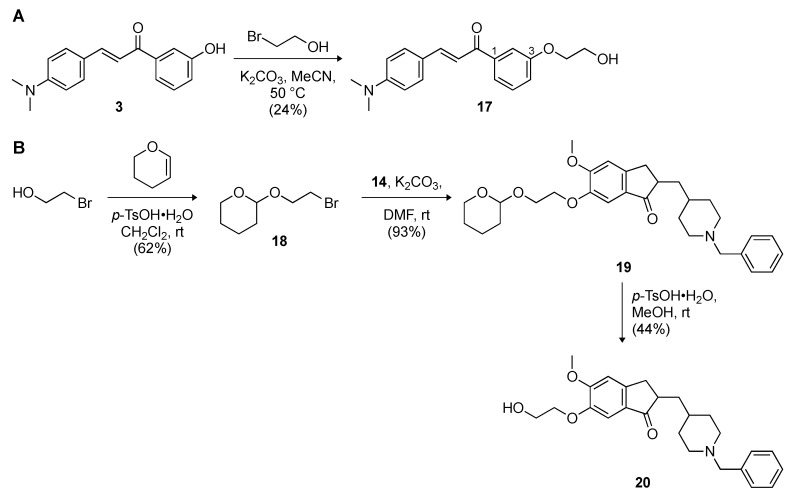
Synthetic schemes for the preparation of (**A**) 1,3-chalcone **17** used for combination studies with donepezil and comparison with 1,3-chalcone-donepezil hybrid **15a**, and (**B**) donepezil derivative **20** used for combination studies with 1,3-chalcone **3** and comparison with 1,3-chalcone-donepezil hybrid **15a**.

With the goal of investigating the importance of the covalent linkage between the chalcones and donepezil, we synthesized chalcone and donepezil fragments with a C_2_ linker (Scheme 3). The parent 1,3-chalcone **3** was reacted with bromoethanol in the presence of K_2_CO_3_ to give compound **17** in 24% yield. In the case of the donepezil fragment, the hydroxyl group of bromoethanol was protected with dihydropyran to give compound **18** in 62% yield. This molecule was subjected to a nucleophilic substitution reaction with the donepezil nucleophile **14** to yield **19**, which was in turn subjected to the removal of the tetrahydropyran group to give compound **20** in 44% yield.

### 2.2. Cholinesterase Inhibitory Activity

To evaluate the potential cholinesterase inhibitory activity of the 12 newly synthesized 1,3- and 1,4-chalcone-donepezil hybrids, **15a**–**15f** and **16a**–**16f**, we determined their half maximal inhibitory concentration (IC_50_) values against *Ee*AChE (AChE from *Electrophorus electricus*) (Table 1 and Figure S66) and *Ef*BChE (BChE from *Equus ferus*) (Table 1 and Figure S67) using the Ellman’s method [60].

#### 2.2.1. AChE Inhibition

In general, from comparing 1,3- and 1,4-chalcone-donepezil hybrids (**15** versus **16**) pairwise (for example, **15a** with **16a**, etc.) we observed that in all cases, with the exception of **15f** versus **16f**, the 1,4-chalcone-donepezil hybrids were more effective at inhibiting *Ee*AChE. In general, most of them displayed slightly higher IC_50_ values (1- to 15-fold) when compared to donepezil, with the exception of compounds **16a** (0.07 ± 0.01 μM, 2 carbon linker) and **16b** (0.14 ± 0.02 μM, 4 carbon linker), which showed equal or better inhibition than donepezil. Out of all the compounds, in the case of the 1,3-chalcone-donepezil series, the two best compounds were **15a** (1.02 ± 0.33 μM, 2 carbon linker) and **15b** (0.65 ± 0.14 μM, 4 carbon linker). Similarly, in the case of the 1,4-chalcone-donepezil series, **16a** (0.07 ± 0.01 μM, 2 carbon linker) and **16b** (0.14 ± 0.02 μM, 4 carbon linker) were the best at inhibiting *Ee*AChE. It appears that increasing the linker length from 2 to 12 carbons gradually increased the IC_50_ values, as exemplified by the values shown by compounds **15a**–**15f** (1.02 ± 0.33 μM, 0.65 ± 0.14 μM, 1.22 ± 0.18 μM, 1.22 ± 0.13 μM, 1.82 ± 0.45 μM, 1.67 ± 0.80 μM) and **16a**–**16f** (0.07 ± 0.01 μM, 0.14 ± 0.02 μM, ≈0.5 μM, 0.52 ± 0.06 μM, 1.38 ± 0.18 μM, >100 μM). Interestingly, with an IC_50_ >100 μM, **16f** (12 carbon linker) was unable to inhibit *Ee*AChE, whereas **15f** (12 carbon linker), with an IC_50_ value of 1.67 ± 0.80 μM, was much more potent. Since we observed better inhibition of *Hs*AChE (from *Homo sapiens*) than *Ee*AChE for donepezil derivatives in one of our previous studies [52], we postulated that the values observed here for *Ee*AChE should translate to *Hs*AChE. We therefore decided to only utilize *Ee*AChE in the current study as it is much more easily accessible.

#### 2.2.2. BChE Inhibition

When we tested the hybrids against *Ef*BChE, we observed a similar trend to that seen against *Ee*AChE. In general, 1,4-chalcone-donepezil hybrids (**16**) were better than 1,3-chalcone-donepezil hybrids (**15**) at inhibiting *Ef*BChE, with the exception of compounds with 2 carbon (**15a**, 0.020 ± 0.002 μM) and 4 carbon linkers (**15b**, 0.03 ± 0.01 μM). In the case of 1,3-chalcone-donepezil hybrids, the compounds with 2 and 4 carbon linkers (**15a** and **15b**) were much better than donepezil (2.0 ± 0.10 μM) at inhibiting *Ef*BChE. In the 1,4-chalcone-donepezil series, the hybrids with 2 (**16a**, ≈0.1 μM), 4 (**16b**, ≈0.5 μM), 6 (**16c**, ≈0.6 μM), and 8 (**16d**, ≈1.0 μM) carbon linkers were better than donepezil (2.0 ± 0.10 μM) at inhibiting *Ef*BChE. Against this enzyme, both compounds **15f** (>100 μM, 12 carbon linker) and **16f** (>100 μM, 12 carbon linker) were found to be inactive. The data gathered from the *Ee*AChE and *Ef*BChE inhibition studies point towards the benefit of having smaller linkers between the chalcones and donepezil.

In order to understand the utility of the hybrids, based on their IC_50_ values against *Ee*AChE and *Ef*BChE, we determined their selectivity index (SI) (Table 1). For all but two hybrids, **15a** and **15b**, *Ee*AChE was inhibited equally to >60-fold better than *Ef*BChE. In the case of **15a** and **15b**, the inhibition of *Ef*BChE was 51-fold and 22-fold better than that of *Ee*AChE, respectively.

### 2.3. ^3^H-PIB Binding Studies

**Figure 1:**
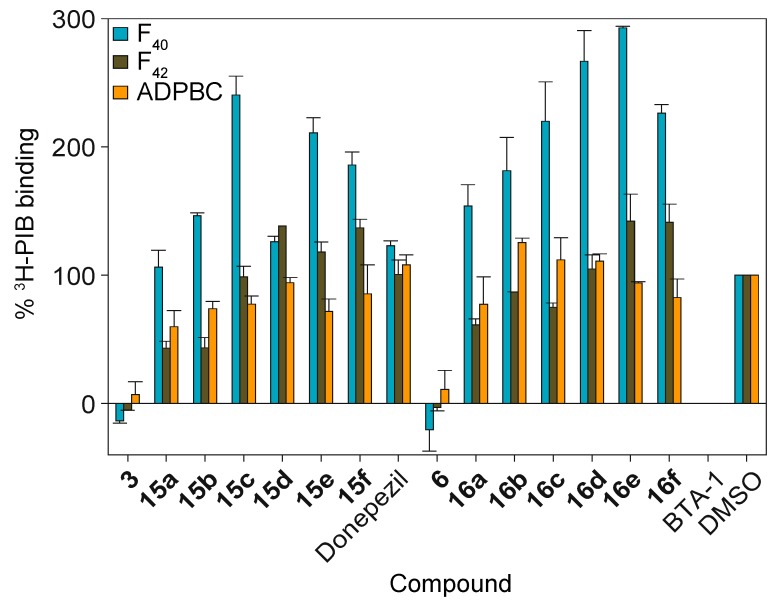
The measurement of competition of chalcone-donepezil hybrids **15a**–**15f** and **16a**–**16f** against ^3^H-PIB binding to Aβ fibrils and ADPBC at 10 μM. BTA-1 (2-(4′-methylaminophenyl)benzothiazole) was used as a positive control. F_40_ = Aβ_(1–40)_ fibrils; F_42_ = Aβ_(1–42)_ fibrils; ADPBC = PIB binding site located in the AD brain. Due to solubility-micellarization concerns that interferes with ^3^H-PIB binding measurements, compounds were not tested at concentrations greater than 10 μM. These experiments were performed in duplicate (*n* = 1 independent experiment). The error bars represent standard deviations (SDEV).

Previously, we demonstrated that various chalcones can displace ^3^H-PIB from the Alzheimer’s disease PIB binding complex (ADPBC) purified from AD brains, as well as from more widely available model fibrils assembled from synthetic Aβ_(1–40)_ and Aβ_(1–42)_ peptides (indicated herein as F_40_ and F_42_, respectively) [59]. When studied for their ability to displace ^3^H-PIB from F_40_ (Figure 1 turquoise bars), F_42_ (brown bars), and ADPBC (orange bars), we found that 1,3- and 1,4-chalcone-donepezil hybrids did not displace ^3^H-PIB as efficiently as chalcones **3** and **6**. Even though there seemed to be no direct correlation between the linker length of the hybrids and their activity, hybrids with shorter linkers appeared to better displace ^3^H-PIB from F_40_ and F_42_ (i.e., compounds **15a** and **15b** displayed better binding to F_40_, F_42_, and ADPBC than **15c**, **15d**, **15e**, and **15f**; compound **16a** displayed better affinity for F_40_ and F_42_ than **16b**, **16c**, **16d**, **16e**, and **16f**). Interestingly, hybrids **16e** and **16f** displayed ADPBC affinity comparable to that of **16a**. These results indicated that the chalcone-donepezil hybrids probably bind to the fibrils at a different location than ^3^H-PIB does in the AD brain. Interestingly, during the course of these experiments, we observed that hybrids **15a**–**15f** and **16a**–**16f** enhanced binding to F_40_. We speculate that when the hybrids bind to F_40_, which is known to be more malleable than F_42_, the conformation of the F_40_ peptide/fibril changes in a way that allow for additional PIB binding. Investigation of this phenomenon is beyond the scope of the current study.

**Figure 2:**
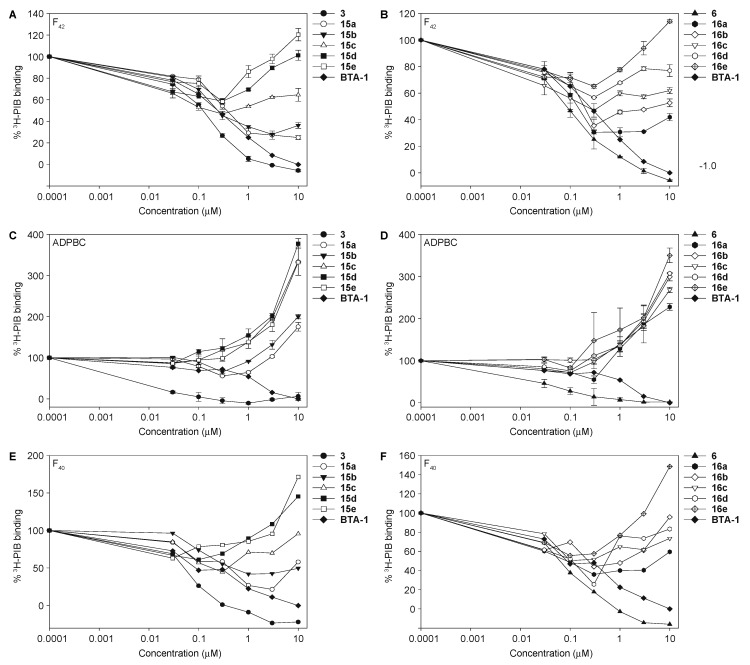
The titration of chalcone-donepezil hybrids **15a**–**15e** and **16a**–**16e** against ^3^H-PIB binding to F_42_ (Aβ_(1–42)_ fibrils) (**A**,**B**), ADPBC (PIB binding site located in the AD brain) (**C**,**D**), and F_40_ (Aβ_(1–40)_ fibrils) (**E**,**F**). BTA-1 (2-(4′-methylaminophenyl)benzothiazole) was used as a positive control. The experiments with F_42_ and ADPBC were performed in duplicate (*n* = 1 independent experiment). The error bars represent standard deviations (SDEV).

To confirm the results obtained from the ^3^H-PIB binding competition studies, we titrated the 1,3-and 1,4-chalcone-donepezil hybrids against ^3^H-PIB bound to F_40_, F_42_, and ADPBC fibrils. The experiments were performed in duplicate for F_42_ and ADPBC and as a single experiment for F_40_. Even though the single independent experiment may not be sufficient to determine the exact value of inhibition, we were able to observe a clear trend, which is sufficient at this stage of investigation. The data obtained by titration assays (Figure 2) were completely in accord with those displayed in Figure 1 for both the 1,3- and 1,4-chalcone-donepezil hybrids.

### 2.4. The Effect of Chalcone-Donepezil Hybrids on Aβ Assembly and Dissociation

The progression of AD neurodegenerative disease is characterized by the accumulation of Aβ plaques followed by the loss of neurons and tau pathology accumulation leading to brain atrophy and dementia. Even though Aβ fibrils are responsible for plaque formation, recent research efforts have pointed out the role of soluble Aβ oligomers in synaptic neurotoxicity [61]. Genetic evidence and autopsy observations of human cases of AD suggest that the buildup and pathogenicity of Aβ is fundamental to the progression of the disease. Since AD progression is tightly connected to Aβ aggregation, it is imperative to evaluate the effect of the 1,3- and 1,4-chalcone-donepezil hybrids **15a**–**15f** and **16a**–**16f** on Aβ oligomer assembly and dissociation. Among Aβ peptide assemblies, such as Aβ_(1–40)_ (F_40_) and Aβ_(1–42)_ (F_42_) fibrils, the Aβ_(1–42)_ peptide is the most abundant and neuro- and synaptotoxic [62,63,64,65].

**Figure 3:**
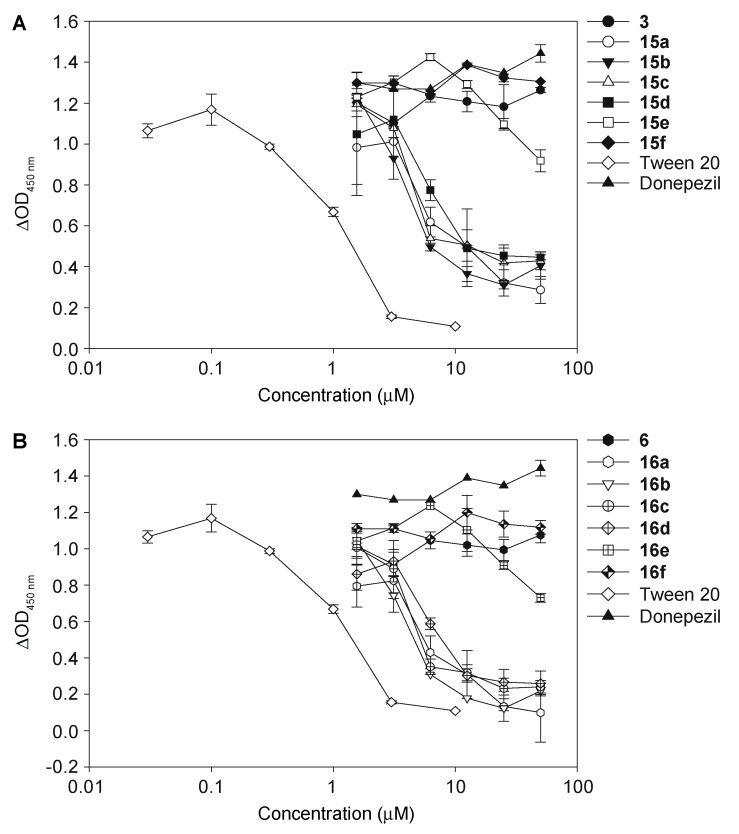
The measurement of biotinyl-Aβ_(1–42)_ (bioAβ_42_) oligomer assembly inhibition of compounds **3** and **15a**–**15f** (**A**), and compounds **6** and **16a**–**16f** (**B**). The experiments were performed over a concentration range of 1.56 to 50 μM. Donepezil and Tween 20 (assembly inhibition) were used as controls. For Tween 20, the concentration range used was 0.03–10 μM. The absorbance of the enzymatic readout of oligomer content was determined at 450 nm with a BioTek HT Synergy plate reader. These experiments were performed in duplicate (*n* = 1 independent experiment). The error bars represent standard deviations (SDEV).

We first examined the effect of the synthesized 1,3- and 1,4-chalcone-donepezil hybrids **15a**–**15f** and **16a**–**16f** on the assembly of *N*-biotinyl-Aβ_(1–42)_ (bioAβ_42_) monomers into oligomers. We quantified the assembly of bioAβ_42_ into soluble oligomers by using an ELISA assay in which the soluble oligomers were captured on NeutrAvidin^TM^-coated ELISA plates and detected with streptavidin-horseradish peroxidase and colorimetric readout (Figure 3). Among the hybrids, those with shorter linkers (2–8 carbons), **15a**–**15d** and **16a**–**16d**, demonstrated better inhibition of bioAβ_42_ oligomer assembly than the parent chalcones **3** and **6** as well as the donepezil from which they were made, respectively. We found that hybrids with longer linkers (10 and 12 carbons), such as **15e**, **15f**, **16e**, and **16f**, did not prevent bioAβ_42_ oligomerization. From these data, we could infer that attaching donepezil to chalcones is highly beneficial as it increases their ability to prevent assembly of bioAβ_42_ oligomers.

**Figure 4:**
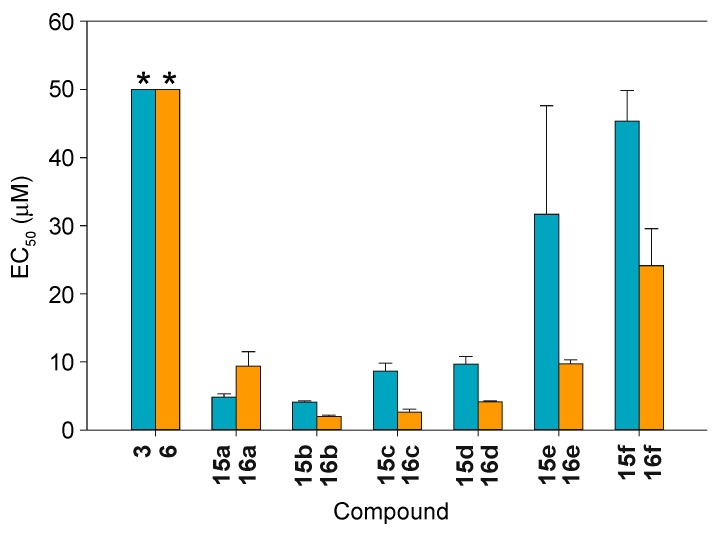
The measurement of half maximal effective concentration (EC_50_) values of chalcones **3** (turquoise bar) and **6** (orange bar) as well as 1,3- and 1,4-chalcone-donepzil hybrids **15a**–**15f** (turquoise bars) and **16a**–**16f** (orange bars) against biotinyl-Aβ_(1–42)_ (bioAβ_42_) oligomer assembly inhibition. These experiments were performed in duplicate (*n* = 3 independent experiments). The error bars represent standard deviations (SDEV). The * on top of the bars for compounds **3** and **6** indicate that these values are in fact >50 μM.

Next, we determined the half maximal effective concentration (EC_50_) values for all of the hybrids synthesized, **15a**–**15f** and **16a**–**16f**, against bioAβ_42_ oligomer assembly (Figure 4 and Table S1). In general, the 1,4-chalcone-donepezil hybrids (**16b**–**16f**) displayed lower EC_50_ values than their corresponding 1,3-chalcone-donepezil hybrid counterparts (**15b**–**15f**), with the exception of **16a**, which had a slightly higher EC_50_ value than **15a**. All compounds tested exhibited better EC_50_ values than chalcone alone. Compounds **15a** (4.83 ± 0.47 μM), **15b** (4.10 ± 0.17 μM), **15c** (8.67 ± 0.15 μM), **15d** (9.67 ± 1.15 μM), **16a** (9.37 ± 2.10 μM), **16b** (2.00 ± 0.20 μM), **16c** (2.60 ± 0.46 μM), **16d** (4.13 ± 0.15 μM), and **16e** (9.73 ± 0.55 μM) displayed EC_50_ values <12 μM and a high affinity for bioAβ_42_, whereas **15e**, **15f**, and **16f** had EC_50_ values >12 μM (up to 45.33 ± 4.51 μM). These data emphasized the importance of having a shorter linker between the chalcone and donepezil scaffolds. Overall, in the case of both 1,3- and 1,4-chalcone-donepzil hybrids, the less lipophilic compounds displayed better inhibition of bioAβ_42_ oligomerization.

**Figure 5:**
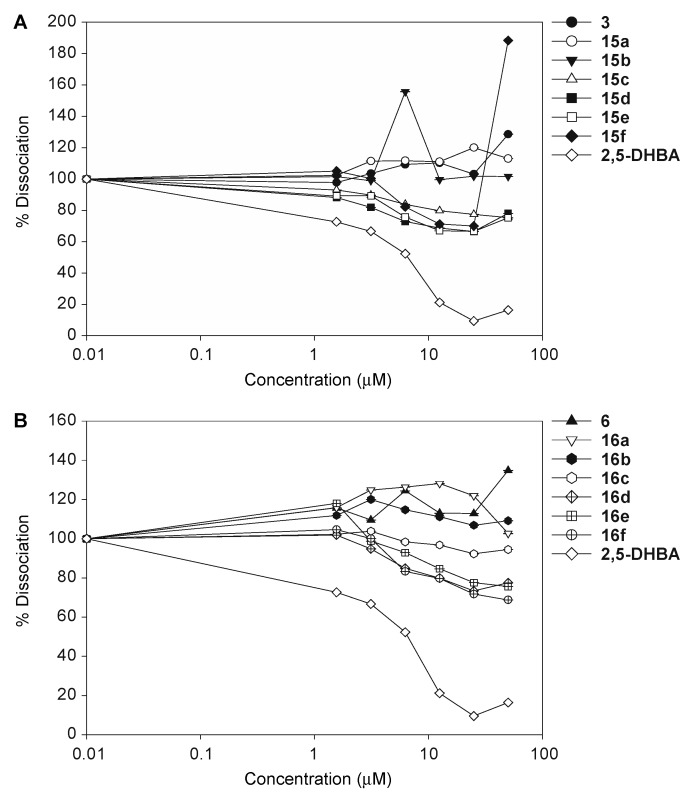
The measurement of biotinyl-Aβ_(1–42)_ (bioAβ_42_) oligomer dissociation of compounds **3** and **15a**–**15f** (**A**), and compounds **6** and **16a**–**16f** (**B**). The experiments were performed over a concentration range of 0.01 to 50 μM. 2,5-DHBA (2,5-dihydroxybenzoic acid) was used as a dissociator positive control. These experiments were performed in duplicate (*n* = 1 independent experiment). The error bars represent standard deviations (SDEV).

In addition to assess the effect of the synthesized 1,3- and 1,4-chalcone-donepezil hybrids **15a**–**15f** and **16a**–**16f** on the assembly of bioAβ_42_ monomers into oligomers, we also investigated their effect on the dissociation of preformed bioAβ_42_ oligomers. While some of these hybrids were able to prevent assembly, none of them could dissociate preformed bioAβ_42_ oligomers (Figure 5).

**Figure 6:**
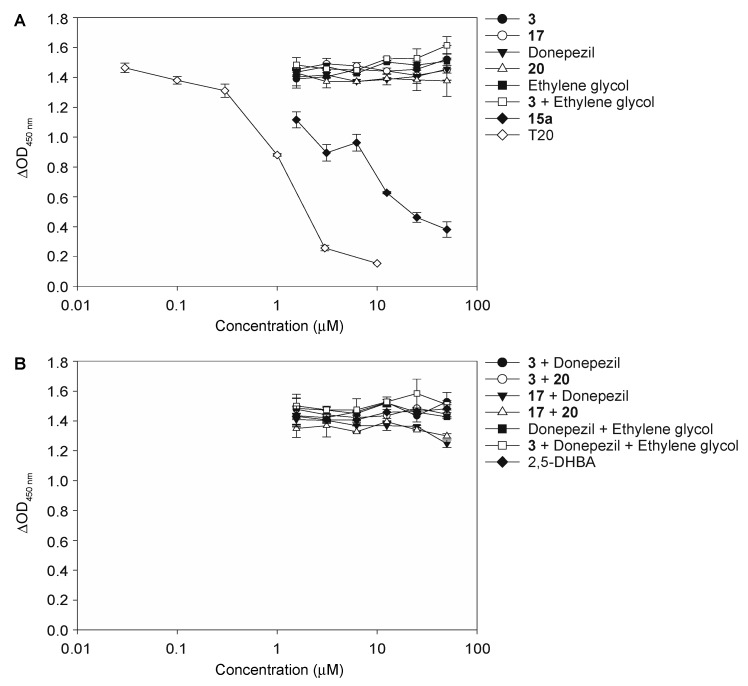
The measurement of biotinyl-Aβ_(1–42)_ (bioAβ_42_) oligomer assembly inhibition of compounds **3**, **17**, donepezil, **20**, ethylene glycol, **3** + ethylene glycol, and **15a** (**A**) and compounds **3** + donepezil, **3** + **20**, **17** + donepezil, **17** + **20**, donepezil + ethylene glycol, and **3** + donepezil + ethylene glycol (**B**). Tween 20 (assembly inhibition) and 2,5-DHBA (2,5-dihydroxybenzoic acid) (dissociator) were used as controls. These experiments were performed in duplicate (*n* = 1 independent experiment). The error bars represent standard deviations (SDEV).

Having demonstrated the ability of 1,3- and 1,4-chalcone-donepezil hybrids **15a**–**15d** and **16a**–**16d** to inhibit bioAβ_42_ oligomer assembly (Figure 3), we wanted to investigate the importance of the covalent linkage of the chalcone and donepezil fragments on bioAβ_42_ assembly. To do so, we tested chalcone **3**, chalcone **17**, donepezil, and its analogue **20** alone for their ability to inhibit bioAβ_42_ oligomer assembly (Figure 6A). We found that chalcone **3**, chalcone **17**, donepezil, and its analogue **20** did not inhibit bioAβ_42_ assembly. Next, we explored 1:1 mixtures of chalcones **3** or **17** with donepezil or its analogue **20** against bioAβ_42_ oligomer assembly (Figure 6B). None of the above combinations prevented bioAβ_42_ oligomerization. Combining ethylene glycol linker to chalcone **3**, donepezil, and chalcone **3** with donepezil in either 1:1 or 1:1:1 mixtures also did not result in inhibition of bioAβ_42_ oligomer assembly. In comparison, the 1,3-chalcone-donepezil hybrid **15a** was successful in preventing the self-assembly of bioAβ_42_. These data confirm the importance of having an actual covalent linkage between the chalcones and donepezil for inhibiting bioAβ_42_ oligomer assembly.

**Figure 7:**
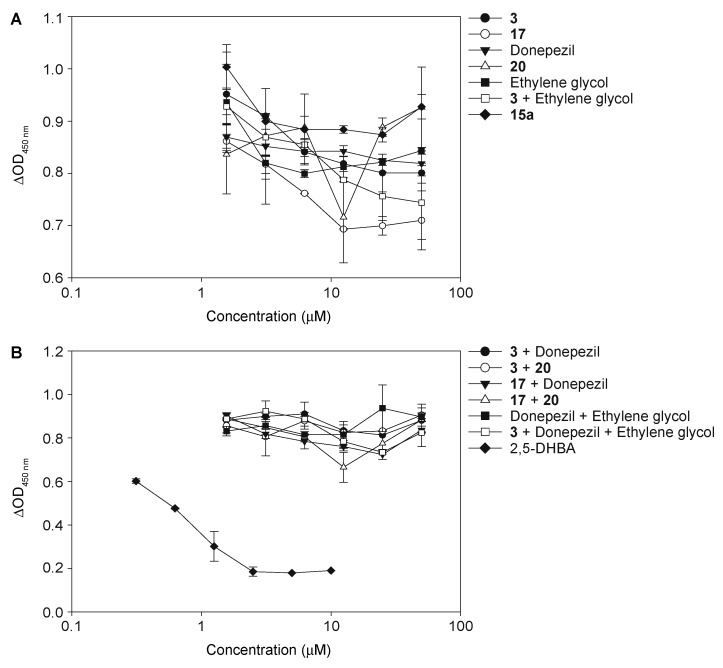
The measurement of biotinyl-Aβ_(1–42)_ (bioAβ_42_) oligomer dissociation of compounds **3**, **17**, donepezil, **20**, ethylene glycol, **3** + ethylene glycol, and **15a** (**A**) and compounds **3** + donepezil, **3** + **20**, **17** + donepezil, **17** + **20**, donepezil + ethylene glycol, and **3** + donepezil + ethylene glycol (**B**). 2,5-DHBA (2,5-dihydroxybenzoic acid) was used as a positive dissociator control. These experiments were performed in duplicate (*n* = 1 independent experiment). The error bars represent standard deviations (SDEV).

Since we had shown in a previous publication that some chalcones could dissociate preformed bioAβ_42_ oligomers [58], we wanted to investigate if the covalent attachment of the various chalcones to donepezil, as in hybrids **15a**–**15f** and **16a**–**16f**, was responsible for the lack of ability of these hybrids to dissociate the oligomers. We wanted to determine if chalcones **3** or **17**, or donepezil, or its analogue **20** alone could dissociate the preformed bioAβ_42_ oligomers (Figure 7A). We also desired to see if combining chalcones **3** or **17** in a 1:1 mixture with donepezil or its analogue **20** would result in dissociation of bioAβ_42_ oligomers (Figure 7B). We found that chalcones **3** and **17** alone, as well as donepezil and its analogue **20**, did not dissociate the preformed oligomers. Neither did the various combinations tested. We also explored the addition of the ethylene glycol linker to donepezil, chalcone **3**, and donepezil with chalcone **3** in 1:1 or 1:1:1 mixtures. We found that these combinations also did not allow for dissociation of preformed bioAβ_42_ oligomers. These data highlight that these specific chalcones **3** and **17** with a hydroxyl moiety at position 3, unlike the previously published chalcone with an hydroxyl at position 2 [58], do not dissociate preformed bioAβ_42_ oligomers. This was not completely surprising, as not all chalcones in our previous study were able to do so.

### 2.5. Molecular Modeling

**Figure 8:**
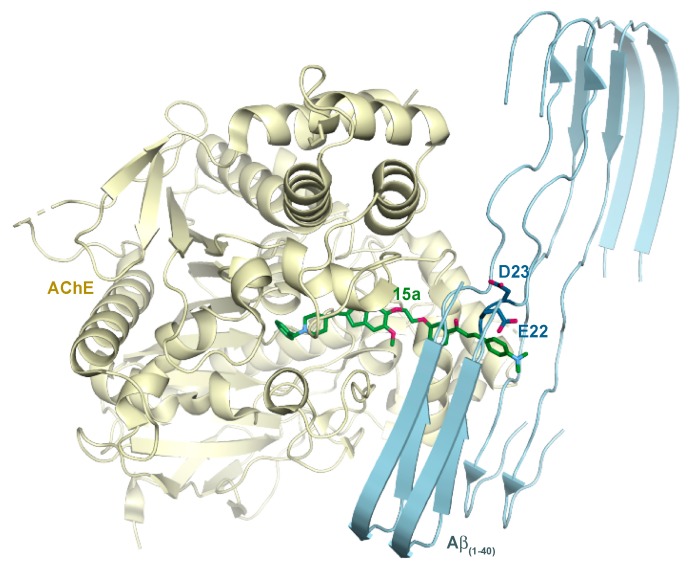
A structural model of the *Hs*AChE-**15a**-Aβ fibril complex. The *Hs*AChE is shown in pale yellow, a two-layer Aβ (residues 1–40) fibril is in light blue, and **15a** is in green. The acidic residues Glu22 and Asp23 of Aβ that may favorably interact with the dimethyl amino group of **15a** are shown as dark blue sticks. The length of the linker of **15a** can span no more than two β-strand layers, as shown in the figure. Note: Oxygen and nitrogen atoms are in red and blue, respectively.

In order to understand how our chalcone-donepezil hybrid molecules may bind simultaneously the AChE enzyme and an Aβ fibril, we built a model of a *Hs*AChE-**15a**-Aβ fibril complex using a crystal structure of the *Hs*AChE-donepezil complex (PDB ID: 4EY7 [66]) and a cryo-EM structure of an Aβ (residues 1–40) fibril (PDB ID: 6SHS [67]) (Figure 8). The donepezil moiety of the hybrid was unambiguously defined by the bound donepezil in the former structure. In searching for a potential binding site for the chalcone-containing side of **15a** in the Aβ fibril, we noticed that Glu22 and Asp23 formed a negatively-charged surface patch that could interact favorably with the terminal positively charged dimethyl amino group of **15a**. The largely hydrophobic channel (lined with side chain of Phe19, Ala21, and Val24) would fit and favorably interact with the hydrophobic regions of the chalcone. The oxygen atom of the carbonyl group of chalcone may form hydrogen bonds with amide nitrogen atoms in this region of the Aβ, where β-sheets are highly distorted. Notably, the linker of **15a** can span no more than two β-strand layers, as shown in Figure 8. In this model, the two β-strands are book-ended by the *Hs*AChE on one side and the terminal dimethyl amino group on the other. These interactions would prevent propagation of the filament or even potentially cause a disassembly of a larger filament into at most two-strand-thick assemblies. It is obvious that in this model, a longer linker of the hybrid compound would allow a thicker filament to form; therefore, it would not be as potent in preventing filament propagation.

## 3. Materials and Methods

### 3.1. Materials and Instrumentation

All chemicals were purchased from Sigma-Aldrich (St. Louis, MO, USA), Alfa Aesar (Ward Hill, MA, USA), and AK scientific (Union City, CA, USA), and used without further purification. Chemical reactions were monitored by thin layer chromatography (TLC) using Merck Silica gel 60 F_254_ plates. Visualization was achieved using UV light and a ceric molybdate stain (5 g (NH_4_)_2_Ce(NO_3_)_6_, 120 g (NH_4_)_6_Mo_7_O_24_·4H_2_O, 80 mL H_2_SO_4_, and 720 mL H_2_O). ^1^H and ^13^C NMR spectra were recorded at 400 and 100 MHz (or 125 MHz), respectively, on a Varian 400 MHz spectrometer (MR400) (or a Varian 500 MHz spectrometer; VNMRS 500), using the indicated deuterated solvents. Chemical shifts (*δ*) are given in parts per million (ppm). Coupling constants (*J*) are given in Hertz (Hz), and conventional abbreviations used for signal shape are as follows: s = singlet; d = doublet; t = triplet; m = multiplet; dd = doublet of doublets; ddd = doublet of doublet of doublets; br s = broad singlet; dt = doublet of triplets. Liquid chromatography-mass spectrometry (LCMS) was carried out using an Agilent 1200 series Quaternary LC system equipped with a diode array detector, and Eclipse XDB-C18 column (250 mm × 4.6 mm, 5 μm). LCMS [M + H]^+^ signals were consistent with the expected molecular weights for all of the reported compounds. Tween 20 was purchased from Sigma-Aldrich (cat # P7949). *N*-biotinyl Aβ_(1–42)_ (bioAβ_42_) was purchased from Anaspec (cat # AS-23523-05; Fremont, CA, USA). ELISA plates (Costar 9018), NeutrAvidin^TM^ (Promega), adhesive film (NUNC), polypropylene 96-well plates (Costar 3365), and polypropylene Eppendorf tubes (Fisher 02-681-248) used for bioAβ_42_ oligomer assembly and dissociation assays were all purchased from Fisher Scientific (Pittsburgh, PA, USA). Plates were washed on a BioTek ELx50 plate washer (BioTek (Winooski, VT, USA)) and absorbance was read on a BioTek HT Synergy plate reader.

### 3.2. Synthesis of Compounds ***3***–***20***

*(E)-3-(4-(Dimethylamino)Phenyl)-1-(3-Hydroxyphenyl)Prop-2-En-1-One* (**3**) (SGT9). The known compound **3** was prepared as previously described [68]. A solution of compounds **1** (68 mg, 0.5 mmol) and **2** (75 mg, 0.5 mmol) in EtOH (1.5 mL) was treated with NaOH pellets (400 mg, 10.0 mmol). The reaction was stirred at room temperature (rt) overnight till completion. Most of the solvent was then removed and 1 N aqueous HCl was added. The precipitate was filtered to give the known compound **3** (110 mg, 82%) (R*_f_* 0.26 in Hexanes:EtOAc/3:1) as an orange solid: ^1^H NMR (400 MHz, CDCl_3_, Figure S1, which matches the lit. [68]) *δ* 7.78 (d, *J* = 15.6 Hz, 1H, HC=CH-Ph), 7.56–7.50 (m, 4H, aromatic), 7.35 (t, *J* = 8.0 Hz, 1H, aromatic), 7.29 (d, *J* = 15.6 Hz, 1H, HC=CH-Ph), 7.03 (dd, **J*_1_* = 8.0 Hz, **J*_2_* = 2.0 Hz, 1H, aromatic), 6.69 (d, *J* = 9.2 Hz, 2H, aromatic), 5.30 (s, 1H, OH), 3.04 (s, 6H, N(CH_3_)_2_).

*(E)-1-(3-(2-Bromoethoxy)Phenyl)-3-(4-(Dimethylamino)Phenyl)Prop-2-En-1-One* (**4a**) (SGT653). A solution of compound **3** (80 mg, 0.30 mmol) and K_2_CO_3_ (165 mg, 1.20 mmol) in anhydrous MeCN (5 mL) was treated with 1,2-dibromoethane (0.10 mL, 1.20 mmol) and the resulting mixture was refluxed overnight. The solvent was then removed and the obtained crude product was purified by column chromatography (SiO_2_ gel, pure Hexanes to Hexanes:EtOAc/4:1; R*_f_* 0.38 in Hexanes:EtOAc/3:1) to yield compound **4a** (43 mg, 38%) as a red solid: ^1^H NMR (400 MHz, CDCl_3_, Figure S2) *δ* 7.78 (d, *J* = 15.6 Hz, 1H, HC=CH-Ph), 7.61 (d, *J* = 8.0 Hz, 1H, aromatic), 7.55–7.52 (m, 3H, aromatic), 7.39 (t, *J* = 8.0 Hz, 1H, aromatic), 7.29 (d, *J* = 15.6 Hz, 1H, HC=CH-Ph), 7.10 (dd, **J*_1_* = 8.0 Hz, **J*_2_* = 2.8 Hz, 1H, aromatic), 6.70 (d, *J* = 8.8 Hz, 2H, aromatic), 4.37 (t, *J* = 6.0 Hz, 2H, BrCH_2_CH_2_OPh), 3.66 (t, *J* = 6.0 Hz, 2H, BrCH_2_CH_2_OPh), 3.04 (s, 6H, N(CH_3_)_2_); ^13^C NMR (100 MHz, CDCl_3_, Figure S3) *δ* 190.1, 158.3, 152.1, 146.0, 140.6, 130.5 (2 carbons), 129.5, 122.5, 121.5, 119.3, 116.7, 113.6, 111.8 (2 carbons), 68.0, 40.1 (2 carbons), 29.1; *m*/*z* calcd for C_19_H_20_BrNO_2_ 373.1; found 374.0 [M + H]^+^.

*(E)-1-(3-(4-Bromobutoxy)Phenyl)-3-(4-(Dimethylamino)Phenyl)Prop-2-En-1-One* (**4b**) (SGT47). A solution of compound **3** (50 mg, 0.19 mmol) and K_2_CO_3_ (52 mg, 0.37 mmol) in anhydrous MeCN (5 mL) was treated with 1,4-dibromobutane (0.04 mL, 0.37 mmol) and the resulting mixture was stirred at 50 °C for 4 h. The solvent was then removed and the obtained crude product was purified by column chromatography (SiO_2_ gel, pure Hexanes to Hexanes:EtOAc/4:1; R*_f_* 0.60 in Hexanes:EtOAc/3:1) to yield compound **4b** (64 mg, 85%) as a red solid: ^1^H NMR (400 MHz, CDCl_3_, Figure S4) *δ* 7.77 (d, *J* = 15.6 Hz, 1H, HC=CH-Ph), 7.57-7.50 (m, 4H, aromatic), 7.36 (t, *J* = 8.4 Hz, 1H, aromatic), 7.29 (d, *J* = 15.6 Hz, 1H, HC=CH-Ph), 7.06 (d, *J* = 8.4 Hz, 1H, aromatic), 6.69 (d, *J* = 8.4 Hz, 2H, aromatic), 4.06 (t, *J* = 6.0 Hz, 2H, BrCH_2_CH_2_CH_2_CH_2_OPh), 3.49 (t, *J* = 6.4 Hz, 2H, BrCH_2_CH_2_CH_2_CH_2_OPh), 3.03 (s, 6H, N(CH_3_)_2_), 2.08 (p, *J* = 7.2 Hz, 2H, BrCH_2_CH_2_CH_2_CH_2_OPh), 1.96 (p, *J* = 7.2 Hz, 2H, BrCH_2_CH_2_CH_2_CH_2_OPh); ^13^C NMR (100 MHz, CDCl_3_, Figure S5) *δ* 190.3, 159.0, 152.0, 145.8, 140.5, 130.4 (2 carbons), 129.4, 122.7, 120.9, 119.0, 117.0, 113.3, 111.9 (2 carbons), 67.0, 40.2 (2 carbons), 33.4, 29.4, 27.8; *m*/*z* calcd for C_21_H_24_BrNO_2_ 401.1; found 402.0 [M + H]^+^.

*(E)-1-(3-((6-Bromohexyl)Oxy)Phenyl)-3-(4-(Dimethylamino)Phenyl)Prop-2-En-1-One* (**4c**) (SGT684). A solution of compound **3** (100 mg, 0.37 mmol) and K_2_CO_3_ (207 mg, 1.50 mmol) in anhydrous MeCN (5 mL) was treated with 1,6-dibromohexane (0.23 mL, 1.50 mmol) and the resulting mixture was stirred at 50 °C overnight. The solvent was then removed and the obtained crude product was purified by column chromatography (SiO_2_ gel, pure Hexanes to Hexanes:EtOAc/4:1; R*_f_* 0.60 in Hexanes:EtOAc/3:1) to yield compound **4c** (143 mg, 89%) as an orange solid: ^1^H NMR (400 MHz, CDCl_3_, Figure S6) *δ* 7.78 (d, *J* = 15.6 Hz, 1H, HC=CH-Ph), 7.57-7.50 (m, 4H, aromatic), 7.36 (t, *J* = 8.0 Hz, 1H, aromatic), 7.30 (d, *J* = 15.6 Hz, 1H, HC=CH-Ph), 7.07 (d, *J* = 8.0 Hz, 1H, aromatic), 6.68 (d, *J* = 8.4 Hz, 2H, aromatic), 4.02 (t, *J* = 6.4 Hz, 2H, BrCH_2_CH_2_(CH_2_)_2_CH_2_CH_2_OPh), 3.42 (t, *J* = 6.8 Hz, 2H, BrCH_2_CH_2_(CH_2_)_2_CH_2_CH_2_OPh), 3.03 (s, 6H, N(CH_3_)_2_), 1.89 (br p, *J* = 6.4 Hz, 2H, BrCH_2_CH_2_(CH_2_)_2_CH_2_CH_2_OPh), 1.81 (br p, *J* = 5.6 Hz, 2H, BrCH_2_CH_2_(CH_2_)_2_CH_2_CH_2_OPh), 1.51 (m, 4H, BrCH_2_CH_2_(CH_2_)_2_CH_2_CH_2_OPh); ^13^C NMR (100 MHz, CDCl_3_, Figure S7) *δ* 190.4, 159.2, 152.0, 145.8, 140.4, 130.4 (2 carbons), 129.4, 122.6, 120.7, 119.0, 116.9, 113.4, 111.8 (2 carbons), 67.9, 40.2 (2 carbons), 33.9, 32.7, 29.0, 27.9, 25.3; *m*/*z* calcd for C_23_H_28_BrNO_2_ 429.1; found 430.1 [M + H]^+^.

*(E)-1-(3-((8-Bromooctyl)Oxy)Phenyl)-3-(4-(Dimethylamino)Phenyl)Prop-2-En-1-One* (**4d**) (SGT685). A solution of compound **3** (100 mg, 0.37 mmol) and K_2_CO_3_ (207 mg, 1.50 mmol) in anhydrous MeCN (5 mL) was treated with 1,8-dibromooctane (0.28 mL, 1.50 mmol) and the resulting mixture was stirred at 50 °C overnight. The solvent was then removed and the obtained crude product was purified by column chromatography (SiO_2_ gel, pure Hexanes to Hexanes:EtOAc/4:1; R*_f_* 0.60 in Hexanes:EtOAc/3:1) to yield compound **4d** (139 mg, 81%) as a yellow solid: ^1^H NMR (400 MHz, CDCl_3_, Figure S8) *δ* 7.78 (d, *J* = 15.6 Hz, 1H, HC=CH-Ph), 7.57-7.50 (m, 4H, aromatic), 7.36 (t, *J* = 8.0 Hz, 1H, aromatic), 7.30 (d, *J* = 15.6 Hz, 1H, HC=CH-Ph), 7.07 (d, *J* = 8.0 Hz, 1H, aromatic), 6.68 (d, *J* = 8.4 Hz, 2H, aromatic), 4.01 (t, *J* = 6.4 Hz, 2H, BrCH_2_CH_2_(CH_2_)_4_CH_2_CH_2_OPh), 3.40 (t, *J* = 6.4 Hz, 2H, BrCH_2_CH_2_(CH_2_)_4_CH_2_CH_2_OPh), 3.03 (s, 6H, N(CH_3_)_2_), 1.85 (p, *J* = 7.6 Hz, 2H, BrCH_2_CH_2_(CH_2_)_4_CH_2_CH_2_OPh), 1.79 (p, *J* = 7.6 Hz, 2H, BrCH_2_CH_2_(CH_2_)_4_CH_2_CH_2_OPh), 1.40–1.50 (m, 4H, BrCH_2_CH_2_(CH_2_)_4_CH_2_CH_2_OPh), 1.36 (m, 4H, BrCH_2_CH_2_(CH_2_)_4_CH_2_CH_2_OPh); ^13^C NMR (100 MHz, CDCl_3_, Figure S9) *δ* 190.4, 159.2, 152.0, 145.8, 140.4, 130.4 (2 carbons), 129.3, 122.6, 120.6, 119.0, 116.9, 113.4, 111.8 (2 carbons), 68.1, 40.1 (2 carbons), 34.1, 32.8, 29.2 (2 carbons), 28.7, 28.1, 25.9; *m*/*z* calcd for C_25_H_32_BrNO_2_ 457.2; found 458.0 [M + H]^+^.

*(E)-1-(3-((10-Bromodecyl)Oxy)Phenyl)-3-(4-(Dimethylamino)Phenyl)Prop-2-En-1-One* (**4e**) (SGT686). A solution of compound **3** (100 mg, 0.37 mmol) and K_2_CO_3_ (207 mg, 1.50 mmol) in anhydrous MeCN (5 mL) was treated with 1,10-dibromodecane (0.34 mL, 1.50 mmol) and the resulting mixture was stirred at 50 °C overnight. The solvent was then removed and the obtained crude product was purified by column chromatography (SiO_2_ gel, pure Hexanes to Hexanes:EtOAc/4:1; R*_f_* 0.60 in Hexanes:EtOAc/3:1) to yield compound **4e** (160 mg, 89%) as a yellow solid: ^1^H NMR (400 MHz, CDCl_3_, Figure S10) *δ* 7.78 (d, *J* = 15.2 Hz, 1H, HC=CH-Ph), 7.57–7.50 (m, 4H, aromatic), 7.36 (t, *J* = 8.0 Hz, 1H, aromatic), 7.30 (d, *J* = 15.2 Hz, 1H, HC=CH-Ph), 7.07 (d, *J* = 8.4 Hz, 1H, aromatic), 6.68 (d, *J* = 8.4 Hz, 2H, aromatic), 4.01 (t, *J* = 6.8 Hz, 2H, BrCH_2_CH_2_(CH_2_)_6_CH_2_CH_2_OPh), 3.39 (t, *J* = 6.8 Hz, 2H, BrCH_2_CH_2_(CH_2_)_6_CH_2_CH_2_OPh), 3.03 (s, 6H, N(CH_3_)_2_), 1.81 (m, 4H), 1.44 (m, 4H), 1.30 (m, 8H); ^13^C NMR (100 MHz, CDCl_3_, Figure S11) *δ* 190.4, 159.3, 152.0, 145.8, 140.4, 130.4 (2 carbons), 129.3, 122.6, 120.6, 119.0, 116.9, 113.4, 111.8 (2 carbons), 68.1, 40.2 (2 carbons), 34.1, 32.8, 29.44, 29.35, 29.32, 29.2, 28.7, 28.1, 26.0; *m*/*z* calcd for C_27_H_36_BrNO_2_ 485.2; found 486.0 [M + H]^+^.

*(E)-1-(3-((12-Bromododecyl)Oxy)Phenyl)-3-(4-(Dimethylamino)Phenyl)Prop-2-En-1-One* (**4f**) (SGT633). A solution of compound **3** (80 mg, 0.30 mmol) and K_2_CO_3_ (165 mg, 1.20 mmol) in anhydrous MeCN (5 mL) was treated with 1,12-dibromododecane (392 mg, 1.20 mmol) and the resulting mixture was refluxed overnight. The solvent was then removed and the obtained crude product was purified by column chromatography (SiO_2_ gel, pure Hexanes to Hexanes:EtOAc/4:1; R*_f_* 0.53 in Hexanes:EtOAc/3:1) to yield compound **4f** (122 mg, 79%) as an orange solid: ^1^H NMR (400 MHz, CDCl_3_, Figure S12) *δ* 7.77 (d, *J* = 15.6 Hz, 1H, HC=CH-Ph), 7.56–7.50 (m, 4H, aromatic), 7.36 (t, *J* = 8.0 Hz, 1H, aromatic), 7.30 (d, *J* = 15.6 Hz, 1H, HC=CH-Ph), 7.07 (dd, **J*_1_* = 8.0 Hz, **J*_2_* = 2.0 Hz, 1H, aromatic), 6.71 (d, *J* = 8.4 Hz, 2H, aromatic), 4.02 (t, *J* = 6.8 Hz, 2H, BrCH_2_(CH_2_)_10_CH_2_OPh), 3.39 (t, *J* = 6.8 Hz, 2H, BrCH_2_(CH_2_)_10_CH_2_OPh), 3.04 (s, 6H, N(CH_3_)_2_), 1.87–1.75 (m, 4H), 1.47–1.27 (m, 16H); ^13^C NMR (100 MHz, CDCl_3_, Figure S13) *δ* 190.4, 159.3, 152.0, 145.8, 140.4, 130.4 (2 carbons), 129.3, 122.7, 120.6, 119.0, 117.0, 113.4, 111.8 (2 carbons), 68.2, 40.1 (2 carbons), 34.1, 32.8, 29.5 (3 carbons), 29.4, 29.3, 29.2, 28.7, 28.2, 26.0; *m*/*z* calcd for C_29_H_40_BrNO_2_ 513.2; found 514.3 [M + H]^+^.

*(E)-3-(4-(Dimethylamino)Phenyl)-1-(4-Hydroxyphenyl)Prop-2-En-1-One* (**6**) (SGT649). The known compound **6** was prepared as previously described [69]. A solution of compounds **5** (0.50 g, 3.67 mmol) and **2** (0.55 g, 3.67 mmol) in EtOH (10 mL) was treated with NaOH pellets (2.94 g, 73.4 mmol). The reaction was stirred at rt overnight till completion. Most of the solvent was then removed and 1 N aqueous HCl was added. The precipitate was filtered to give the known compound **6** (0.43 g, 44%) (R*_f_* 0.20 in Hexanes:EtOAc/2:1) as a red solid: ^1^H NMR (400 MHz, CDCl_3_, Figure S14, which matches the lit. [69]) *δ* 7.97 (d, *J* = 8.4 Hz, 2H, aromatic), 7.77 (d, *J* = 15.6 Hz, 1H, HC=CH-Ph), 7.54 (d, *J* = 8.8 Hz, 2H, aromatic), 7.33 (d, *J* = 15.6 Hz, 1H, HC=CH-Ph), 6.90 (d, *J* = 8.4 Hz, 2H, aromatic), 6.72 (d, *J* = 8.8 Hz, 2H, aromatic), 5.42 (s, 1H, OH), 3.04 (s, 6H, N(CH_3_)_2_).

*(E)-1-(4-(2-Bromoethoxy)Phenyl)-3-(4-(Dimethylamino)Phenyl)Prop-2-En-1-One* (**7a**) (SGT652). A solution of **6** (80 mg, 0.30 mmol) and K_2_CO_3_ (165 mg, 1.20 mmol) in anhydrous MeCN (5 mL) was treated with 1,2-dibromoethane (0.10 mL, 1.20 mmol) and the resulting mixture was refluxed overnight. The solvent was then removed and the obtained crude product was purified by column chromatography (SiO_2_ gel, pure Hexanes to Hexanes:EtOAc/4:1; R*_f_* 0.31 in Hexanes:EtOAc/3:1) to yield compound **7a** (90 mg, 80%) as a yellow solid: ^1^H NMR (400 MHz, CDCl_3_, Figure S15) *δ* 8.01 (d, *J* = 8.8 Hz, 2H, aromatic), 7.77 (d, *J* = 15.6 Hz, 1H, HC=CH-Ph), 7.55 (d, *J* = 8.8 Hz, 2H, aromatic), 7.34 (d, *J* = 15.6 Hz, 1H, HC=CH-Ph), 6.97 (d, *J* = 8.8 Hz, 2H, aromatic), 6.75 (d, *J* = 8.8 Hz, 2H, aromatic), 4.36 (t, *J* = 6.0 Hz, 2H, BrCH_2_CH_2_OPh), 3.66 (t, *J* = 6.0 Hz, 2H, BrCH_2_CH_2_OPh), 3.04 (s, 6H, N(CH_3_)_2_); ^13^C NMR (100 MHz, CDCl_3_, Figure S16) *δ* 188.8, 161.3, 151.9, 145.1, 132.5, 130.6 (2 carbons), 130.3 (2 carbons), 122.8, 116.5, 114.2 (2 carbons), 111.8 (2 carbons), 67.8, 40.1 (2 carbons), 28.7; *m*/*z* calcd for C_19_H_20_BrNO_2_ 373.1; found 374.0 [M + H]^+^.

*(E)-1-(4-(4-Bromobutoxy)Phenyl)-3-(4-(Dimethylamino)Phenyl)Prop-2-En-1-One* (**7b**) (SGT648). A solution of compound **6** (80 mg, 0.30 mmol) and K_2_CO_3_ (83 mg, 0.60 mmol) in anhydrous MeCN (5 mL) was treated with 1,4-dibromobutane (0.07 mL, 0.60 mmol) and the resulting mixture was refluxed overnight. The solvent was then removed and the obtained crude product was purified by column chromatography (SiO_2_ gel, pure Hexanes to Hexanes:EtOAc/4:1; R*_f_* 0.42 in Hexanes:EtOAc/3:1) to yield compound **7b** (56 mg, 47%) as an orange solid: ^1^H NMR (400 MHz, CDCl_3_, Figure S17) *δ* 8.00 (d, *J* = 8.8 Hz, 2H, aromatic), 7.77 (d, *J* = 15.6 Hz, 1H, HC=CH-Ph), 7.54 (d, *J* = 8.4 Hz, 2H, aromatic), 7.34 (d, *J* = 15.6 Hz, 1H, HC=CH-Ph), 6.94 (d, *J* = 8.8 Hz, 2H, aromatic), 6.70 (d, *J* = 8.4 Hz, 2H, aromatic), 4.07 (t, *J* = 6.4 Hz, 2H, BrCH_2_CH_2_CH_2_CH_2_OPh), 3.49 (t, *J* = 6.4 Hz, 2H, BrCH_2_CH_2_CH_2_CH_2_OPh), 3.03 (s, 6H, N(CH_3_)_2_), 2.08 (p, *J* = 6.4 Hz, 2H, BrCH_2_CH_2_CH_2_CH_2_OPh), 1.97 (p, *J* = 6.4 Hz, 2H, BrCH_2_CH_2_CH_2_CH_2_OPh); ^13^C NMR (100 MHz, CDCl_3_, Figure S18) *δ* 188.8, 162.2, 151.8, 144.9, 131.8, 130.5 (2 carbons), 130.2 (2 carbons), 123.0, 116.7, 114.1 (2 carbons), 111.9 (2 carbons), 67.0, 40.2 (2 carbons), 33.3, 29.3, 27.8; *m*/*z* calcd for C_21_H_24_BrNO_2_ 401.1; found 402.0 [M + H]^+^.

*(E)-1-(4-((6-Bromohexyl)Oxy)Phenyl)-3-(4-(Dimethylamino)Phenyl)Prop-2-En-1-One* (**7c**) (SGT691). A solution of compound **6** (50 mg, 0.19 mmol) and K_2_CO_3_ (103 mg, 0.75 mmol) in anhydrous MeCN (5 mL) was treated with 1,6-dibromohexane (0.12 mL, 0.75 mmol) and the resulting mixture was stirred at 50 °C overnight. The solvent was then removed and the obtained crude product was purified by column chromatography (SiO_2_ gel, pure Hexanes to Hexanes:EtOAc/4:1; R*_f_* 0.33 in Hexanes:EtOAc/4:1) to yield compound **7c** (56 mg, 70%) as an orange solid: ^1^H NMR (400 MHz, CDCl_3_, Figure S19) *δ* 8.00 (d, *J* = 8.4 Hz, 2H, aromatic), 7.77 (d, *J* = 15.6 Hz, 1H, HC=CH-Ph), 7.54 (d, *J* = 8.4 Hz, 2H, aromatic), 7.35 (d, *J* = 15.6 Hz, 1H, HC=CH-Ph), 6.94 (d, *J* = 8.4 Hz, 2H, aromatic), 6.71 (d, *J* = 8.4 Hz, 2H, aromatic), 4.03 (t, *J* = 6.4 Hz, 2H, BrCH_2_CH_2_CH_2_CH_2_OPh), 3.42 (t, *J* = 6.4 Hz, 2H, BrCH_2_CH_2_CH_2_CH_2_OPh), 3.03 (s, 6H, N(CH_3_)_2_), 1.89 (br p, *J* = 6.4 Hz, 2H, BrCH_2_CH_2_(CH_2_)_2_CH_2_CH_2_OPh), 1.82 (br p, *J* = 6.4 Hz, 2H, BrCH_2_CH_2_(CH_2_)_2_CH_2_CH_2_OPh), 1.51 (m, 4H, BrCH_2_CH_2_(CH_2_)_2_CH_2_CH_2_OPh; ^13^C NMR (100 MHz, (CD_3_)_2_SO, Figure S20) *δ* 187.4, 162.6, 152.3, 144.7, 131.03 (2 carbons), 130.94 (2 carbons), 122.6, 116.5, 114.7 (2 carbons), 112.2 (2 carbons), 68.1, 65.0, 35.6 (2 carbons), 32.6, 28.8, 27.7, 25.0; *m*/*z* calcd for C_23_H_28_BrNO_2_ 429.1; found 430.0 [M + H]^+^.

*(E)-1-(4-((8-Bromooctyl)Oxy)Phenyl)-3-(4-(Dimethylamino)Phenyl)Prop-2-En-1-One* (**7d**) (SGT692). A solution of compound **6** (50 mg, 0.19 mmol) and K_2_CO_3_ (103 mg, 0.75 mmol) in anhydrous MeCN (5 mL) was treated with 1,8-dibromooctane (0.14 mL, 0.75 mmol) and the resulting mixture was stirred at 50 °C overnight. The solvent was then removed and the obtained crude product was purified by column chromatography (SiO_2_ gel, pure Hexanes to Hexanes:EtOAc/4:1; R*_f_* 0.33 in Hexanes:EtOAc/4:1) to yield compound **7d** (47 mg, 55%) as a yellow solid: ^1^H NMR (400 MHz, CDCl_3_, Figure S21) *δ* 8.00 (d, *J* = 8.8 Hz, 2H, aromatic), 7.77 (d, *J* = 15.2 Hz, 1H, HC=CH-Ph), 7.54 (d, *J* = 8.8 Hz, 2H, aromatic), 7.35 (d, *J* = 15.2 Hz, 1H, HC=CH-Ph), 6.94 (d, *J* = 8.8 Hz, 2H, aromatic), 6.70 (d, *J* = 8.4 Hz, 2H, aromatic), 4.02 (t, *J* = 6.4 Hz, 2H, BrCH_2_CH_2_(CH_2_)_4_CH_2_CH_2_OPh), 3.40 (t, *J* = 6.4 Hz, 2H, BrCH_2_CH_2_(CH_2_)_4_CH_2_CH_2_OPh), 3.03 (s, 6H, N(CH_3_)_2_), 1.85 (p, *J* = 6.8 Hz, 2H, BrCH_2_CH_2_(CH_2_)_4_CH_2_CH_2_OPh), 1.80 (p, *J* = 6.8 Hz, 2H, BrCH_2_CH_2_(CH_2_)_4_CH_2_CH_2_OPh), 1.40–1.50 (m, 4H), 1.36 (m, 4H); ^13^C NMR (100 MHz, CDCl_3_, Figure S22) *δ* 188.8, 162.5, 144.7, 131.6, 130.5 (2 carbons), 130.2 (2 carbons), 116.9, 114.1 (2 carbons), 112.1 (2 carbons), 68.1, 40.3 (2 carbons), 34.0, 32.7, 29.14, 29.07, 28.6, 28.1, 25.9; *m*/*z* calcd for C_25_H_32_BrNO_2_ 457.2; found 458.4 [M + H]^+^.

*(E)-1-(4-((10-Bromodecyl)Oxy)Phenyl)-3-(4-(Dimethylamino)Phenyl)Prop-2-En-1-One* (**7e**) (SGT693). A solution of compound **6** (50 mg, 0.19 mmol) and K_2_CO_3_ (103 mg, 0.75 mmol) in anhydrous MeCN (5 mL) was treated with 1,10-dibromodecane (0.17 mL, 0.75 mmol) and the resulting mixture was stirred at 50 °C overnight. The solvent was then removed and the obtained crude product was purified by column chromatography (SiO_2_ gel, pure Hexanes to Hexanes:EtOAc/4:1; R*_f_* 0.41 in Hexanes:EtOAc/4:1) to yield compound **7e** (41 mg, 46%) as a yellow solid: ^1^H NMR (400 MHz, CDCl_3_, Figure S23) *δ* 8.00 (d, *J* = 8.4 Hz, 2H, aromatic), 7.77 (d, *J* = 15.2 Hz, 1H, HC=CH-Ph), 7.54 (d, *J* = 8.8 Hz, 2H, aromatic), 7.35 (d, *J* = 15.2 Hz, 1H, HC=CH-Ph), 6.94 (d, *J* = 8.4 Hz, 2H, aromatic), 6.72 (d, *J* = 8.4 Hz, 2H, aromatic), 4.02 (t, *J* = 6.8 Hz, 2H, BrCH_2_CH_2_(CH_2_)_6_CH_2_CH_2_OPh), 3.40 (t, *J* = 6.8 Hz, 2H, BrCH_2_CH_2_(CH_2_)_6_CH_2_CH_2_OPh), 3.03 (s, 6H, N(CH_3_)_2_), 1.84 (p, *J* = 7.6 Hz, 2H, BrCH_2_CH_2_(CH_2_)_6_CH_2_CH_2_OPh), 1.79 (p, *J* = 6.8 Hz, 2H, BrCH_2_CH_2_(CH_2_)_4_CH_2_CH_2_OPh), 1.43 (m, 4H), 1.30 (m, 8H); ^13^C NMR (100 MHz, CDCl_3_, Figure S24) *δ* 188.8, 162.6, 144.7, 131.5, 130.5 (2 carbons), 130.2 (2 carbons), 116.9, 114.1 (2 carbons), 112.0 (2 carbons), 68.1, 40.3 (2 carbons), 34.0, 32.8, 29.4, 29.32, 29.28, 29.1, 28.7, 28.1, 25.9; *m*/*z* calcd for C_27_H_36_BrNO_2_ 485.2; found 486.3 [M + H]^+^.

*(E)-1-(4-((12-Bromododecyl)Oxy)Phenyl)-3-(4-(Dimethylamino)Phenyl)Prop-2-En-1-One* (**7f**) (SGT654). A solution of compound **6** (80 mg, 0.30 mmol) and K_2_CO_3_ (165 mg, 1.20 mmol) in anhydrous MeCN (5 mL) was treated with 1,12-dibromododecane (392 mg, 1.20 mmol) and the resulting mixture was refluxed overnight. The solvent was then removed and the obtained crude product was purified by column chromatography (SiO_2_ gel, pure Hexanes to Hexanes:EtOAc/4:1; R*_f_* 0.47 in Hexanes:EtOAc/3:1) to yield compound **7f** (132 mg, 86%) as an orange solid: ^1^H NMR (400 MHz, CDCl_3_, Figure S25) *δ* 8.00 (d, *J* = 8.8 Hz, 2H, aromatic), 7.77 (d, *J* = 15.2 Hz, 1H, HC=CH-Ph), 7.54 (d, *J* = 8.4 Hz, 2H, aromatic), 7.35 (d, *J* = 15.2 Hz, 1H, HC=CH-Ph), 6.94 (d, *J* = 8.8 Hz, 2H, aromatic), 6.73 (d, *J* = 8.4 Hz, 2H, aromatic), 4.02 (t, *J* = 6.8 Hz, 2H, BrCH_2_(CH_2_)_10_CH_2_OPh), 3.39 (t, *J* = 6.8 Hz, 2H, BrCH_2_(CH_2_)_10_CH_2_OPh), 3.03 (s, 6H, N(CH_3_)_2_), 1.87-1.75 (m, 4H), 1.47–1.27 (m, 16H); ^13^C NMR (100 MHz, CDCl_3_, Figure S26) *δ* 188.8, 162.6, 151.8, 144.8, 131.6, 130.5 (2 carbons), 130.2 (2 carbons), 123.0, 116.8, 114.1 (2 carbons), 111.9 (2 carbons), 68.2, 40.2 (2 carbons), 34.1, 32.8, 29.5 (3 carbons), 29.4, 29.3, 29.1, 28.7, 28.2, 26.0; *m*/*z* calcd for C_29_H_40_BrNO_2_ 513.2; found 514.1 [M + H]^+^.

*3-(4-Hydroxy-3-Methoxyphenyl)Propanoic Acid* (**9**). To a solution of ferulic acid (6.0 g, 30.9 mmol) in degassed EtOAc (100 mL) was added a catalytic amount of 10% Pd/C (0.43 g). The reaction flask was then sealed with a rubber septum and freed of air. The reaction mixture was stirred at rt overnight under H_2_ atmosphere. Upon completion, the reaction mixture was filtered through a bed of celite, and concentrated to afford the known compound **9** [70] (6.1 g, quant.) as an off-white solid: ^1^H NMR (400 MHz, CDCl_3_, Figure S27, which matches the lit. [70]) *δ* 10.50 (very br s, 1H, CO_2_H), 6.82 (d, *J* = 7.6 Hz, 1H, aromatic), 6.69 (s, 1H, aromatic), 6.68 (d, *J* = 7.6 Hz, 1H, aromatic), 5.60 (very br s, 1H, OH), 3.85 (s, 3H, PhOCH_3_), 2.87 (t, *J* = 7.2 Hz, 2H, PhCH_2_CH_2_CO_2_H), 2.64 (t, *J* = 7.2 Hz, 2H, PhCH_2_CH_2_CO_2_H).

*6-Hydroxy-5-Methoxy-2,3-Dihydro-1H-Inden-1-One* (**10**). A solution of **9** (6.3 g, 32.1 mmol) in methanesulfonic acid (50 mL) was refluxed at 120 °C for 1 h. After cooling to rt, the reaction mixture was poured into ice-water, stirred for 5 min, and filtered to afford a crude dark brown solid, which was recrystallized from EtOH to afford the known compound **10** [71] (3.8 g, 67%) as a yellow solid: ^1^H NMR (400 MHz, (CD_3_)_2_SO, Figure S28, which matches the lit. [71]) *δ* 9.38 (s, 1H, OH), 7.03 (s, 1H, aromatic), 6.89 (s, 1H, aromatic), 3.83 (s, 3H, OCH_3_), 2.92 (t, *J* = 5.6 Hz, 2H, CH_2_CH_2_C=O), 2.49 (t, *J* = 5.6 Hz, 2H, CH_2_CH_2_C=O).

*6-((Tert-Butyldimethylsilyl)Oxy)-5-Methoxy-2,3-Dihydro-1H-Inden-1-One* (**11**) (SGT640). TBDMSCl (3.2 g, 21.3 mmol) was added to a solution of **10** (1.9 g, 10.7 mmol), DMAP (0.5 g, 4.3 mmol), and Et_3_N (3.0 mL, 21.3 mmol) in freshly distilled CH_2_Cl_2_ (100 mL). The reaction mixture was stirred at rt overnight before being quenched with H_2_O (100 mL). The organic layer was separated, washed with H_2_O (2 × 100 mL) and brine (100 mL), dried over anhydrous MgSO_4_, filtered, and concentrated under reduced pressure to afford a crude dark brown solid, which was purified by flash column chromatography (SiO_2_ gel, pure Hexanes to Hexanes:EtOAc/3:1, R*_f_* 0.44 in Hexanes:EtOAc/3:1) to afford a brown solid, which was further triturated in Hexanes to give compound **11** (2.8 g, 90%) as a white solid: ^1^H NMR (400 MHz, CDCl_3_, Figure S29) *δ* 7.17 (s, 1H, aromatic), 6.84 (s, 1H, aromatic), 3.87 (s, 3H, PhOCH_3_), 3.02 (app. t, *J* = 5.6 Hz, 2H, CH_2_CH_2_C=O), 2.64 (app. t, *J* = 5.6 Hz, 2H, CH_2_CH_2_C=O), 0.98 (s, 9H, SiC(CH_3_)_3_), 0.14 (s, 6H, Si(CH_3_)_2_); ^13^C NMR (100 MHz, CDCl_3_, Figure S30) *δ* 205.7, 157.5, 150.9, 145.2, 130.0, 114.1, 107.8, 55.6, 36.6, 25.62 (3 carbons), 25.56, 18.4, −4.7 (2 carbons); *m/z* calcd for C_16_H_24_O_3_Si 292.2; found 293.2 [M + H]^+^.

*(Z)-2-((1-Benzylpiperidin-4-Yl)Methylene)-6-Hydroxy-5-Methoxy-2,3-Dihydro-1H-Inden-1-One* (**13**) (SGT641). To a solution of compounds **11** (1.00 g, 3.42 mmol) and **12** (0.68 mL, 3.42 mmol) in EtOH (10 mL) was added KOH (0.5 g), and the mixture was refluxed at 65 °C. After 1 h, the reaction was analyzed by TLC (CH_2_Cl_2_:MeOH/19:1, R*_f_* 0.30 in CH_2_Cl_2_:MeOH/19:1). The reaction mixture was concentrated under reduced pressure to give a crude yellow solid, which was re-dissolved in H_2_O (10 mL). Then, 1 N aqueous HCl was then slowly added until pH 5 to give a yellow precipitate, which was recrystallized in MeCN to afford compound **13** (0.81 g, 65%) as a yellow solid: ^1^H NMR (400 MHz, CDCl_3_, Figure S31) *δ* 7.32–7.24 (m, 6H, aromatic), 6.87 (s, 1H, aromatic), 6.63 (d, *J* = 10.0 Hz, 1H, C=CH), 5.70 (br s, 1H, OH), 3.98 (s, 3H, OCH_3_), 3.56 (s, 2H), 3.51 (s, 2H), 2.91 (d, *J* = 11.6 Hz, 2H), 2.30 (m, 1H), 2.04 (t, *J* = 11.6 Hz, 2H), 1.70-1.60 (m, 4H); ^13^C NMR (100 MHz, CDCl_3_, Figure S32) *δ* 192.5, 152.6, 145.8, 143.4, 139.8, 138.2, 135.5, 132.5, 129.2 (2 carbons), 128.2 (2 carbons), 127.0, 108.7, 106.8, 63.5, 56.2, 53.1 (2 carbons), 37.2, 31.2 (2 carbons), 29.5; *m*/*z* calcd for C_23_H_25_NO_3_ 363.2; found 364.2 [M + H]^+^.

*2-((1-Benzylpiperidin-4-Yl)Methyl)-6-Hydroxy-5-Methoxy-2,3-Dihydro-1H-Inden-1-One* (**14**) (SGT332). To a solution of **13** (101 mg, 0.28 mmol) in degassed THF (2.5 mL) was added 10% Pd/C (wet support, Sigma 520829-10G, 10 mg). The reaction flask was then sealed with a rubber septum and freed of air. Thioanisole (14.2 × 10^−7^ mL, obtained using 5 μL of a stock solution comprising 14.2 μL of thioanisole in 50 mL of anhydrous THF) was added and the reaction mixture was stirred at rt overnight under H_2_ atmosphere. Upon completion, the reaction mixture was filtered through a bed of celite, and concentrated to afford the known compound **14** (96 mg, 94%) as a yellow solid: ^1^H NMR (400 MHz, CDCl_3_, Figure S33) *δ* 7.30-7.20 (m, 6H, aromatic), 6.82 (s, 1H, aromatic), 3.96 (s, 3H, OCH_3_), 3.49 (s, 2H, NCH_2_Ph), 3.20 (dd, **J*_1_* = 18.0 Hz, **J*_2_* = 7.6 Hz, 1H), 2.87 (m, 2H), 2.66 (dt, **J*_1_* = 13.6 Hz, **J*_2_* = 3.6 Hz, 2H), 1.98–1.82 (m, 3H), 1.72–1.63 (m, 2H), 1.48 (m, 1H), 1.39–1.24 (m, 3H); ^13^C NMR (100 MHz, CDCl_3_, Figure S34) *δ* 207.8, 152.9, 147.5, 145.8, 138.3, 130.0, 129.3 (2 carbons), 128.1 (2 carbons), 126.9, 108.0, 106.9, 63.4, 56.2, 53.7 (2 carbons), 45.3, 38.7, 34.4, 33.4, 32.9, 31.7; *m*/*z* calcd for C_23_H_27_NO_3_ 365.2; found 366.2 [M + H]^+^.

*(E)-2-((1-Benzylpiperidin-4-Yl)Methyl)-6-(2-(3-(3-(4-(Dimethylamino)Phenyl)Acryloyl)Phenoxy)Ethoxy)-5-Methoxy-2,3-Dihydro-1H-Inden-1-One* (**15a**) (SGT656). A solution of compound **14** (15 mg, 0.041 mmol), compound **4a** (19 mg, 0.049 mmol), and K_2_CO_3_ (17 mg, 0.12 mmol) in anhydrous DMF (5 mL) was heated at 65 °C overnight. The reaction mixture was then diluted with H_2_O, and extracted with EtOAc (3×). The combined organic layers were washed with H_2_O (3×) and brine (3×), dried over anhydrous MgSO_4_, filtered, and concentrated under reduced pressure. The crude product obtained was purified by column chromatography (SiO_2_ gel, pure CH_2_Cl_2_ to CH_2_Cl_2_:MeOH/19:1; R*_f_* 0.39 in CH_2_Cl_2_:MeOH/19:1) to yield compound **15a** (25 mg, 93%) as an orange oil: ^1^H NMR (400 MHz, CDCl_3_, Figure S35) *δ* 7.77 (d, *J* = 15.6 Hz, 1H, HC=CH-Ph), 7.59–7.56 (m, 2H, aromatic), 7.52 (d, *J* = 8.4 Hz, 2H, aromatic), 7.37 (t, *J* = 8.0 Hz, 1H, aromatic), 7.30–7.23 (m, 7H, aromatic), 7.13 (dd, *J*_1_ = 8.0 Hz, *J*_2_ = 2.4 Hz, 1H, aromatic), 6.83 (s, 1H, aromatic), 6.66 (d, *J* = 8.4 Hz, 2H, aromatic), 4.41 (m, 4H), 3.89 (s, 3H, OCH_3_), 3.52 (s, 2H, NCH_2_Ph), 3.20 (dd, *J*_1_ = 17.6 Hz, *J*_2_ = 8.0 Hz, 1H), 3.02 (s, 6H, N(CH_3_)_2_), 2.91 (m, 2H), 2.66 (dt, *J*_1_ = 14.0 Hz, *J*_2_ = 2.4 Hz, 2H), 1.99 (m, 2H), 1.89 (m, 1H), 1.65–1.55 (m, 2H), 1.55–1.40 (m, 1H), 1.40–1.25 (m, 3H); ^13^C NMR (100 MHz, CDCl_3_, Figure S36) *δ* 207.7, 190.1, 158.8, 155.9, 152.0, 149.2, 148.4 (2 carbons), 145.9, 140.4, 130.4 (2 carbons), 129.41, 129.37 (2 carbons), 129.1, 128.2 (2 carbons), 127.1, 122.6, 121.2, 119.2, 116.7, 113.8, 111.8 (2 carbons), 107.7, 106.3, 67.6, 66.5, 63.2, 56.1, 53.6, 53.5, 45.4, 40.1 (2 carbons), 38.6, 34.3, 33.4, 32.7, 31.5; *m/z* calcd for C_42_H_46_N_2_O_5_ 658.3; found 659.3 [M + H]^+^.

*(E)-2-((1-Benzylpiperidin-4-Yl)Methyl)-6-(4-(3-(3-(4-(Dimethylamino)Phenyl)Acryloyl)Phenoxy)Butoxy)-5-Methoxy-2,3-Dihydro-1H-Inden-1-One* (**15b**) (SGT681). A solution of compound **14** (30 mg, 0.082 mmol), compound **4b** (40 mg, 0.099 mmol), and K_2_CO_3_ (34 mg, 0.25 mmol) in anhydrous DMF (5 mL) was heated at 65 °C overnight. The reaction mixture was then diluted with H_2_O, and extracted with EtOAc (3×). The combined organic layers were washed with H_2_O (3×) and brine (3×), dried over anhydrous MgSO_4_, filtered, and concentrated under reduced pressure. The crude product obtained was purified by column chromatography (SiO_2_ gel, pure CH_2_Cl_2_ to CH_2_Cl_2_:MeOH/19:1; R*_f_* 0.39 in CH_2_Cl_2_:MeOH/19:1) to yield compound **15b** (48 mg, 86%) as an orange oil: ^1^H NMR (400 MHz, CDCl_3_, Figure S37) *δ* 7.77 (d, *J* = 15.2 Hz, 1H, HC=CH-Ph), 7.58–7.46 (m, 4H, aromatic), 7.38–7.22 (m, 7H, aromatic), 7.14 (s, 1H, aromatic), 7.06 (dd, *J*_1_ = 8.4 Hz, *J*_2_ = 2.0 Hz, 1H, aromatic), 6.80 (s, 1H, aromatic), 6.67 (d, *J* = 8.8 Hz, 2H, aromatic), 4.11 (m, 4H), 3.89 (s, 3H, OCH_3_), 3.57 (s, 2H, NCH_2_Ph), 3.19 (dd, *J*_1_ = 17.6 Hz, *J*_2_ = 8.4 Hz, 1H), 3.02 (s, 6H, N(CH_3_)_2_), 2.96 (m, 2H), 2.65 (dt, *J*_1_ = 14.0 Hz, *J*_2_ = 4.4 Hz, 2H), 2.05-1.98 (m, 6H), 1.89–1.84 (m, 1H), 1.70 (m, 2H), 1.60–1.22 (m, 4H); ^13^C NMR (100 MHz, CDCl_3_, Figure S38) *δ* 207.7, 190.3, 159.1, 155.8, 152.0, 148.7 (3 carbons), 145.8, 140.4, 130.4 (2 carbons), 129.8 (2 carbons), 129.3, 129.0, 128.4 (2 carbons), 127.7, 122.5, 120.7, 119.0, 116.8, 113.4, 111.8 (2 carbons), 107.4, 105.4, 68.6, 67.6, 62.7, 56.2, 53.3 (2 carbons), 45.1, 40.1 (2 carbons), 38.4, 33.7, 33.4, 31.8, 31.0, 26.1, 25.6; *m/z* calcd for C_44_H_50_N_2_O_5_ 686.4; found 687.3 [M + H]^+^.

*(E)-2-((1-Benzylpiperidin-4-Yl)Methyl)-6-((6-(3-(3-(4-(Dimethylamino)Phenyl)Acryloyl)Phenoxy)Hexyl)Oxy)-5-Methoxy-2,3-Dihydro-1H-Inden-1-One* (**15c**) (SGT687). A solution of compound **14** (30 mg, 0.082 mmol), compound **4c** (42 mg, 0.099 mmol), and K_2_CO_3_ (34 mg, 0.24 mmol) in anhydrous DMF (5 mL) was heated at 80 °C overnight. The reaction mixture was then diluted with H_2_O, and extracted with EtOAc (3×). The combined organic layers were washed with H_2_O (3×) and brine (3×), dried over anhydrous MgSO_4_, filtered, and concentrated under reduced pressure. The crude product obtained was purified by column chromatography (SiO_2_ gel, pure CH_2_Cl_2_ to CH_2_Cl_2_:MeOH/19:1; R*_f_* 0.39 in CH_2_Cl_2_:MeOH/19:1) to yield compound **15c** (61 mg, quantitative yield) as an orange solid: ^1^H NMR (400 MHz, CDCl_3_, Figure S39) *δ* 7.77 (d, *J* = 15.6 Hz, 1H, HC=CH-Ph), 7.58–7.48 (m, 4H, aromatic), 7.38–7.24 (m, 7H, aromatic), 7.13 (s, 1H, aromatic), 7.06 (d, *J* = 8.4 Hz, 1H, aromatic), 6.81 (s, 1H, aromatic), 6.67 (d, *J* = 8.4 Hz, 2H, aromatic), 4.02 (m, 4H), 3.91 (s, 3H, OCH_3_), 3.60 (s, 2H, NCH_2_Ph), 3.20 (dd, *J*_1_ = 17.2 Hz, *J*_2_ = 8.4 Hz, 1H), 3.03 (s, 6H, N(CH_3_)_2_), 2.97 (m, 2H), 2.65 (m, 2H), 2.06 (m, 2H), 1.92–1.78 (m, 5H), 1.71 (m, 2H), 1.54 (m, 4H), 1.45–1.23 (m, 4H); ^13^C NMR (100 MHz, CDCl_3_, Figure S40) *δ* 207.8, 190.4, 159.2, 155.8, 152.0, 148.8, 148.6 (2 carbons), 145.8, 140.4, 130.4 (2 carbons), 129.6, 129.3 (2 carbons), 129.1, 128.3 (2 carbons), 127.5, 122.6, 120.6, 119.0, 116.9, 113.4, 111.8 (2 carbons), 107.4, 105.4, 68.9, 67.9, 62.9, 56.2, 53.4 (2 carbons), 45.2, 40.1 (2 carbons), 38.5, 34.0, 33.4, 32.2, 31.2, 29.1, 28.8, 25.82, 25.75; *m/z* calcd for C_46_H_54_N_2_O_5_ 714.4; found 715.2 [M + H]^+^.

*(E)-2-((1-Benzylpiperidin-4-Yl)Methyl)-6-((8-(3-(3-(4-(Dimethylamino)Phenyl)Acryloyl)Phenoxy)Octyl)Oxy)-5-Methoxy-2,3-Dihydro-1H-Inden-1-One* (**15d**) (SGT688). A solution of compound **14** (30 mg, 0.082 mmol), compound **4d** (45 mg, 0.099 mmol), and K_2_CO_3_ (34 mg, 0.24 mmol) in anhydrous DMF (5 mL) was heated at 80 °C overnight. The reaction mixture was then diluted with H_2_O, and extracted with EtOAc (3×). The combined organic layers were washed with H_2_O (3×) and brine (3×), dried over anhydrous MgSO_4_, filtered, and concentrated under reduced pressure. The crude product obtained was purified by column chromatography (SiO_2_ gel, pure CH_2_Cl_2_ to CH_2_Cl_2_:MeOH/19:1; R*_f_* 0.39 in CH_2_Cl_2_:MeOH/19:1) to yield compound **15d** (58 mg, 95%) as an orange solid: ^1^H NMR (400 MHz, CDCl_3_, Figure S41) *δ* 7.77 (d, *J* = 15.2 Hz, 1H, HC=CH-Ph), 7.56–7.48 (m, 4H, aromatic), 7.38–7.24 (m, 7H, aromatic), 7.13 (s, 1H, aromatic), 7.06 (d, *J* = 8.4 Hz, 1H, aromatic), 6.82 (s, 1H, aromatic), 6.67 (d, *J* = 8.4 Hz, 2H, aromatic), 4.01 (t, *J* = 6.4 Hz, 4H), 3.92 (s, 3H, OCH_3_), 3.57 (s, 2H, NCH_2_Ph), 3.20 (dd, *J*_1_ = 17.2 Hz, *J*_2_ = 8.0 Hz, 1H), 3.03 (s, 6H, N(CH_3_)_2_), 2.95 (m, 2H), 2.65 (m, 2H), 2.03 (m, 2H), 1.92–1.65 (m, 7H), 1.45 (m, 5H), 1.42–1.23 (m, 7H); ^13^C NMR (100 MHz, CDCl_3_, Figure S42) *δ* 207.8, 190.4, 159.3, 155.7, 152.0, 148.8, 148.5 (2 carbons), 145.8, 140.4, 130.4 (2 carbons), 129.5, 129.3 (2 carbons), 129.1, 128.2 (2 carbons), 127.3, 122.6, 120.6, 119.0, 116.9, 113.4, 111.8 (2 carbons), 107.4, 105.4, 69.0, 68.1, 63.1, 56.2, 53.5 (2 carbons), 45.3, 40.1 (2 carbons), 38.6, 34.2, 33.4, 32.4, 31.4, 29.3 (2 carbons), 29.2, 28.9, 25.95, 25.85; *m/z* calcd for C_48_H_58_N_2_O_5_ 742.4; found 743.4 [M + H]^+^.

*(E)-2-((1-Benzylpiperidin-4-Yl)Methyl)-6-((10-(3-(3-(4-(Dimethylamino)Phenyl)Acryloyl)Phenoxy)Decyl)Oxy)-5-Methoxy-2,3-Dihydro-1H-Inden-1-One* (**15e**) (SGT689). A solution of compound **14** (30 mg, 0.082 mmol), compound **4e** (48 mg, 0.099 mmol), and K_2_CO_3_ (34 mg, 0.24 mmol) in anhydrous DMF (5 mL) was heated at 80 °C overnight. The reaction mixture was then diluted with H_2_O, and extracted with EtOAc (3×). The combined organic layers were washed with H_2_O (3×) and brine (3×), dried over anhydrous MgSO_4_, filtered, and concentrated under reduced pressure. The crude product obtained was purified by column chromatography (SiO_2_ gel, pure CH_2_Cl_2_ to CH_2_Cl_2_:MeOH/19:1; R*_f_* 0.39 in CH_2_Cl_2_:MeOH/19:1) to yield compound **15e** (42 mg, 67%) as a yellow solid: ^1^H NMR (400 MHz, CDCl_3_, Figure S43) *δ* 7.77 (d, *J* = 15.6 Hz, 1H, HC=CH-Ph), 7.56–7.48 (m, 4H, aromatic), 7.38–7.24 (m, 7H, aromatic), 7.13 (s, 1H, aromatic), 7.07 (d, *J* = 8.4 Hz, 1H, aromatic), 6.81 (s, 1H, aromatic), 6.67 (d, *J* = 8.4 Hz, 2H, aromatic), 4.01 (t, *J* = 6.8 Hz, 4H, 2×OCH_2_CH_2_), 3.91 (s, 3H, OCH_3_), 3.54 (s, 2H, NCH_2_Ph), 3.20 (dd, *J*_1_ = 17.2 Hz, *J*_2_ = 8.0 Hz, 1H), 3.03 (s, 6H, N(CH_3_)_2_), 2.92 (m, 2H), 2.65 (m, 2H), 2.01 (m, 2H), 1.92-1.65 (m, 7H), 1.43 (m, 5H), 1.31 (m, 11H); ^13^C NMR (100 MHz, CDCl_3_, Figure S44) *δ* 207.8, 190.4, 159.3, 155.7, 152.0, 148.8, 148.5 (2 carbons), 145.8, 140.4, 130.4 (2 carbons), 129.4, 129.3 (2 carbons), 129.2, 128.2 (2 carbons), 127.2, 122.6, 120.6, 119.0, 116.9, 113.4, 111.8 (2 carbons), 107.4, 105.4, 69.0, 68.2, 63.2, 56.2, 53.6 (2 carbons), 45.3, 40.1 (2 carbons), 38.6, 34.2, 33.3, 32.6, 31.5, 29.7 (2 carbons), 29.4, 29.3, 29.2, 28.9, 26.0, 25.9; *m/z* calcd for C_50_H_62_N_2_O_5_ 770.5; found 771.3 [M + H]^+^.

*(E)-2-((1-Benzylpiperidin-4-Yl)Methyl)-6-((12-(3-(3-(4-(Dimethylamino)Phenyl)Acryloyl)Phenoxy)Dodecyl)Oxy)-5-Methoxy-2,3-Dihydro-1H-Inden-1-One* (**15f**) (SGT679). A solution of compound **14** (30 mg, 0.082 mmol), compound **4f** (51 mg, 0.099 mmol), and K_2_CO_3_ (34 mg, 0.25 mmol) in anhydrous DMF (5 mL) was heated at 80 °C overnight. The reaction mixture was then diluted with H_2_O, and extracted with EtOAc (3×). The combined organic layers were washed with H_2_O (3×) and brine (3×), dried over anhydrous MgSO_4_, filtered, and concentrated under reduced pressure. The crude product obtained was purified by column chromatography (SiO_2_ gel, pure CH_2_Cl_2_ to CH_2_Cl_2_:MeOH/19:1; R*_f_* 0.39 in CH_2_Cl_2_:MeOH/19:1) to yield compound **15f** (32 mg, 48%) as an orange oil: ^1^H NMR (400 MHz, CDCl_3_, Figure S45) *δ* 7.77 (d, *J* = 15.2 Hz, 1H, HC=CH-Ph), 7.56–7.50 (m, 4H, aromatic), 7.35 (t, *J* = 8.0 Hz, 1H, aromatic), 7.33–7.28 (m, 6H, aromatic), 7.12 (s, 1H, aromatic), 7.06 (dd, *J*_1_ = 8.0 Hz, *J*_2_ = 2.0 Hz, 1H, aromatic), 6.81 (s, 1H, aromatic), 6.67 (d, *J* = 8.8 Hz, 2H, aromatic), 4.00 (m, 4H), 3.91 (s, 3H, OCH_3_), 3.57 (s, 2H, NCH_2_Ph), 3.19 (dd, *J*_1_ = 17.6 Hz, *J*_2_ = 8.0 Hz, 1H), 3.02 (s, 6H, N(CH_3_)_2_), 2.95 (m, 2H), 2.65 (dt, *J*_1_ = 14.0 Hz, *J*_2_ = 4.4 Hz, 2H), 2.03 (m, 2H), 1.90–1.75 (m, 5H), 1.73–1.60 (m, 2H), 1.50–1.40 (m, 6H), 1.40–1.25 (m, 14H); ^13^C NMR (100 MHz, CDCl_3_, Figure S46) *δ* 207.8, 190.4, 159.3, 155.7, 152.0, 148.8, 148.5 (2 carbons), 145.8, 140.4, 130.4 (2 carbons), 129.5, 129.3 (2 carbons), 129.1, 128.3 (2 carbons), 127.3, 122.6, 120.6, 119.0, 116.9, 113.4, 111.8 (2 carbons), 107.4, 105.4, 69.1, 68.2, 62.9, 56.2, 53.4 (2 carbons), 45.3, 40.1 (2 carbons), 38.6, 34.1, 33.3, 32.3, 31.3, 29.5 (3 carbons), 29.5, 29.4, 29.3, 29.2, 28.9, 26.0, 25.9; *m/z* calcd for C_52_H_66_N_2_O_5_ 798.5; found 799.3 [M + H]^+^.

*(E)-2-((1-Benzylpiperidin-4-Yl)Methyl)-6-(2-(4-(3-(4-(Dimethylamino)Phenyl)Acryloyl)Phenoxy)Ethoxy)-5-Methoxy-2,3-Dihydro-1H-Inden-1-One* (**16a**) (SGT683). A solution of compound **14** (20 mg, 0.055 mmol), compound **7a** (25 mg, 0.066 mmol), and K_2_CO_3_ (23 mg, 0.16 mmol) in anhydrous DMF (5 mL) was heated at 80 °C overnight. The reaction mixture was then diluted with H_2_O, and extracted with EtOAc (3×). The combined organic layers were washed with H_2_O (3×) and brine (3×), dried over anhydrous MgSO_4_, filtered, and concentrated under reduced pressure. The crude product obtained was purified by column chromatography (SiO_2_ gel, pure CH_2_Cl_2_ to CH_2_Cl_2_:MeOH/19:1; R*_f_* 0.39 in CH_2_Cl_2_:MeOH/19:1) to yield compound **16a** (48 mg, quantitative yield) as a yellow solid: ^1^H NMR (400 MHz, CDCl_3_, Figure S47) *δ* 8.00 (d, *J* = 8.8 Hz, 2H, aromatic), 7.76 (d, *J* = 15.6 Hz, 1H, HC=CH-Ph), 7.53 (d, *J* = 8.8 Hz, 2H, aromatic), 7.33 (d, *J* = 15.6 Hz, 1H, HC=CH-Ph), 7.32–7.24 (m, 5H, aromatic), 7.23 (s, 1H, aromatic), 7.00 (d, *J* = 8.8 Hz, 2H, aromatic), 6.84 (s, 1H, aromatic), 6.67 (d, *J* = 8.8 Hz, 2H, aromatic), 4.41 (m, 4H), 3.90 (s, 3H, OCH_3_), 3.55 (s, 2H, NCH_2_Ph), 3.21 (dd, *J*_1_ = 17.6 Hz, *J*_2_ = 8.0 Hz, 1H), 3.02 (s, 6H, N(CH_3_)_2_), 2.92 (m, 2H), 2.67 (dt, *J*_1_ = 14.4 Hz, *J*_2_ = 3.2 Hz, 2H), 2.02 (m, 2H), 1.89 (m, 1H), 1.70 (m, 2H), 1.50 (m, 1H), 1.40–1.25 (m, 3H); ^13^C NMR (100 MHz, CDCl_3_, Figure S48) *δ* 207.6, 188.9, 161.9, 155.9, 151.9, 149.3, 148.4 (2 carbons), 145.0, 132.2, 130.5 (2 carbons), 130.3 (2 carbons), 129.4 (2 carbons) 129.1, 128.2 (2 carbons), 127.2, 122.8, 116.6, 114.3 (2 carbons), 111.8 (2 carbons), 107.8, 106.3, 67.4, 66.4, 63.1, 56.2, 53.5 (2 carbons), 45.3, 40.1 (2 carbons), 38.6, 34.2, 33.4, 32.5, 31.5; *m/z* calcd for C_42_H_46_N_2_O_5_ 658.3; found 659.3 [M + H]^+^.

*(E)-2-((1-Benzylpiperidin-4-Yl)Methyl)-6-(4-(4-(3-(4-(Dimethylamino)Phenyl)Acryloyl)Phenoxy)Butoxy)-5-Methoxy-2,3-Dihydro-1H-Inden-1-One* (**16b**) (SGT682). A solution of compound **14** (20 mg, 0.055 mmol), compound **7b** (27 mg, 0.066 mmol), and K_2_CO_3_ (23 mg, 0.16 mmol) in anhydrous DMF (5 mL) was heated at 65 °C overnight. The reaction mixture was then diluted with H_2_O, and extracted with EtOAc (3×). The combined organic layers were washed with H_2_O (3×) and brine (3×), dried over anhydrous MgSO_4_, filtered, and concentrated under reduced pressure. The crude product obtained was purified by column chromatography (SiO_2_ gel, pure CH_2_Cl_2_ to CH_2_Cl_2_:MeOH/19:1; R*_f_* 0.39 in CH_2_Cl_2_:MeOH/19:1) to yield compound **16b** (14 mg, 37%) as an orange oil: ^1^H NMR (400 MHz, CDCl_3_, Figure S49) *δ* 7.98 (d, *J* = 8.8 Hz, 2H, aromatic), 7.76 (d, *J* = 15.2 Hz, 1H, HC=CH-Ph), 7.53 (d, *J* = 8.8 Hz, 2H, aromatic), 7.33 (d, *J* = 15.2 Hz, 1H, HC=CH-Ph), 7.33–7.26 (m, 5H, aromatic), 7.13 (s, 1H, aromatic), 6.93 (d, *J* = 8.8 Hz, 2H, aromatic), 6.81 (s, 1H, aromatic), 6.67 (d, *J* = 8.8 Hz, 2H, aromatic), 4.41 (q, *J* = 5.6 Hz, 4H), 3.89 (s, 3H, OCH_3_), 3.62 (s, 2H, NCH_2_Ph), 3.21 (dd, *J*_1_ = 17.6 Hz, *J*_2_ = 8.4 Hz, 1H), 3.02 (s, 6H, N(CH_3_)_2_), 2.92 (m, 2H), 2.67 (dt, *J*_1_ = 14.0 Hz, *J*_2_ = 4.0 Hz, 2H), 2.10 (m, 2H), 2.02 (m, 4H), 1.88 (m, 1H), 1.72 (m, 2H), 1.60–1.20 (m, 4H); ^13^C NMR (100 MHz, CDCl_3_, Figure S50) *δ* 207.7, 188.9, 162.4, 155.8, 151.9, 148.7 (3 carbons), 144.9, 132.1, 131.7, 131.0, 130.5 (2 carbons), 130.2 (2 carbons), 129.7, 129.1, 128.3 (2 carbons), 122.8, 116.6, 114.1 (2 carbons), 111.8 (2 carbons), 107.5, 105.5, 68.6, 67.6, 62.7, 56.1, 53.3, 45.2, 40.1 (2 carbons), 38.5, 33.9, 33.4, 31.9, 31.1, 29.7, 26.0, 25.5; *m/z* calcd for C_44_H_50_N_2_O_5_ 686.4; found 687.4 [M + H]^+^.

*(E)-2-((1-Benzylpiperidin-4-Yl)Methyl)-6-((6-(4-(3-(4-(Dimethylamino)Phenyl)Acryloyl)Phenoxy)Hexyl)Oxy)-5-Methoxy-2,3-Dihydro-1H-Inden-1-One* (**16c**) (SGT694). A solution of compound **14** (25 mg, 0.068 mmol), compound **7c** (35 mg, 0.082 mmol), and K_2_CO_3_ (28 mg, 0.21 mmol) in anhydrous DMF (5 mL) was heated at 80 °C overnight. The reaction mixture was then diluted with H_2_O, and extracted with EtOAc (3×). The combined organic layers were washed with H_2_O (3×) and brine (3×), dried over anhydrous MgSO_4_, filtered, and concentrated under reduced pressure. The crude product obtained was purified by column chromatography (SiO_2_ gel, pure CH_2_Cl_2_ to CH_2_Cl_2_:MeOH/19:1; R*_f_* 0.39 in CH_2_Cl_2_:MeOH/19:1) to yield compound **16c** (48 mg, 98%) as a yellow solid: ^1^H NMR (400 MHz, CDCl_3_, Figure S51) *δ* 7.99 (d, *J* = 8.8 Hz, 2H, aromatic), 7.76 (d, *J* = 15.2 Hz, 1H, HC=CH-Ph), 7.53 (d, *J* = 8.4 Hz, 2H, aromatic), 7.36–7.24 (m, 6H, aromatic), 7.14 (s, 1H, aromatic), 6.93 (d, *J* = 8.4 Hz, 2H, aromatic), 6.82 (s, 1H, aromatic), 6.68 (d, *J* = 8.8 Hz, 2H, aromatic), 4.032 (t, *J* = 6.0 Hz, 2H), 4.025 (t, *J* = 6.0 Hz, 2H), 3.91 (s, 3H, OCH_3_), 3.54 (s, 2H, NCH_2_Ph), 3.20 (dd, *J*_1_ = 17.6 Hz, *J*_2_ = 8.4 Hz, 1H), 3.02 (s, 6H, N(CH_3_)_2_), 2.92 (m, 2H), 2.65 (m, 2H), 2.00 (m, 2H), 1.90–1.80 (m, 9H), 1.60–1.20 (m, 6H); ^13^C NMR (100 MHz, CDCl_3_, Figure S52) *δ* 207.7, 188.8, 162.5, 155.7, 151.8, 148.8, 148.5 (3 carbons), 144.8, 131.6, 130.5, 130.2, 129.5, 129.1 (2 carbons), 128.2 (2 carbons), 127.3, 122.8 (2 carbons), 116.6, 114.1, 111.8 (2 carbons), 107.4, 105.4, 68.8, 67.9, 63.0, 56.2, 56.1, 53.4, 45.3, 40.1, 38.5 (2 carbons), 33.9, 33.3, 29.0, 28.8, 25.71, 25.66, 25.58, 24.9; *m/z* calcd for C_46_H_54_N_2_O_5_ 714.4; found 715.3 [M + H]^+^.

*(E)-2-((1-Benzylpiperidin-4-Yl)Methyl)-6-((8-(4-(3-(4-(Dimethylamino)Phenyl)Acryloyl)Phenoxy)Octyl)Oxy)-5-Methoxy-2,3-Dihydro-1H-Inden-1-One* (**16d**) (SGT695). A solution of compound **14** (25 mg, 0.068 mmol), compound **7d** (38 mg, 0.082 mmol), and K_2_CO_3_ (28 mg, 0.21 mmol) in anhydrous DMF (5 mL) was heated at 80 °C overnight. The reaction mixture was then diluted with H_2_O, and extracted with EtOAc (3×). The combined organic layers were washed with H_2_O (3×) and brine (3×), dried over anhydrous MgSO_4_, filtered, and concentrated under reduced pressure. The crude product obtained was purified by column chromatography (SiO_2_ gel, pure CH_2_Cl_2_ to CH_2_Cl_2_:MeOH/19:1; R*_f_* 0.40 in CH_2_Cl_2_:MeOH/19:1) to yield compound **16d** (51 mg, quantitative yield) as a yellow solid: ^1^H NMR (400 MHz, CDCl_3_, Figure S53) *δ* 7.99 (d, *J* = 9.2 Hz, 2H, aromatic), 7.76 (d, *J* = 15.2 Hz, 1H, HC=CH-Ph), 7.53 (d, *J* = 8.8 Hz, 2H, aromatic), 7.36–7.24 (m, 6H, aromatic), 7.13 (s, 1H, aromatic), 6.93 (d, *J* = 8.8 Hz, 2H, aromatic), 6.82 (s, 1H, aromatic), 6.68 (d, *J* = 8.4 Hz, 2H, aromatic), 4.01 (t, *J* = 6.4 Hz, 4H), 3.92 (s, 3H, OCH_3_), 3.54 (s, 2H, NCH_2_Ph), 3.20 (dd, *J*_1_ = 17.6 Hz, *J*_2_ = 8.4 Hz, 1H), 3.02 (s, 6H, N(CH_3_)_2_), 2.92 (m, 2H), 2.65 (m, 2H), 2.00 (m, 2H), 1.90–1.76 (m, 5H), 1.76–1.65 (m, 2H), 1.50–1.20 (m, 12H); ^13^C NMR (100 MHz, CDCl_3_, Figure S54) *δ* 207.8, 188.9, 162.5, 155.7, 151.8, 148.8, 148.5 (3 carbons), 144.8, 131.6, 130.5 (2 carbons), 130.2 (2 carbons), 129.4, 129.2, 128.2 (2 carbons), 127.2, 122.9, 116.7, 114.1 (2 carbons), 111.8 (2 carbons), 107.4, 105.4, 69.0, 68.1, 63.2, 56.2, 53.6 (2 carbons), 45.4, 40.1 (2 carbons), 38.6, 34.2, 33.3, 31.5, 29.7, 29.2 (2 carbons), 29.1, 28.8, 25.9, 25.8; *m/z* calcd for C_48_H_58_N_2_O_5_ 742.4; found 743.3 [M + H]^+^.

*(E)-2-((1-Benzylpiperidin-4-Yl)Methyl)-6-((10-(4-(3-(4-(Dimethylamino)Phenyl)Acryloyl)Phenoxy)Decyl)Oxy)-5-Methoxy-2,3-Dihydro-1H-Inden-1-One* (**16e**) (SGT696). A solution of compound **16** (20 mg, 0.055 mmol), compound **7e** (32 mg, 0.066 mmol), and K_2_CO_3_ (23 mg, 0.16 mmol) in anhydrous DMF (5 mL) was heated at 80 °C overnight. The reaction mixture was then diluted with H_2_O, and extracted with EtOAc (3×). The combined organic layers were washed with H_2_O (3×) and brine (3×), dried over anhydrous MgSO_4_, filtered, and concentrated under reduced pressure. The crude product obtained was purified by column chromatography (SiO_2_ gel, pure CH_2_Cl_2_ to CH_2_Cl_2_:MeOH/19:1; R*_f_* 0.40 in CH_2_Cl_2_:MeOH/19:1) to yield compound **16e** (40 mg, 95%) as a yellow solid: ^1^H NMR (400 MHz, CDCl_3_, Figure S55) *δ* 7.99 (d, *J* = 8.8 Hz, 2H, aromatic), 7.76 (d, *J* = 15.2 Hz, 1H, HC=CH-Ph), 7.53 (d, *J* = 8.8 Hz, 2H, aromatic), 7.36–7.24 (m, 6H, aromatic), 7.13 (s, 1H, aromatic), 6.93 (d, *J* = 8.8 Hz, 2H, aromatic), 6.82 (s, 1H, aromatic), 6.68 (d, *J* = 8.0 Hz, 2H, aromatic), 4.01 (t, *J* = 6.8 Hz, 4H), 3.92 (s, 3H, OCH_3_), 3.55 (s, 2H, NCH_2_Ph), 3.20 (dd, *J*_1_ = 17.6 Hz, *J*_2_ = 8.4 Hz, 1H), 3.02 (s, 6H, N(CH_3_)_2_), 2.94 (m, 2H), 2.65 (m, 2H), 2.02 (m, 2H), 1.92–1.60 (m, 7H), 1.50–1.20 (m, 16H); ^13^C NMR (100 MHz, CDCl_3_, Figure S56) *δ* 207.8, 188.9, 162.6, 155.8, 151.8, 148.8, 148.5 (3 carbons), 144.8, 131.6, 130.5 (2 carbons), 130.2 (2 carbons), 129.4, 129.2, 128.2 (2 carbons), 127.2, 122.9, 116.7, 114.1 (2 carbons), 111.8 (2 carbons), 107.4, 105.4, 69.0, 68.2, 63.1, 56.2, 53.6 (2 carbons), 45.3, 40.1 (2 carbons), 38.6, 34.2, 33.4, 31.5, 29.7, 29.42, 29.40, 29.30, 29.28, 29.1, 28.9, 26.0, 25.9; *m/z* calcd for C_50_H_62_N_2_O_5_ 770.5; found 771.4 [M + H]^+^.

*(E)-2-((1-Benzylpiperidin-4-Yl)Methyl)-6-((12-(4-(3-(4-(Dimethylamino)Phenyl)Acryloyl)Phenoxy)Dodecyl)Oxy)-5-Methoxy-2,3-Dihydro-1H-Inden-1-One* (**16f**) (SGT680). A solution of compound **14** (25 mg, 0.068 mmol), compound **7f** (42 mg, 0.082 mmol), and K_2_CO_3_ (28 mg, 0.21 mmol) in anhydrous DMF (5 mL) was heated at 80 °C overnight. The reaction mixture was then diluted with H_2_O, and extracted with EtOAc (3×). The combined organic layers were washed with H_2_O (3×) and brine (3×), dried over anhydrous MgSO_4_, filtered, and concentrated under reduced pressure. The crude product obtained was purified by column chromatography (SiO_2_ gel, pure CH_2_Cl_2_ to CH_2_Cl_2_:MeOH/19:1; R*_f_* 0.39 in CH_2_Cl_2_:MeOH/19:1) to yield compound **16f** (56 mg, quantitative yield) as an orange solid: ^1^H NMR (400 MHz, CDCl_3_, Figure S57) *δ* 7.99 (d, *J* = 8.8 Hz, 2H, aromatic), 7.76 (d, *J* = 15.6 Hz, 1H, HC=CH-Ph), 7.53 (d, *J* = 8.8 Hz, 2H, aromatic), 7.38–7.26 (m, 6H, aromatic), 7.12 (s, 1H, aromatic), 6.93 (d, *J* = 8.8 Hz, 2H, aromatic), 6.81 (s, 1H, aromatic), 6.67 (d, *J* = 8.4 Hz, 2H, aromatic), 4.01 (t, *J* = 6.4 Hz, 2H), 4.00 (t, *J* = 6.4 Hz, 2H), 3.91 (s, 3H, OCH_3_), 3.67 (s, 2H, NCH_2_Ph), 3.21 (dd, *J*_1_ = 17.6 Hz, *J*_2_ = 8.4 Hz, 1H), 3.03 (m, 2H), 3.02 (s, 6H, N(CH_3_)_2_), 2.64 (m, 2H), 2.16 (m, 2H), 2.00 (m, 1H), 1.92–1.70 (m, 7H), 1.50–1.20 (m, 19H); ^13^C NMR (100 MHz, CDCl_3_, Figure S58) *δ* 207.6, 188.9, 162.6, 155.8, 151.8, 148.9, 148.5 (3 carbons), 144.8, 131.6, 130.5 (2 carbons), 130.2 (2 carbons), 129.8, 129.1, 128.4 (2 carbons), 127.8, 122.9, 116.7, 114.1 (2 carbons), 111.8 (2 carbons), 107.4, 105.4, 69.1, 68.2, 62.5, 56.2, 53.2 (2 carbons), 45.1, 40.1 (2 carbons), 38.4, 33.6, 33.4, 31.0, 29.7, 29.50 (3 carbons), 29.47, 29.33, 29.30, 29.1, 28.9, 26.0, 25.9; *m/z* calcd for C_52_H_66_N_2_O_5_ 798.5; found 799.3 [M + H]^+^.

*(E)-3-(4-(Dimethylamino)Phenyl)-1-(3-(2-Hydroxyethoxy)Phenyl)Prop-2-En-1-One* (**17**) (SGT863). A solution of compound (50 mg, 0.19 mmol) and K_2_CO_3_ (52 mg, 0.37 mmol) in anhydrous MeCN (5 mL) was treated with 2-bromoethanol (30 μL, 0.37 mmol) and the resulting mixture was refluxed overnight. The solvent was then removed and the obtained crude product was purified by column chromatography (SiO_2_ gel, pure Hexanes to Hexanes:EtOAc/1:1; R*_f_* 0.32 in Hexanes:EtOAc/1:1) to yield compound **17** (14 mg, 24%) as a red solid: ^1^H NMR (400 MHz, CDCl_3_, Figure S59) *δ* 7.78 (d, *J* = 15.2 Hz, 1H, HC=CH-Ph), 7.60–7.58 (m, 1H, aromatic), 7.55–7.51 (m, 3H, aromatic), 7.38 (t, *J* = 8.4 Hz, 1H, aromatic), 7.29 (d, *J* = 16.0 Hz, 1H, HC=CH-Ph), 7.10 (ddd, **J*_1_* = 8.4 Hz, **J*_2_* = 2.8 Hz, *J_3_* = 0.8 Hz, 1H, aromatic), 6.68 (d, *J* = 8.8 Hz, 2H, aromatic), 4.15 (t, *J* = 4.4 Hz, 2H, HOCH_2_CH_2_OPh), 3.98 (q, *J* = 4.0 Hz, 2H, HOCH_2_CH_2_OPh), 3.03 (s, 6H, N(CH_3_)_2_); ^13^C NMR (125 MHz, CDCl_3_, Figure S60) *δ* 189.3, 157.9, 145.1, 139.6, 129.6 (3 carbons), 128.6, 120.3, 118.2 (2 carbons), 116.0, 112.7 (2 carbons), 111.1, 68.5, 60.5, 39.3 (2 carbons); *m*/*z* calcd for C_19_H_21_NO_3_ 311.2; found 312.1 [M + H]^+^.

*2-(2-Bromoethoxy)Tetrahydro-2H-Pyran* (**18**) (SGT864). A mixture of 2-bromoethanol (353 mg, 2.8 mmol), 3,4-dihydro-2*H*-pyran (0.31 mL, 3.4 mmol), and *p*-TsOH monohydrate (11 mg, 0.06 mmol) in anhydrous CH_2_Cl_2_ (10 mL) was stirred at rt overnight. The reaction mixture was washed with NaHCO_3_, H_2_O, and brine, dried over anhydrous MgSO_4_, filtered, and concentrated under reduced pressure. The crude product obtained was purified by column chromatography (SiO_2_ gel, Hexanes:EtOAc/19:1; R*_f_* 0.52 in Hexanes:EtOAc/9:1) to yield the known compound **18** [72] (362 mg, 62%) as a colorless oil: ^1^H NMR (400 MHz, CDCl_3_, Figure S61, which matches the lit. [72]) *δ* 4.66 (t, *J* = 3.6 Hz, 1H), 3.99 (dt, **J*_1_* = 11.2 Hz, **J*_2_* = 6.4 Hz, 1H), 3.87 (ddd, **J*_1_* = 11.6 Hz, **J*_2_* = 8.0 Hz, *J_3_* = 2.8 Hz, 1H), 3.75 (dt, **J*_1_* = 11.2 Hz, **J*_2_* = 6.8 Hz, 1H), 3.54–3.44 (m, 3H), 1.88–1.77 (m, 1H), 1.76–1.66 (m, 1H), 1.65–1.48 (m, 4H).

*2-((1-Benzylpiperidin-4-Yl)Methyl)-5-Methoxy-6-(2-((Tetrahydro-2H-Pyran-2-Yl)Oxy)Ethoxy)-2,3-Dihydro-1H-Inden-1-One* (**19**) (SGT1729). A solution of compound **14** (50 mg, 0.14 mmol) and K_2_CO_3_ (39 mg, 0.27 mmol) in anhydrous DMF (5 mL) was treated with a solution of compound **18** (57 mg, 0.27 mmol) in DMF (1 mL), and the resulting mixture was stirred at rt overnight. The reaction mixture was quenched with H_2_O and extracted with EtOAc (3×). The combined organic layers were washed with H_2_O (3×), and brine (3×), dried over anhydrous MgSO_4_, filtered, and concentrated under reduced pressure. The crude product obtained was purified by column chromatography (SiO_2_ gel, pure CH_2_Cl_2_ to CH_2_Cl_2_:MeOH/19:1; R*_f_* 0.21 in CH_2_Cl_2_:MeOH/19:1) to yield compound **19** (63 mg, 93%) as a yellow oil: ^1^H NMR (400 MHz, CDCl_3_, Figure S62) *δ* 7.35–7.22 (m, 5H, aromatic), 7.21 (s, 1H, aromatic), 6.82 (s, 1H, aromatic), 4.70 (t, *J* = 3.6 Hz, 2H), 4.21 (t, *J* = 4.8 Hz, 2H), 4.05 (m, 1H), 3.91 (s, 3H, OCH_3_), 3.87–3.82 (m, 2H), 3.49 (m, 2H, NCH_2_Ph), 3.20 (dd, **J*_1_* = 17.6 Hz, **J*_2_* = 8.4 Hz, 1H), 2.88 (m, 2H), 2.66 (dt, **J*_1_* = 14.4 Hz, **J*_2_* = 3.6 Hz, 2H), 2.00–1.76 (m, 4H), 1.75–1.48 (m, 8H), 1.38–1.23 (m, 3H); ^13^C NMR (100 MHz, CDCl_3_, Figure S63) 207.6, 155.9, 148.8, 148.7, 129.5 (2 carbons), 129.1 (2 carbons), 128.2 (2 carbons), 127.3, 107.6, 106.3, 98.9, 68.5, 65.42, 65.41, 63.1, 62.1, 56.12, 56.10, 53.5, 45.3, 38.6, 34.1, 33.4, 31.4, 30.4, 25.4, 19.2; *m*/*z* calcd for C_30_H_39_NO_5_ 493.3; found 494.2 [M + H]^+^.

*2-((1-Benzylpiperidin-4-Yl)Methyl)-6-(2-Hydroxyethoxy)-5-Methoxy-2,3-Dihydro-1H-Inden-1-One* (**20**) (SGT1732). A solution of compound **19** (50 mg, 0.10 mmol) and *p*-TsOH monohydrate (19 mg, 0.10 mmol) in MeOH (5 mL) was stirred at rt overnight. The solvents were removed, and the crude material obtained was dissolved in EtOAc, washed with NaHCO_3_, H_2_O, and brine, dried over anhydrous MgSO_4_, filtered, and concentrated under reduced pressure. The crude product obtained was purified by column chromatography (SiO_2_ gel, pure CH_2_Cl_2_ to CH_2_Cl_2_:MeOH/19:1; R*_f_* 0.16 in CH_2_Cl_2_:MeOH/19:1) to yield compound **20** (18 mg, 44%) as a colorless oil: ^1^H NMR (400 MHz, CDCl_3_, Figure S64) *δ* 7.35–7.22 (m, 5H, aromatic), 7.18 (s, 1H, aromatic), 6.84 (s, 1H, aromatic), 4.12 (t, *J* = 4.4 Hz, 2H), 3.96 (t, *J* = 4.4 Hz, 2H), 3.92 (s, 3H, OCH_3_), 3.55 (br s, 2H, NCH_2_Ph), 3.22 (dd, **J*_1_* = 17.6 Hz, **J*_2_* = 8.0 Hz, 1H), 2.93 (m, 2H), 2.67 (dt, **J*_1_* = 14.0 Hz, **J*_2_* = 3.2 Hz, 2H), 2.10–1.94 (m, 2H), 1.93–1.84 (m, 1H), 1.80–1.30 (m, 6H); ^13^C NMR (100 MHz, CDCl_3_, Figure S65) 207.5, 155.9, 149.2, 148.4, 129.6 (2 carbons), 129.2 (2 carbons), 128.3 (2 carbons), 127.5, 107.6, 106.7, 70.7, 62.9, 61.0, 56.1, 53.41, 53.35, 45.2, 38.5, 33.9, 33.4 (2 carbons), 31.2; *m*/*z* calcd for C_25_H_31_NO_4_ 409.23; found 410.2 [M + H]^+^.

### 3.3. In Vitro Inhibition of EeAChE and EfBChE

The ChE inhibition assays were performed as previously described [29]. The 1,3- and 1,4-chalcone-donepezil hybrids (0.1 nM to 100 μM) were dissolved in sodium phosphate buffer ((100 μL), 0.1 M, pH 8.0). They were diluted 5-fold and either *Ee*AChE or *Ef*BChE was added to the solution of the inhibitors (50 μL, containing 0.08 U/mL ChE (final concentration for both *Ee*AChE and *Ef*BChE). The enzymes and inhibitors were incubated for 10 min followed by the addition of DTNB (50 μL, 0.25 mM final concentration) and acylthiocholine (acetylthiocholine for *Ee*AChE and butyrylthiocholine for *Ef*BChE). The reactions were monitored at 412 nm by using a SpectraMax M5 plate reader (Molecular Devices, San Jose, CA) at 25 °C every 30 s for 10 min. Using the initial rates, the rate of no reaction was subtracted and normalized that value to the rate of no inhibitor. All assays were performed in triplicate. The data was plotted as a sigmoidal curve and IC_50_ values were calculated using SigmaPlot 14.0 (Systat Software, San Jose, CA, USA). The IC_50_ values for *Ee*AChE and *Ef*BChE inhibitions are presented in Table 1 and the corresponding graphs are presented in Figures S66 and S67, respectively.

### 3.4. Materials Used for ^3^H-PIB Binding Assays and the Assays Themselves

#### 3.4.1. Aβ_(1–40)_ and Aβ_(1–42)_ Fibril Assembly

In total, 250 μg of lyophilized NH_4_OH-treated Aβ_(1–40)_ (cat # A-1157-02) and Aβ_(1–42)_ (cat # A-1167-02) purchased from rPeptide (Watkinsville, GA, USA) were each solubilized in their glass vials with 250 μL of a buffer solution comprised of 20 mM NaP_i_, 145 mM NaCl, 0.02% *w*/*v* NaN_3_ at pH 7.5 and incubated at 37 °C for 3 days, vortexing once per day. Prior to being transferred to screw-top polypropylene vials and being stored at −75 °C, these 1 mg/mL fibril suspensions were further diluted with an equal volume of the buffer solution. Their fibril content was determined in aliquots before and after centrifugation at 13,000× *g* by thioflavin fluorescence [73].

#### 3.4.2. ADPBC Isolation

Human AD brain frontal cortical tissue (from the Sanders-Brown Center on Aging Alzheimer’s Disease Center Brain Bank at the University of Kentucky) was processed as follows. The insoluble Pittsburgh Compound B-binding fraction (ADPBC = AD PIB-binding complex) was prepared from the frontal cortex by sequential differential centrifugation of the tissue homogenate followed by sodium dodecyl sulfate extraction [74]. After detergent extraction, the pellet was washed with 20 mM NaP_i_ buffer with 145 mM NaCl at pH 7.5 and collected by centrifugation at 100,000× *g* and aliquots stored in screw-top polypropylene vials at −75 °C.

#### 3.4.3. ^3^H-PIB Binding Assays

Competition of compounds for the binding of ^3^H-PIB (VT 278, specific activity 70.2 Ci/mmol) purchased from Vitrax Radiochemicals (Placentia, CA) to Aβ_(1–40)_ and Aβ_(1–42)_ fibrils and AD brain extract (ADPBC) was determined by radioligand binding. 200 μL of 1.2 nM ^3^H-PIB in 20 mM NaP_i_ buffer with 145 mM NaCl at pH 7.5, and 5% *v*/*v* EtOH-containing compounds and solvent controls were added to 200 ng of Aβ_(1–40)_ or Aβ_(1–42)_ fibrils in 20 μL of 20 mM NaP_i_ buffer with 145 mM NaCl at pH 7.5 in individual wells of a polypropylene 96-well plate (Costar 3355). For the ADPBC, 25 μL of 20 mM NaP_i_ buffer with 145 mM NaCl at pH 7.5 containing the equivalent of 133 μg wet weight of original tissue were incubated with compounds and solvent controls. The plates were sealed and incubated for 3 h at 22 °C, without shaking, and transferred to a 96-well Millipore Multiscreen HTS Hi Flow FB (GF/B) filter plate, and filtered with a multi-well plate vacuum manifold (Millipore Corporation, Bedford, MA, USA). The filters were rapidly washed 4× with 200 μL of 20 mM NaP_i_ buffer with 145 mM NaCl at pH 7.5, and 5% *v*/*v* EtOH, dried, and transferred to scintillation vials and Budget-Solve (Fisher) scintillation fluid was added. Specific binding of ^3^H-PIB was determined by subtracting the non-specific binding determined in the presence of 1 μM BTA-1. These data are presented in Figure 1.

#### 3.4.4. ^3^H-PIB Titration Assays

The titration of compounds with ^3^H-PIB bound to Aβ_(1–40)_ and Aβ_(1–42)_ fibrils and AD brain extract (ADPBC) were performed to further determine the competition of our compounds for the ^3^H-PIB binding site of the AD brain. The experiments were performed as described in the ^3^H-PIB binding assays of Section 3.4.3 with the use of a concentration range of 10 to 0.03 μM for the compounds tested. These data are presented in Figure 2.

### 3.5. Biotinyl-Aβ_(1–42)_ (bioAβ_42_) Oligomer Assembly and Dissociation Assays

#### 3.5.1. Preparation of ELISA Plates Coated with NeutrAvidin^TM^

Each well of ELISA plates (Costar 9018) were coated with 50 μL of 1 μg/mL NeutrAvidin^TM^ in 10 mM NaP_i_ buffer at pH 7.5 overnight at 4 °C. The next day, they were blocked with 200 μL of 20 mM NaP_i_ buffer at pH 7.5 containing 0.145 M NaCl and 0.1% *v*/*v* Tween 20 for at least 2 h at rt. The plates were then stored in their blocked form at 4 °C prior to use for the oligomer assembly and dissociation assays.

#### 3.5.2. Overview of bioAβ_42_ Oligomer Assembly

The assembly of bioAβ_42_ into soluble oligomers was quantified with an ELISA assay in which the soluble oligomers were captured with the NeutrAvidin^TM^ coating the ELISA plates. The bound oligomers were detected with streptavidin-horseradish peroxidase (SA-HRP) [75]. The colorimetric or other signal produced was specific for oligomeric bioAβ_42_ species because the biotin of the NeutrAvidin^TM^-captured monomeric bioAβ_(1–42)_ was complexed with the NeutrAvidin^TM^ and was unable to react with the SA-HRP. Using biotinylated reagents and biotin-binding proteins for capture and detection avoids the interaction of small molecules with antibodies and this selectivity is useful for screening compound libraries [75,76]. Below, please find the details of the three steps (Steps 1–3) used for the oligomer assembly assay.

Step 1: Preparation of monomeric bioAβ_42_. For a low background, it was essential to rigorously pretreat the bioAβ_42_ peptide to convert (disaggregate) the multimeric species that form upon storage in the solid state, or even frozen in 1,1,1,3,3,3-hexafluoroisopropanol (HFIP) to monomers. The required amount of stock bioAβ_42_ peptide (0.5 mg dissolved at 1 mg/mL in HFIP) was pipetted into a polypropylene microfuge tube, dried to a thin film under a gentle air or N_2_ stream, and then disaggregated for 10 min at rt in neat trifluoroacetic acid (TFA). After removal of the TFA by drying with an air or N_2_ stream, DMSO was added to produce a 2.3 μg/mL (50×) stock solution.

Step 2: Assembly of bioAβ_42_ oligomers. 2 μL of disaggregated monomeric 50× bioAβ_42_ peptide in DMSO (prepared in Step 1) was pipetted into the bottom of each well of a polypropylene 96-well plate. The assembly reaction was started by the addition of 100 μL of diluted compound containing up to 1% *v*/*v* DMSO in 20 mM NaP_i_ buffer containing 0.145 M NaCl at pH 7.5. The 96-well plate was sealed and incubated at rt for 30 min without shaking. The bioAβ_42_ oligomer assembly was stopped by adding 50 μL of 0.3% Tween 20 *v*/*v* in distilled H_2_O.

Step 3: Measurement of bioAβ_42_ oligomers. To start measuring the bioAβ_42_ oligomers, the blocking solution for the NeutrAvidin^TM^-coated ELISA plate was removed and 50 μL of bioAβ_42_ oligomers (up to total of 20 nM bioAβ_42_ in 20 mM NaP_i_ buffer with 0.145 M NaCl and 0.1% *v*/*v* Tween 20 at pH 7.5) was added to each well. The 96-well plate was then sealed and incubated for 2 h while shaking at 150 rpm at rt. The plate was then washed 3× with 200 μL of a solution comprised of 20 mM Tris-HCl, 34 mM NaCl, 0.1% *v*/*v* Tween 20 at pH 7.5, and then 50 μL of SA-HRP (1:20,000) in 20 mM NaP_i_ buffer with 0.145 M NaCl and 0.1% *v*/*v* Tween 20 at pH 7.5 was added to each well. The plate was sealed and incubated for 1 h while shaking at 150 rpm at rt. The plate was washed as previously described, and 100 μL/well of HRP substrate (i.e., 1 mM:0.07 mM/tetramethylbenzidine:H_2_O_2_) in 0.2 M citrate buffer, pH 4.0 was added. The plate was incubated at rt for 5 to 10 min and the reaction was stopped by the addition of 100 μL/well of 1% *v*/*v* H_2_SO_4_. The absorbance of each well was read at 450 nm with a BioTek HT Synergy plate reader. These data are presented in Figure 3.

#### 3.5.3. EC_50_ Determination against bioAβ_42_ Oligomer Assembly

The EC_50_ values of hybrids **15a**–**15f** and **16a**–**16f** against bioAβ_42_ oligomer assembly was determined as described in the overview of bioAβ_42_ oligomer assembly in Section 3.5.2. A concentration range of 50 to 1.56 μM for the compounds was used in this study. These data are presented in Figure 4 and Table S1.

#### 3.5.4. Overview of bioAβ_42_ Oligomer Dissociation

Dissociation of preformed oligomers of bioAβ_42_ was measured by pretreating biotinylated oligomers with solvent controls or compounds for a period of time, capturing them with NeutrAvidin^TM^ on ELISA plates, and detecting the remaining bound oligomers with SA-HRP [77] as described above. Below, please find the details of the two steps (Steps 1 and 2) used for the oligomer dissociation assay.

Step 1: Preformed bioAβ_42_ oligomers (sufficient for 400 wells). In total, 1 μg of bioAβ_42_ peptide (1 mg/mL dissolved in HFIP) was pipetted into 20 μL of HFIP in a polypropylene microfuge tube, and dried to a thin film under a gentle air or N_2_ stream. The film was dissolved in 250 μL of DMSO and vortexed. After 10 min at rt with intermittent vortexing, the DMSO-solubilized bioAβ_42_ was added rapidly to 12.5 mL of 20 mM NaP_i_ buffer with 0.145 M NaCl at pH 7.5 in a 17 × 100 mm polypropylene tube, sealed, and vortexed. After incubation at rt for 1 h with intermittent vortexing, 0.375 mL of 100 mM Tween 20 (10% *v*/*v*) in distilled H_2_O was added to make the final Tween 20 concentration 0.3% *v*/*v*. The preformed oligomers were mixed by inversion and aliquoted for storage at −75 °C. Note: Multiple freeze-thaw cycles should be avoided.

Step 2: Oligomer dissociation assay. Preformed bioAβ_42_ oligomers (16.3 nM total bioAβ_42_) in Tween 20 were added to diluted compound in 20 mM NaP_i_ buffer with 0.145 M NaCl at pH 7.5 (final concentration of bioAβ_42_ = 2.7 nM, ≤1% *v*/*v* DMSO) and incubated overnight (~18 h) with shaking. Then, 25 μL of the preformed bio42 oligomers adjusted to 0.6% *v*/*v* Tween 20 was pipetted into each well of a polypropylene 96-well plate. In total, 125 μL of diluted compound in 20 mM NaP_i_ buffer with 0.145 M NaCl at pH 7.5 containing up to 1% *v*/*v* DMSO were added to the oligomers in the plates. The plates were sealed and shaken at 150 rpm overnight at rt. After 18 h of incubation, the plates were centrifuged at 1000× *g* for 10 min and a 100-μL aliquot was transferred from each well into a well of an NA-coated ELISA plate that had been blocked with Tween 20. The oligomer content of that aliquot was measured as described for the bioAβ_42_ oligomer assembly assay. These data are presented in Figure 5.

#### 3.5.5. Determination of the Effects of Chalcone and Donepezil Fragments (Alone or in Combination) on Biotinyl-Aβ_(1–42)_ (bioAβ_42_) Oligomer Assembly and Dissociation

The effects of various chalcones, donepezil, donepezil analogue, and 1,3-chalcone-donepezil hybrids were tested for their ability to inhibit the bioAβ_42_ oligomer assembly and dissociation either alone or in combination with each other. The experiments were performed as described in the biotinyl-Aβ_(1–42)_ (bioAβ_42_) oligomer assembly and dissociation assays section. 1:1 or 1:1:1 ratios of the compounds were used, and these data are presented in Figure 6 and Figure 7.

### 3.6. Molecular Modeling

In the modeling of the *Hs*AChE-**15a**-Aβ complex, we used the crystal structure of the *Hs*AChE-donepezil complex (PDB ID: 4EY7 [66]) and the cryo-electron microscopy (cryo-EM) structure of an Aβ fibril (PDB ID: 6SHS [67]). The modeling was performed with Crystallographic Oriented Toolkit (Coot [78]) software. To generate the model, we superimposed the donepezil moiety of **15a** with the bound donepezil in the *Hs*AChE-donepezil crystal structure. The Aβ fibril was stripped down to two layers of Aβ and manually docked onto the chalcone side of **15a** in an extended conformation while avoiding steric clashes and maximizing electrostatic and hydrophobic interactions between **15a** and Aβ, assuming that the amino group of **15a** would interact with the highly negatively-charged surface of Aβ defined by adjacent residues Glu22 and Asp23.

## 4. Conclusions

In summary, we synthesized 12 novel 1,3- and 1,4-chalcone-donepezil hybrids, **15a**–**15f** and **16a**–**16f**, and evaluated their ability to inhibit AD therapeutic targets. In general, most of these compounds were able to inhibit the actions of *Ee*AChE and *Ef*BChE with low to sub-micromolar IC_50_ values. In general, 1,4-chalcone-donepezil hybrids (**16**) were better at inhibiting *Ee*AChE and *Ef*BChE than 1,3-chalcone-donepezil hybrids (**15**), with the exception of **15f** against *Ee*AChE and **15a** and **15b** against *Ef*BChE. The presence of shorter linkers between chalcones and donepezil seemed to have a positive impact on *Ee*AChE and *Ef*BChE inhibition. In the displacement of ^3^H-PIB from F_40_, F_42_, and ADPBC fibrils assays, we found that 1,3- and 1,4-chalcone-donepezil hybrids did not displace ^3^H-PIB as effectively as chalcones **3** and **6**, which pointed towards a different binding site for these hybrids compared to PIB. Compared to donepezil and parent 1,3- and 1,4-chalcones **3** and **6**, compounds **15a**, **15b**, **15c**, **15d**, **16a**, **16b**, **16c**, and **16d** exhibited better inhibition of bioAβ_42_ oligomer assembly. However, none of these 1,3- and 1,4-chalcone-donepezil hybrids were effective at dissociating preformed bioAβ_42_ oligomers. Finally, we performed a combination study that convincingly showed that a covalent linkage between chalcones and donepezil is essential for the prevention of bioAβ_42_ oligomerization. These studies demonstrate the promise of chalcone-donepezil hybrids as bifunctional molecules against two hallmarks of AD.

## Figures and Tables

**Table 1:** Inhibition (IC_50_ in μM) of *Ee*AChE (from *Electrophorus electricus*) and *Ef*BChE (from *Equus ferus*) by compounds **15a**–**15f** and **16a**–**16f** as well as the selectivity index (SI) for each inhibitor based on their IC_50_ values.

Cpd	*Ee*AChE	*Ef*BChE	SI
Donepezil·HCl	0.12 ± 0.01	2.0 ± 0.1	17
**15a**	1.02 ± 0.33	0.020 ± 0.002	0.02
**15b**	0.65 ± 0.14	0.03 ± 0.01	0.05
**15c**	1.22 ± 0.18	≈4	≈3.3
**15d**	1.22 ± 0.13	≈2	≈1.6
**15e**	1.82 ± 0.45	≈10	≈5.5
**15f**	1.67 ± 0.80	>100	>60
**16a**	0.07 ± 0.01	≈0.1	≈1.4
**16b**	0.14 ± 0.02	≈0.5	≈3.6
**16c**	≈0.5	≈0.6	≈1.2
**16d**	0.52 ± 0.06	≈1.0	≈1.9
**16e**	1.38 ± 0.18	≈5.0	≈3.8
**16f**	>100	>100	1

These experiments were performed in triplicate (*n* = 1 independent experiment). The errors represent standard deviations (SDEV).

## Supplementary Materials

The following are available online, the Supplementary Materials include ^1^H and ^13^C NMR spectra for all the molecules synthesized (Figures S1–S65). The IC_50_ curves for the inhibition of *Ee*AChE (Figure S66) and *Ef*BChE (Figure S67) are also provided.

## Abbreviations

Aβ	amyloid-β
ACh	acetylcholine
AChEI	acetylcholinesterase inhibitor
AD	Alzheimer’s disease
ChE	cholinesterase
EC_50_	half maximal effective concentration
*Ee*AChE	acetylcholinesterase (from *Electrophorus electricus*)
*Ef*BChE	butyrylcholinesterase (from *Equus ferus*)
*Hs*AChE	acetylcholinesterase (from *Homo sapiens*)
IC_50_	half maximal inhibitory concentration
KOH	potassium hydroxide
MsOH	methanesulfonic acid
NMDA	*N*-methyl-d-aspartate
PIB	Pittsburgh Compound B
ROS	reactive oxygen species
SAR	structure-activity relationship
SDEV	standard deviation
TBDMS	*tert*-butyldimethylsilyl
