# Supplementary material for: Combining Chalcones with Donepezil to Inhibit Both Cholinesterases and Aβ Fibril Assembly
Source: Molecules. 2019 Dec 24;25(1):77. doi: 10.3390/molecules25010077 (PMC6983213; doi:10.3390/molecules25010077)
Supplement: Supplementary file 1 [file molecules-25-00077-s001.pdf]

# Supplementary Materials

## Combining chalcones with donepezil to inhibit both cholinesterases and Aβ fibril assembly

Nishad Thamban Chandrika,[a] Marina Y. Fosso,[a] Oleg V. Tsodikov,[a] Harry LeVine III,[b,c] and Sylvie Garneau-Tsodikova[a,]*

[a]Department of Pharmaceutical Sciences, College of Pharmacy, University of Kentucky, Lexington, KY, 40536-0596, USA. [b]Center on Aging and [b]Department of Molecular and Cellular Biochemistry, School of Medicine, University of Kentucky, Lexington, KY, 40536-0230, USA.

* Correspondence to Sylvie Garneau-Tsodikova (E-mail: sylviegtsodikova@uky.edu)

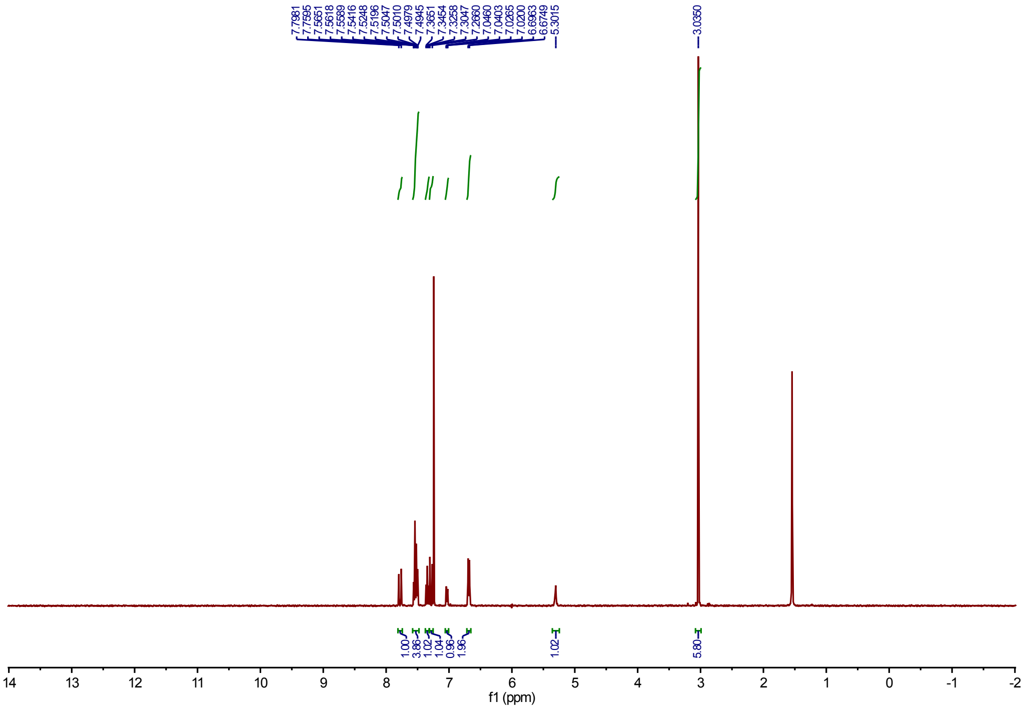


**Figure S1.** $^{1}H$ NMR spectrum for compound **3** in $CDCl_3$ (400 MHz).

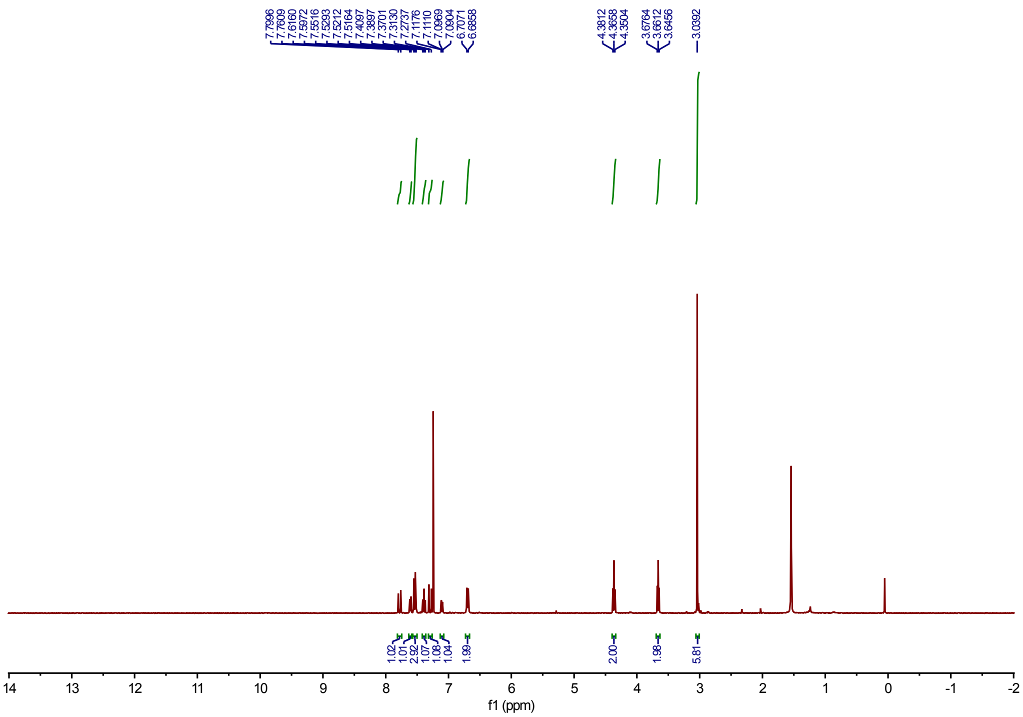

**Figure S2.** $^{1}$H NMR spectrum for compound **4a** in $CDCl_3$ (400 MHz).

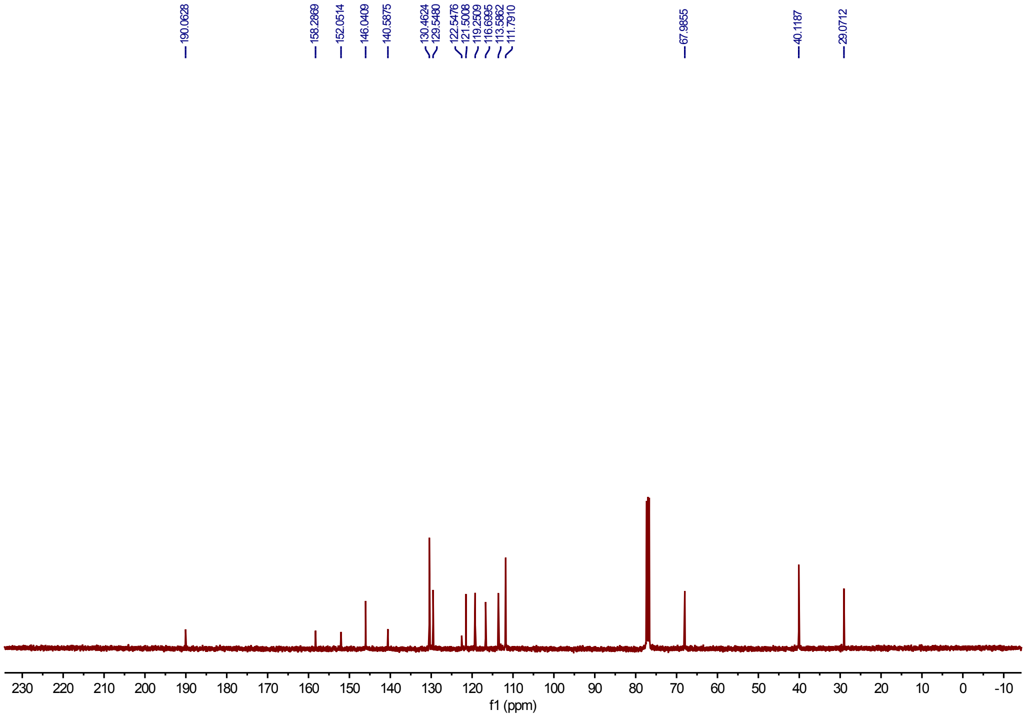

**Figure S3.** $^{13}$C NMR spectrum for compound **4a** in $CDCl_3$ (100 MHz).

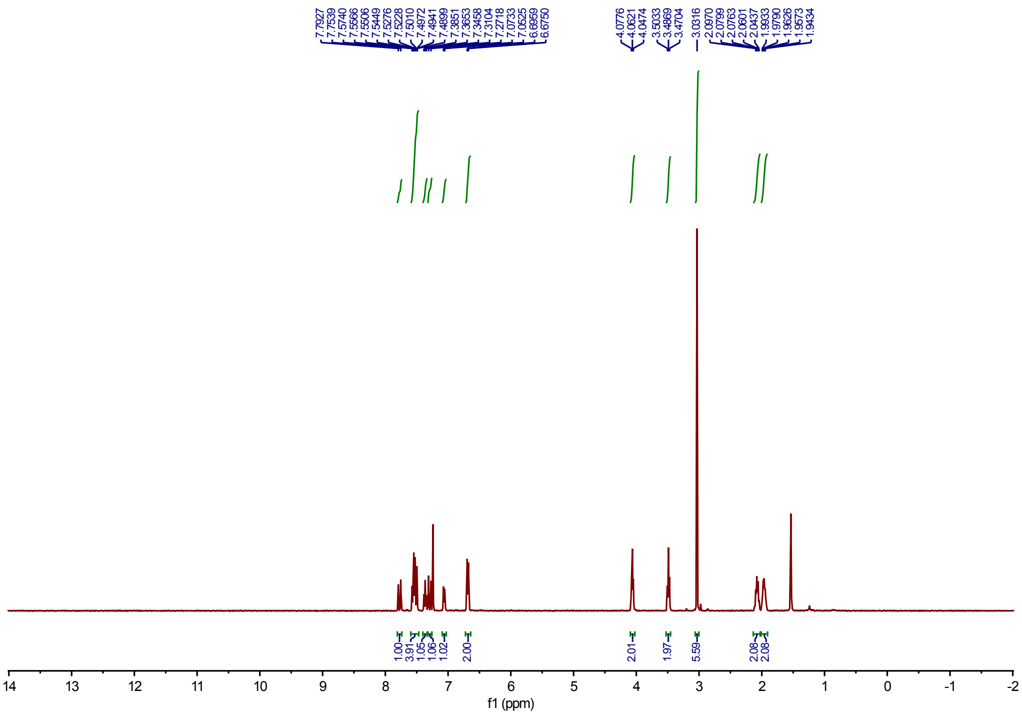


**Figure S4.** $^{1}$H NMR spectrum for compound **4b** in $CDCl_3$ (400 MHz).

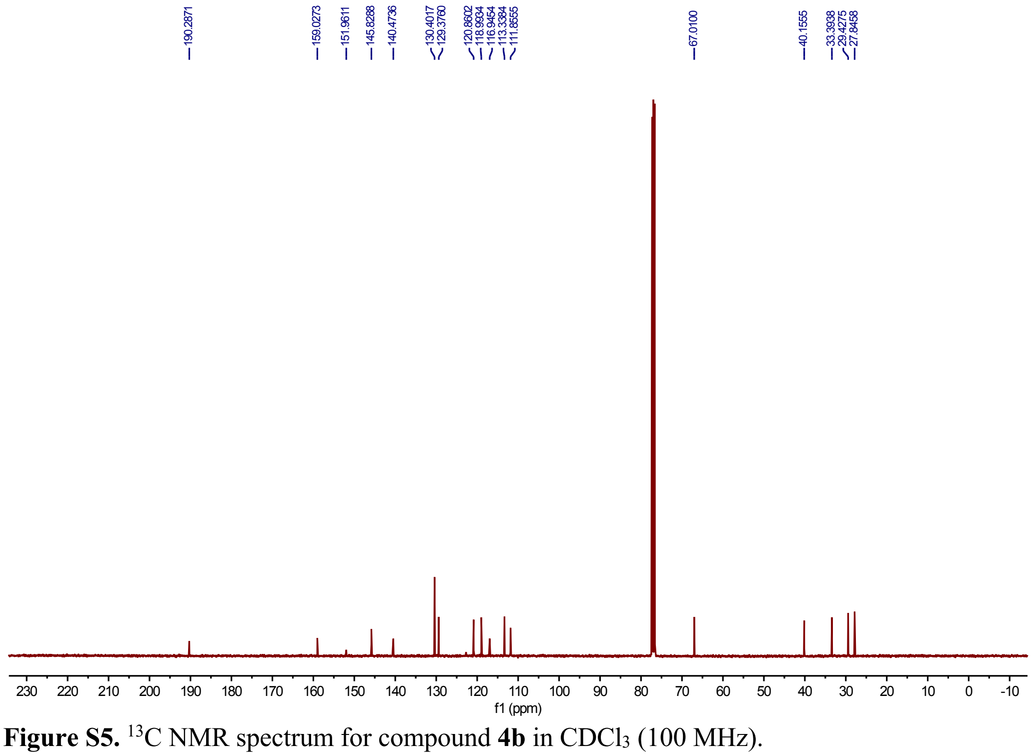


**Figure S5.** $^{13}$C NMR spectrum for compound **4b** in $CDCl_3$ (100 MHz).

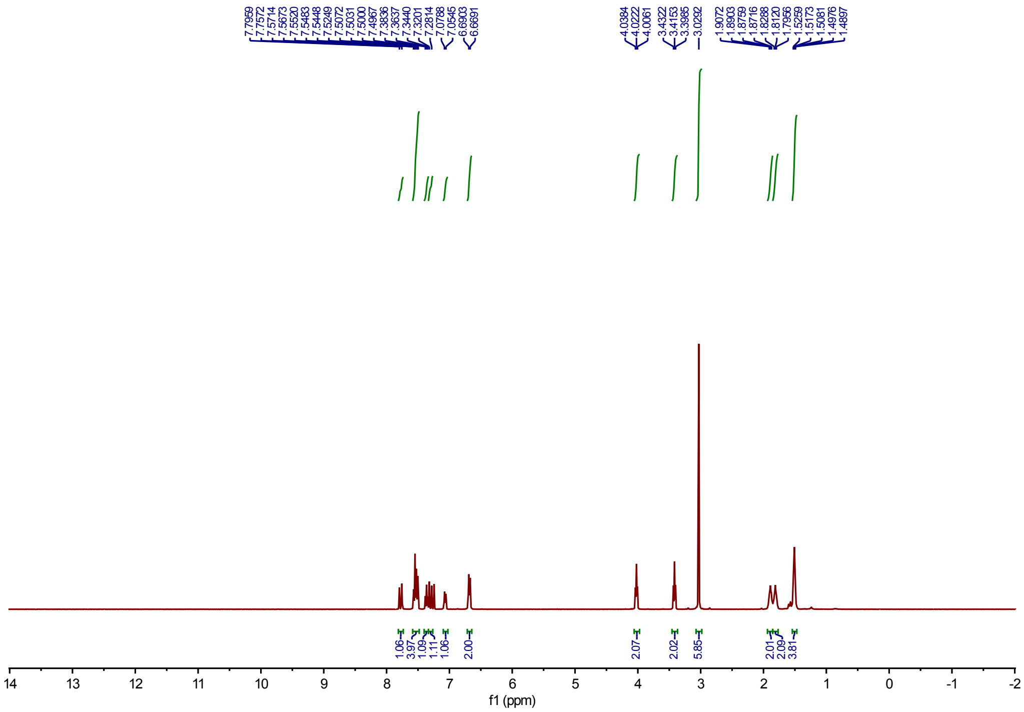


**Figure S6.** $^{1}$H NMR spectrum for compound **4c** in $CDCl_3$ (400 MHz).

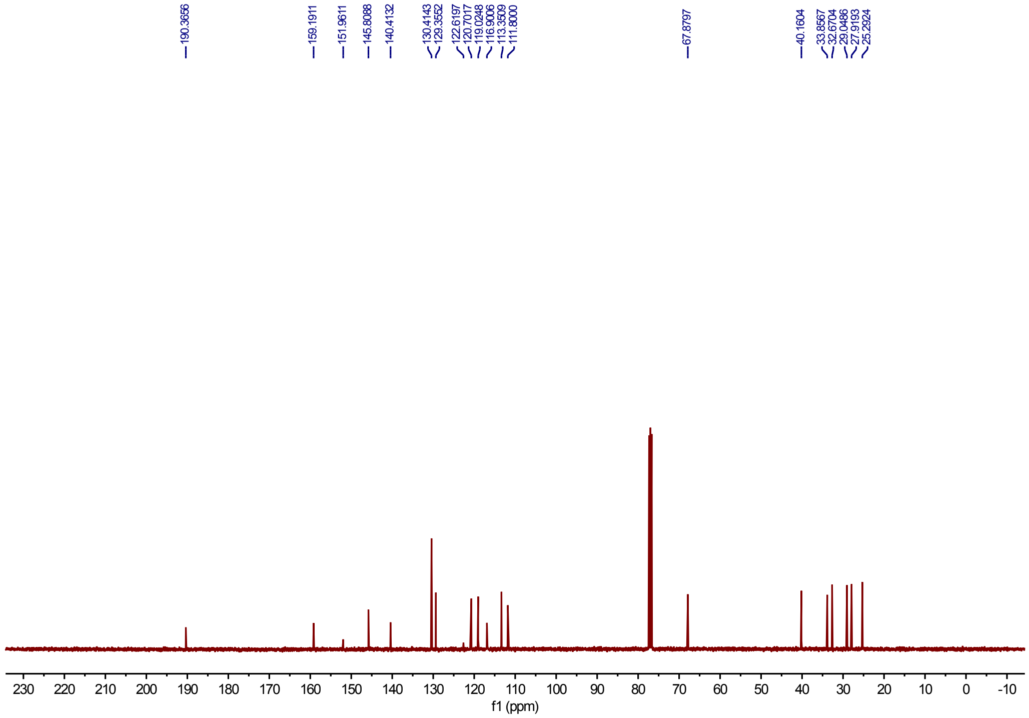


**Figure S7.** $^{13}$C NMR spectrum for compound **4c** in $CDCl_3$ (100 MHz).

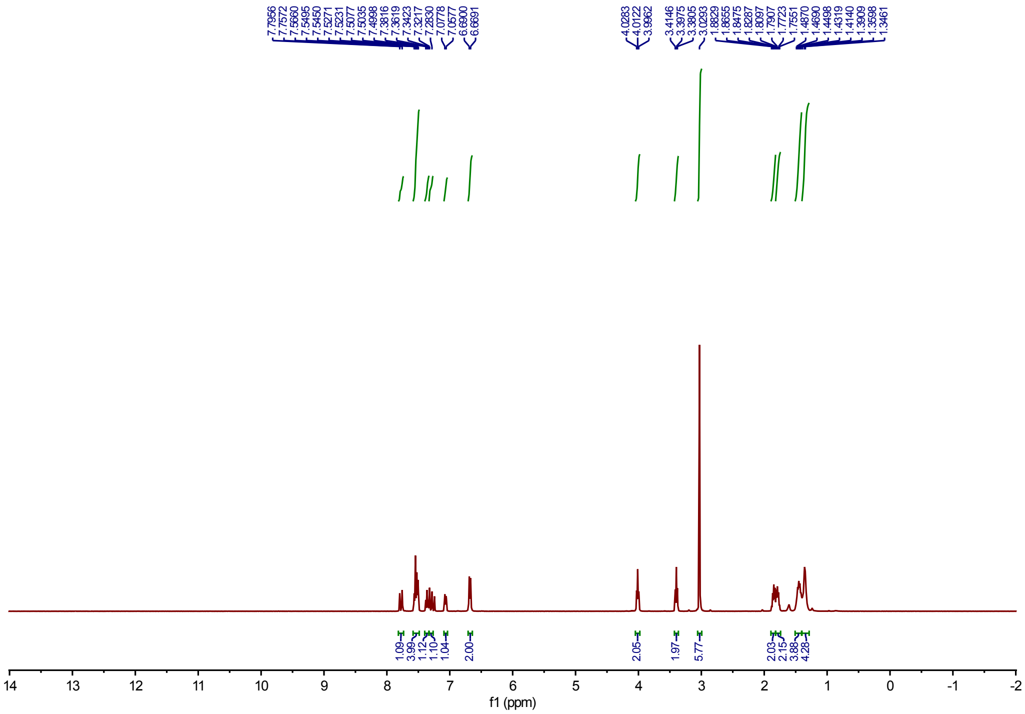

**Figure S8.** $^{1}$H NMR spectrum for compound **4d** in $CDCl_3$ (400 MHz).

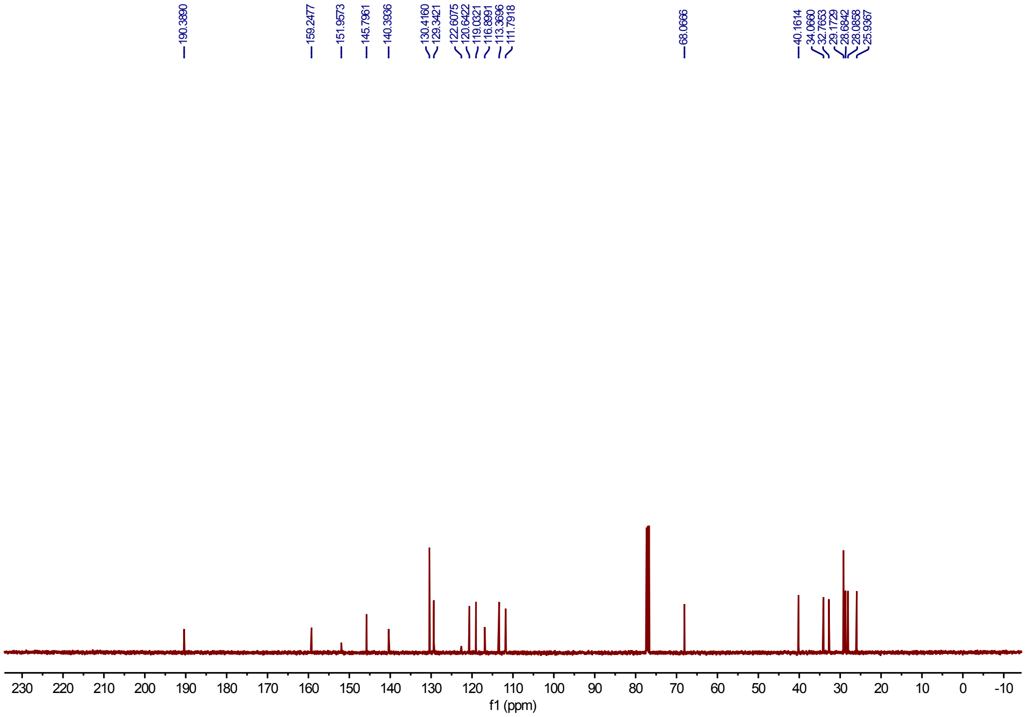

**Figure S9.** $^{13}$C NMR spectrum for compound **4d** in $CDCl_3$ (100 MHz).

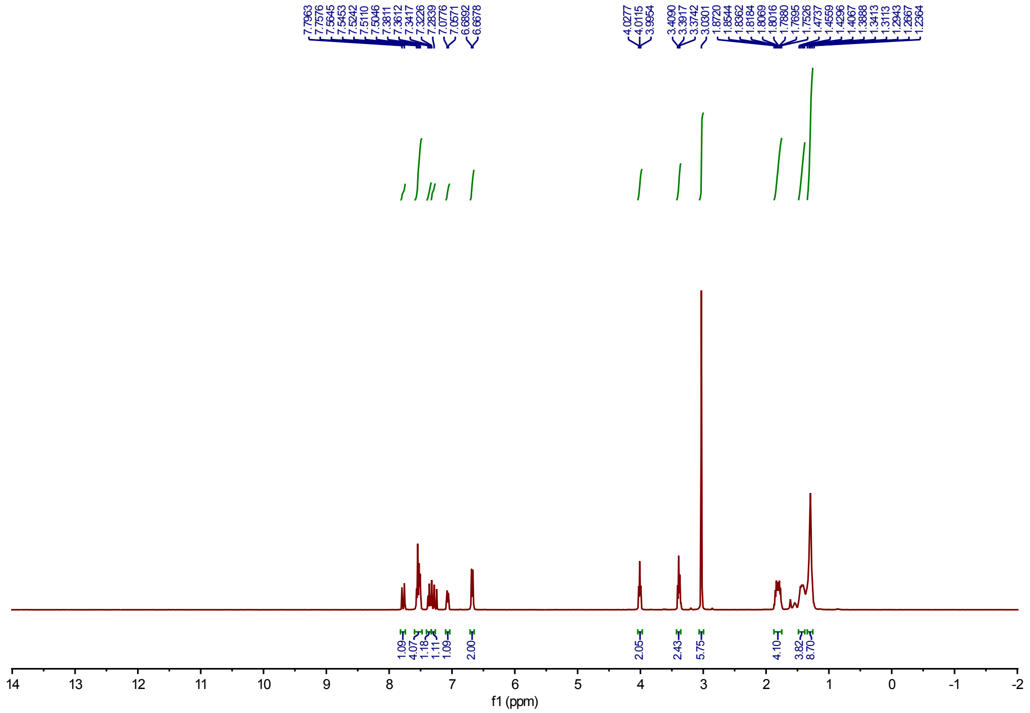

**Figure S10.** $^{1}H$ NMR spectrum for compound **4e** in $CDCl_3$ (400 MHz).

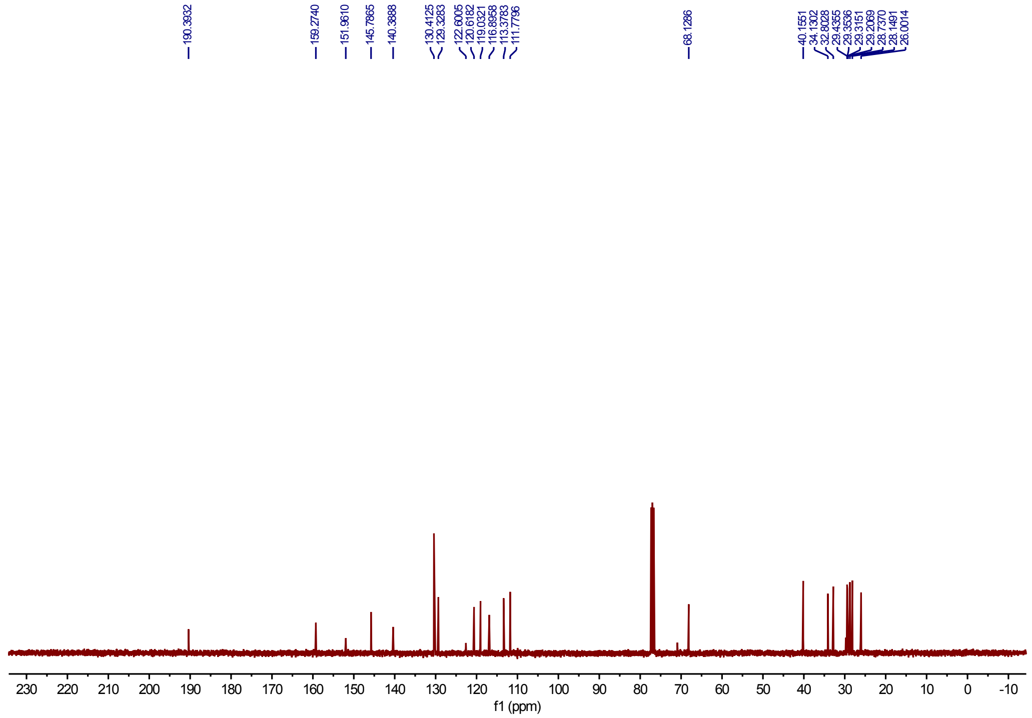

**Figure S11.** $^{13}C$ NMR spectrum for compound **4e** in $CDCl_3$ (100 MHz).

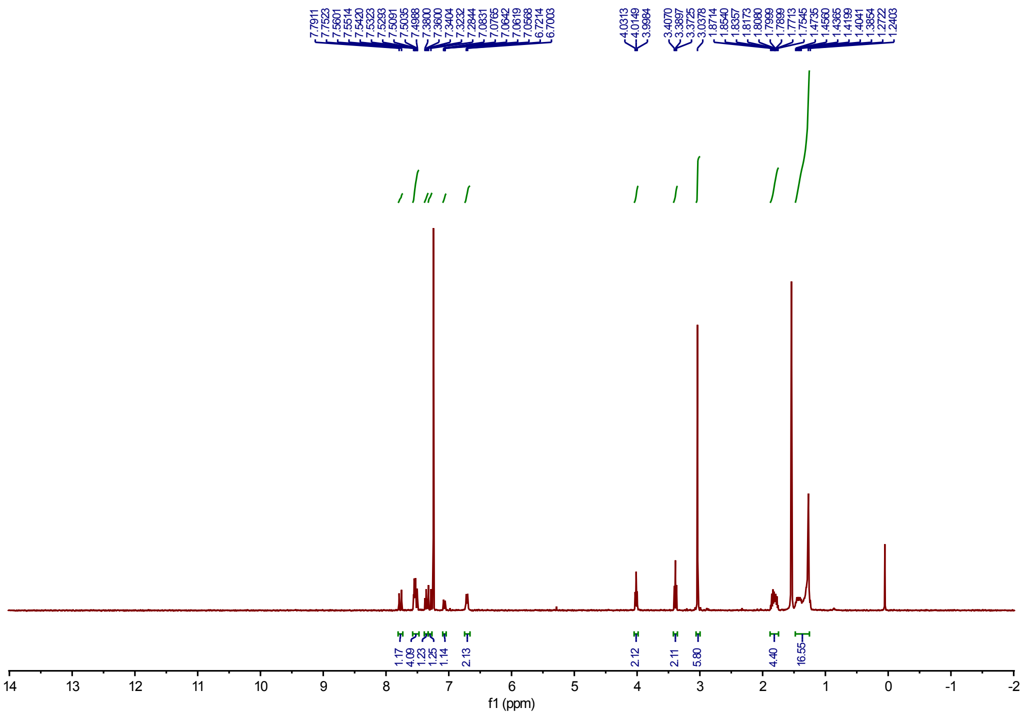


**Figure S12.** $^{1}$H NMR spectrum for compound **4f** in $CDCl_3$ (400 MHz).

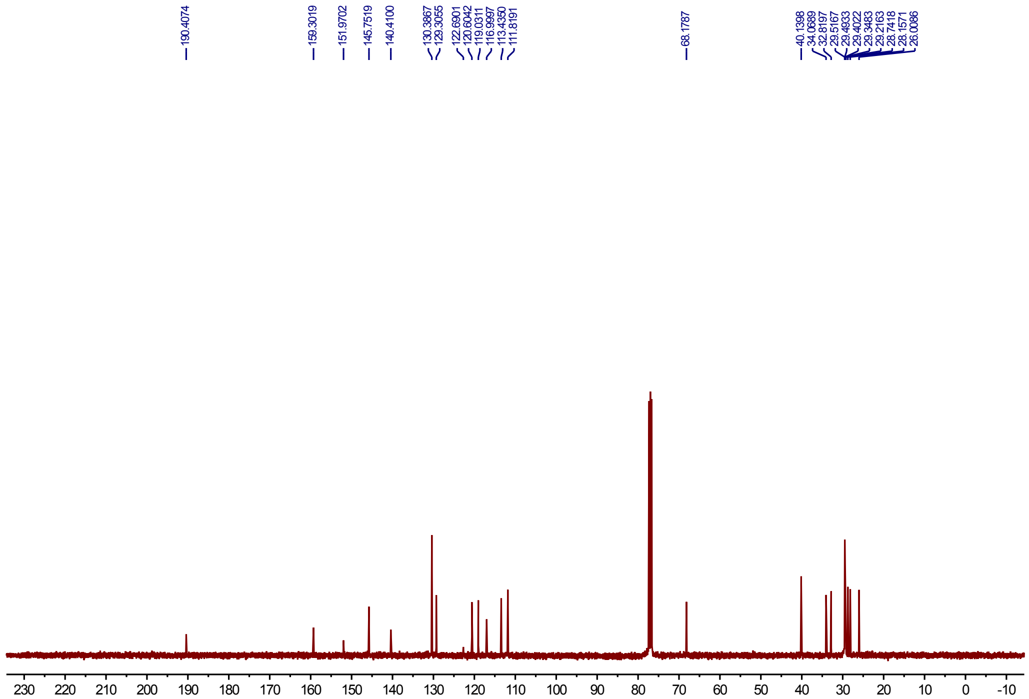


**Figure S13.** $^{13}$C NMR spectrum for compound **4f** in $CDCl_3$ (100 MHz).

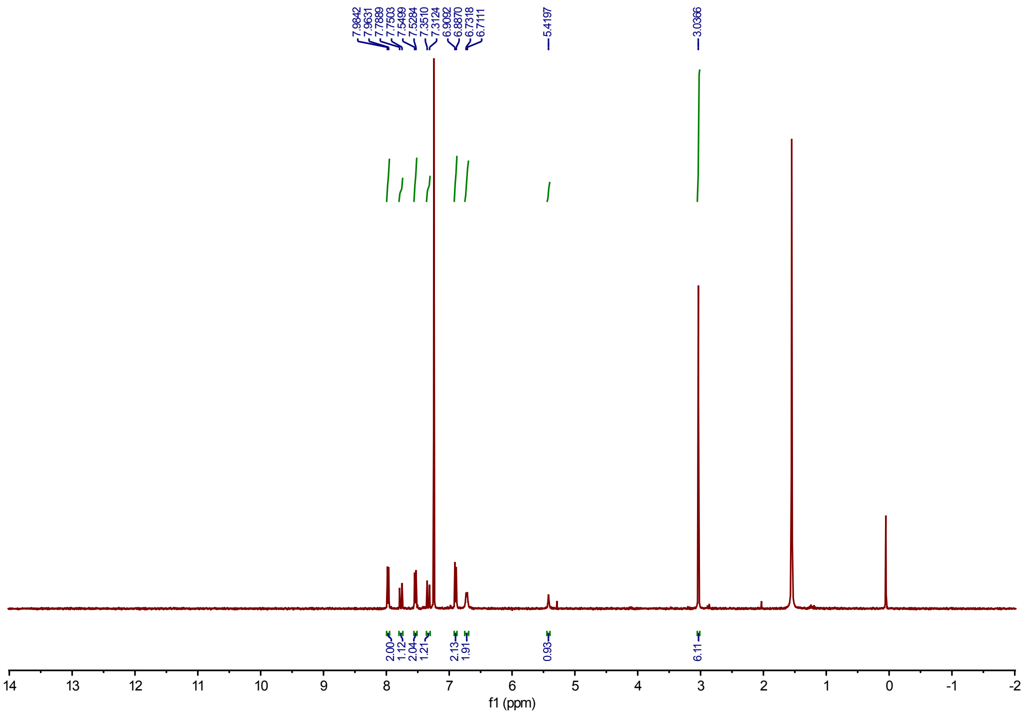

**Figure S14.** $^{1}$H NMR spectrum for compound **6** in $CDCl_3$ (400 MHz).

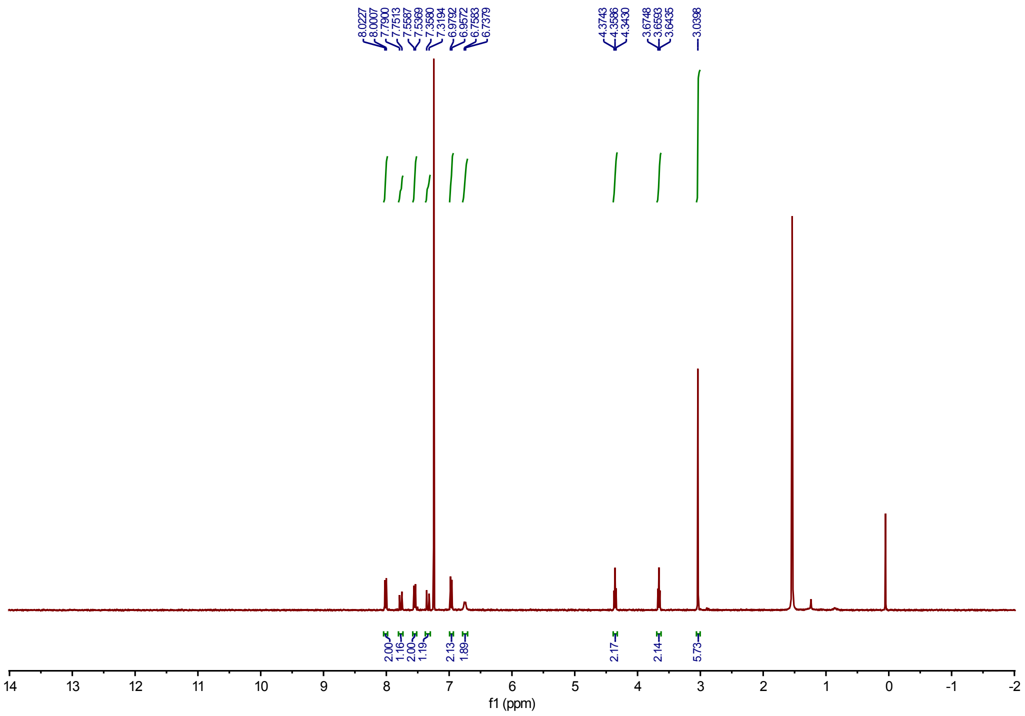

**Figure S15.** $^{1}$H NMR spectrum for compound **7a** in $CDCl_3$ (400 MHz).

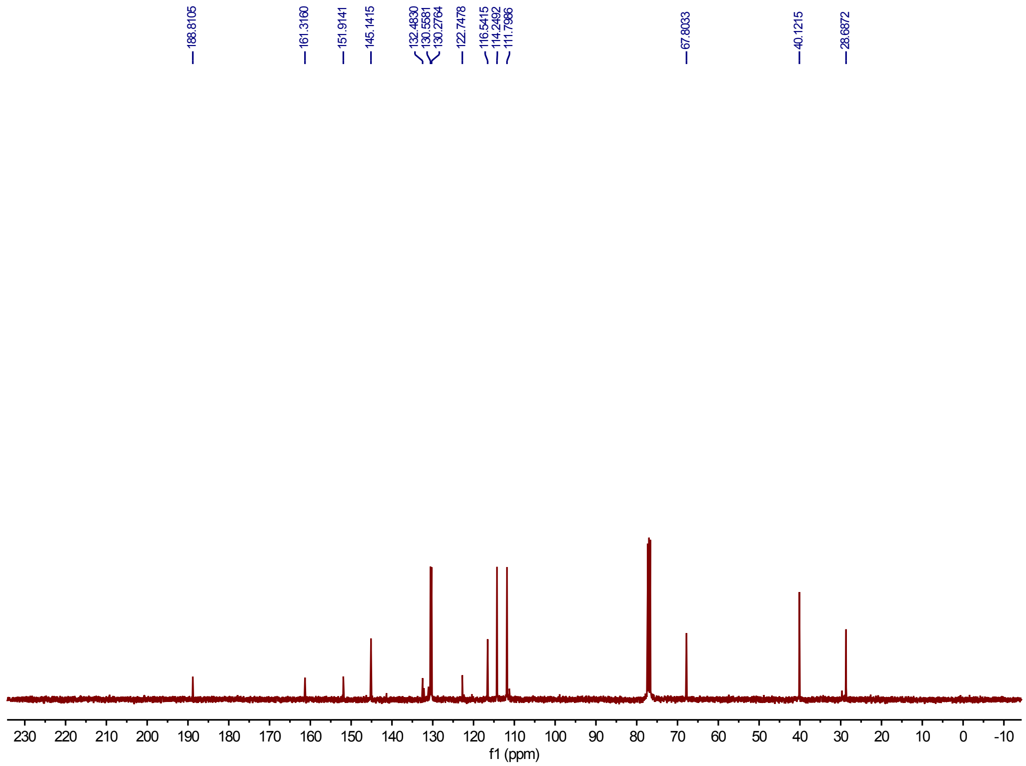


**Figure S16.** $^{13}C$ NMR spectrum for compound **7a** in $CDCl_3$ (100 MHz).

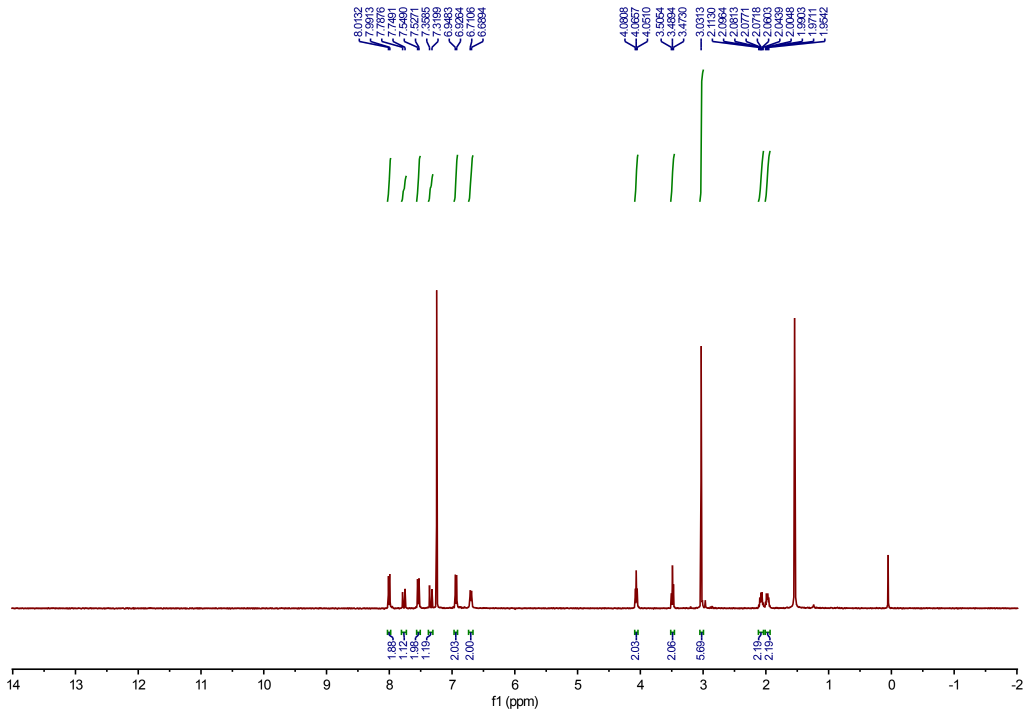


**Figure S17.** $^{1}H$ NMR spectrum for compound **7b** in $CDCl_3$ (400 MHz).

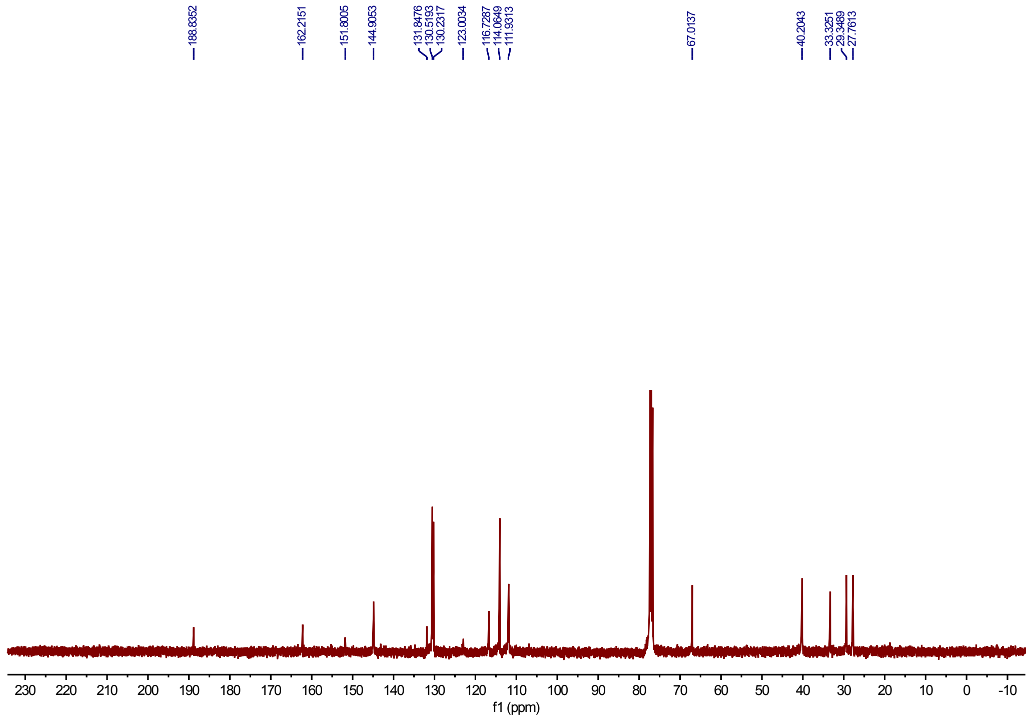


**Figure S18.** $^{13}C$ NMR spectrum for compound **7b** in $CDCl_3$ (100 MHz).

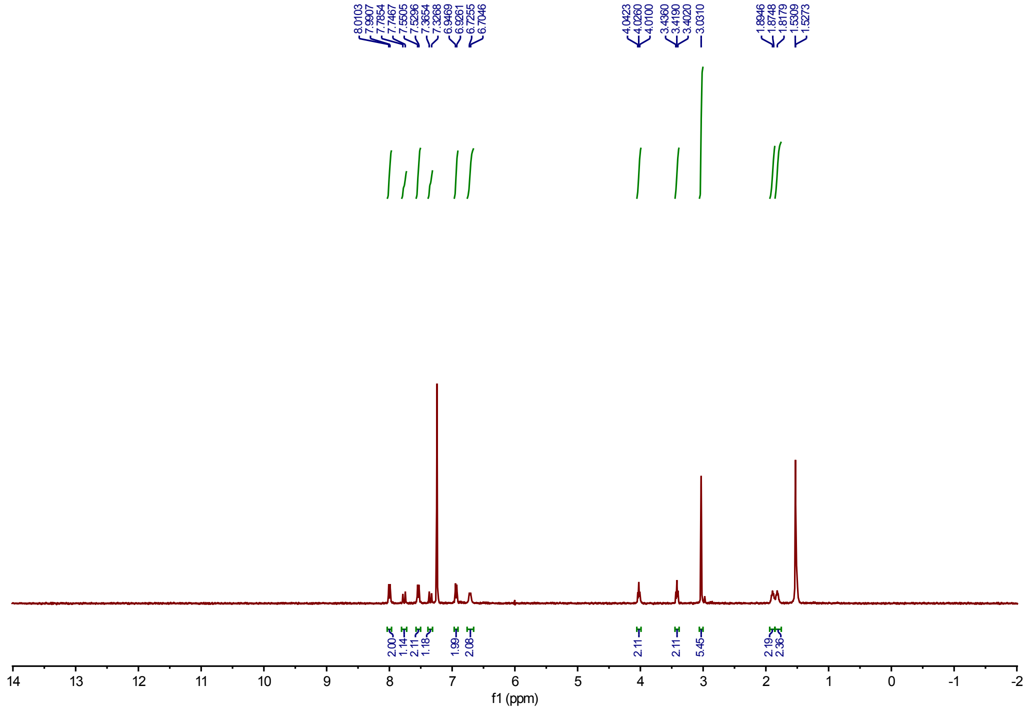


**Figure S19.** $^{1}H$ NMR spectrum for compound **7c** in $CDCl_3$ (400 MHz).

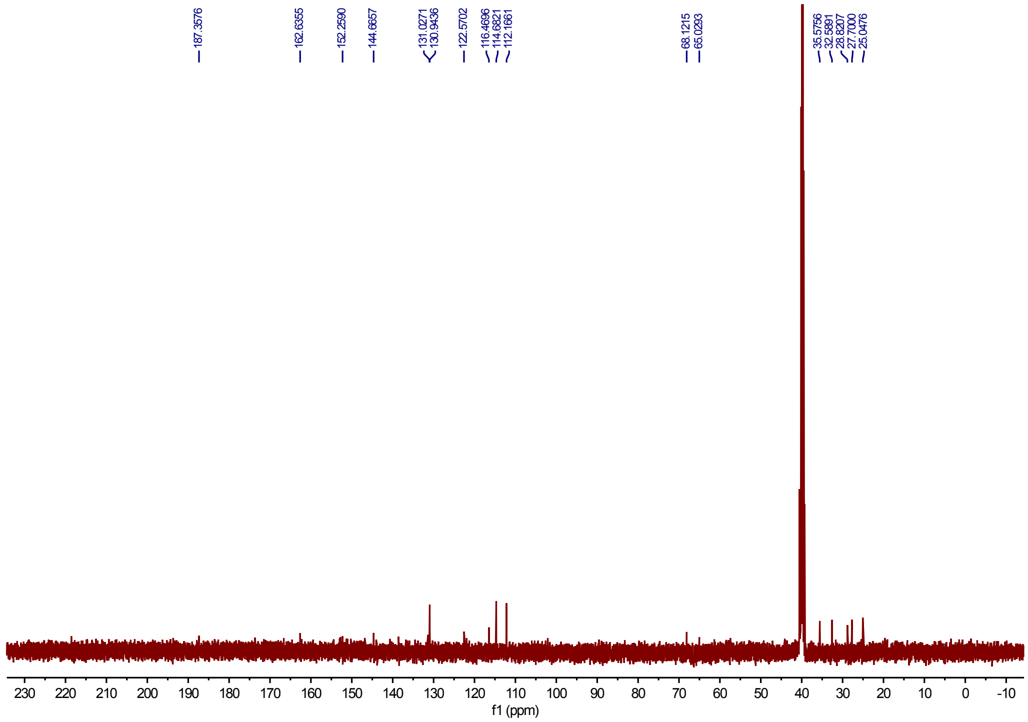


**Figure S20.** $^{13}C$ NMR spectrum for compound **7c** in $(CD_3)_2SO$ (100 MHz).

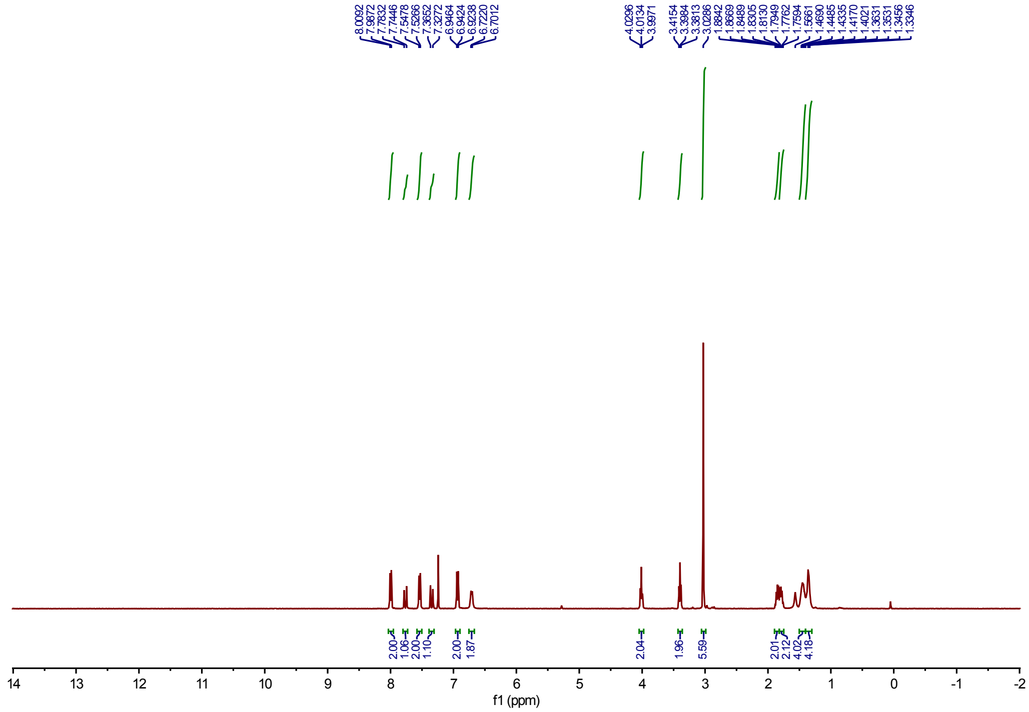


**Figure S21.** $^{1}H$ NMR spectrum for compound **7d** in $CDCl_3$ (400 MHz).

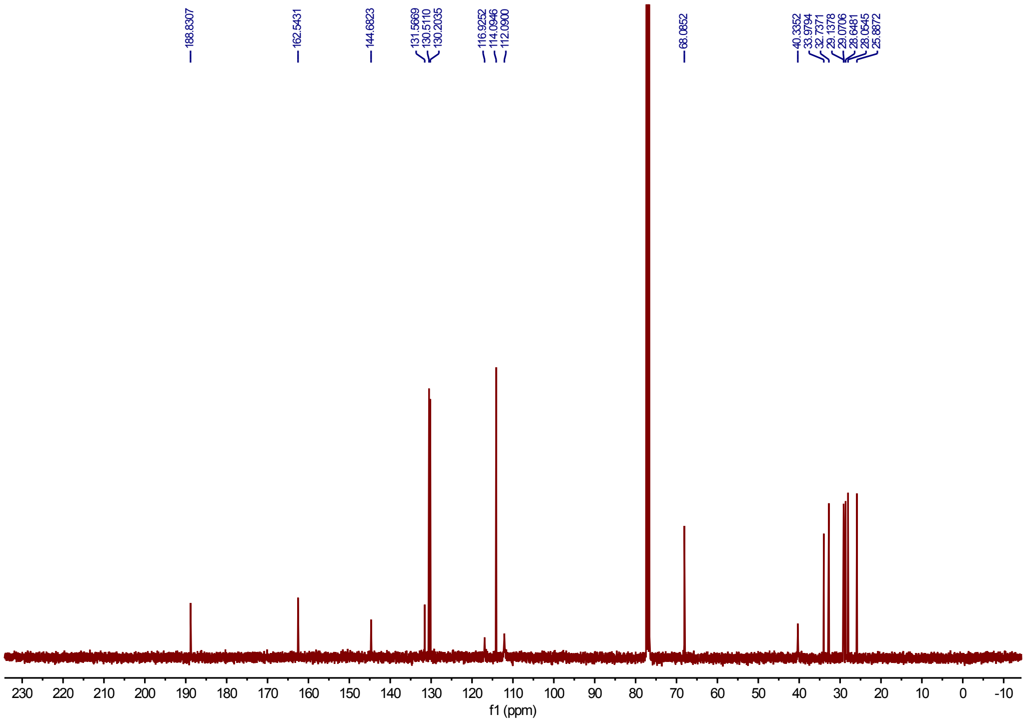


**Figure S22.** $^{13}$C NMR spectrum for compound **7d** in $CDCl_3$ (100 MHz).

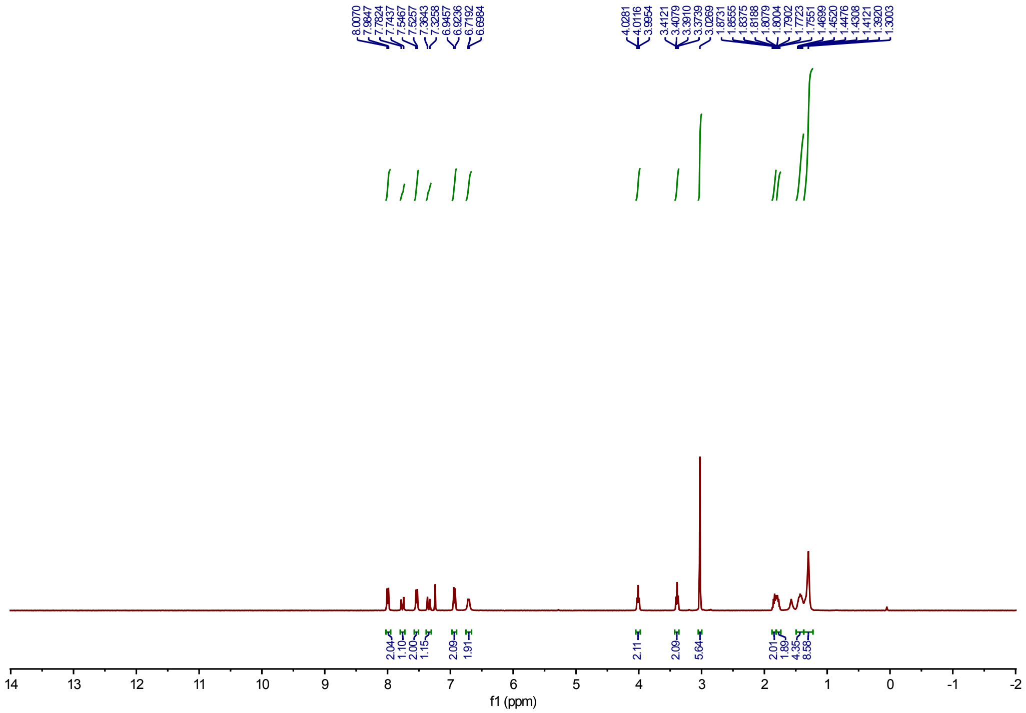


**Figure S23.** $^{1}$H NMR spectrum for compound **7e** in $CDCl_3$ (400 MHz).

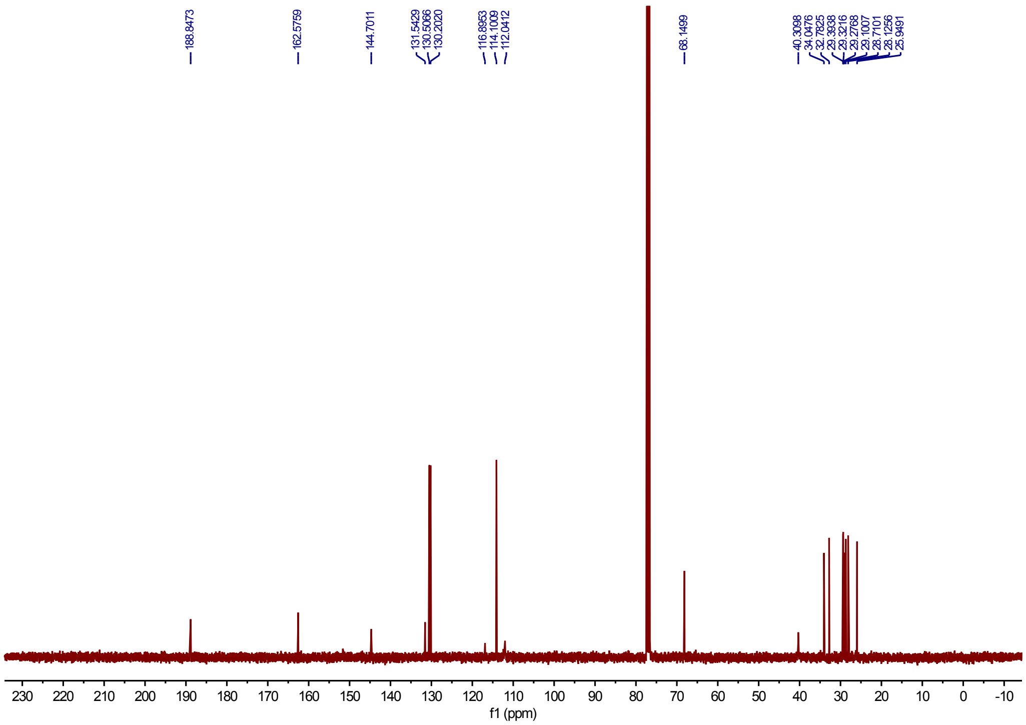


**Figure S24.** $^{13}C$ NMR spectrum for compound **7e** in $CDCl_3$ (100 MHz).

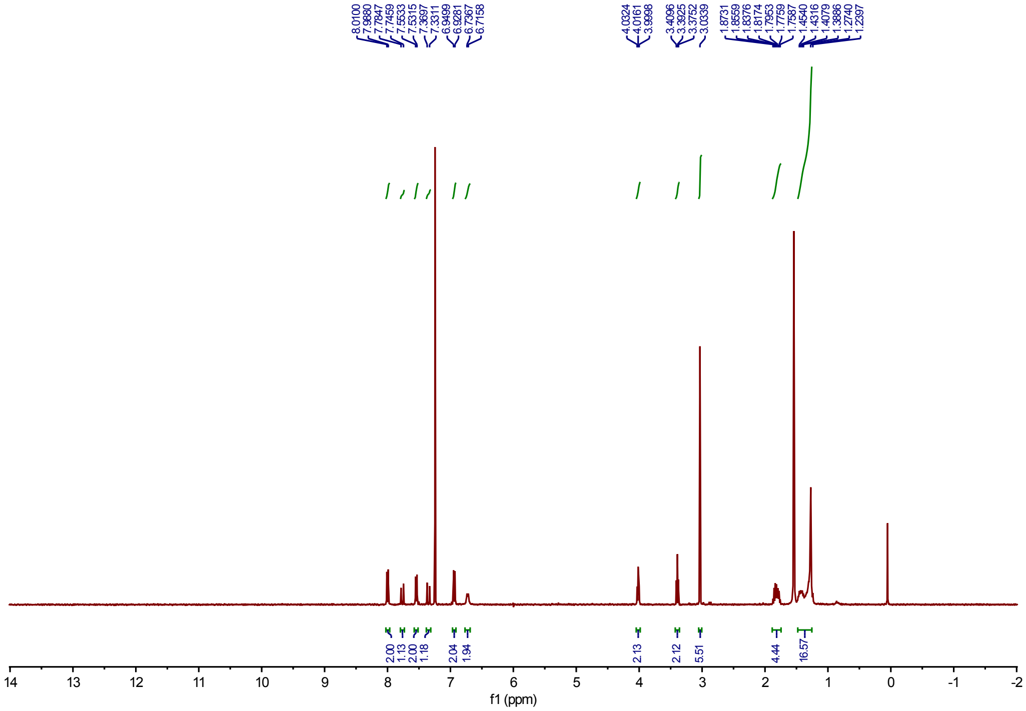


**Figure S25.** $^{1}H$ NMR spectrum for compound **7f** in $CDCl_3$ (400 MHz).

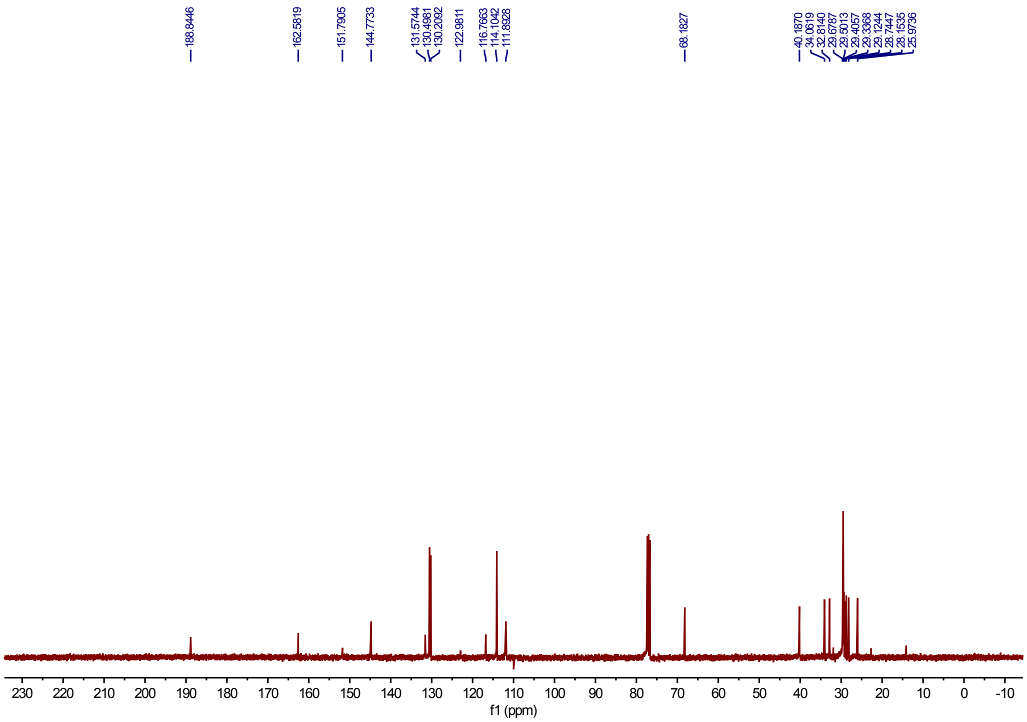


**Figure S26.** $^{13}C$ NMR spectrum for compound **7f** in $CDCl_3$ (100 MHz).

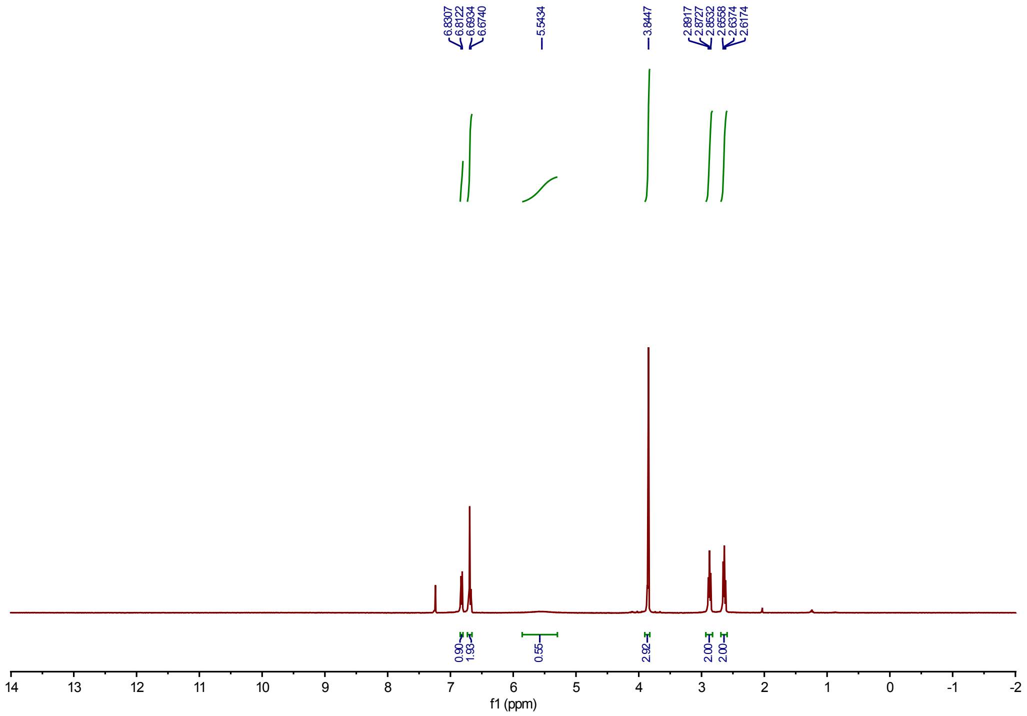


**Figure S27.** $^{1}H$ NMR spectrum for compound **9** in $CDCl_3$ (400 MHz).

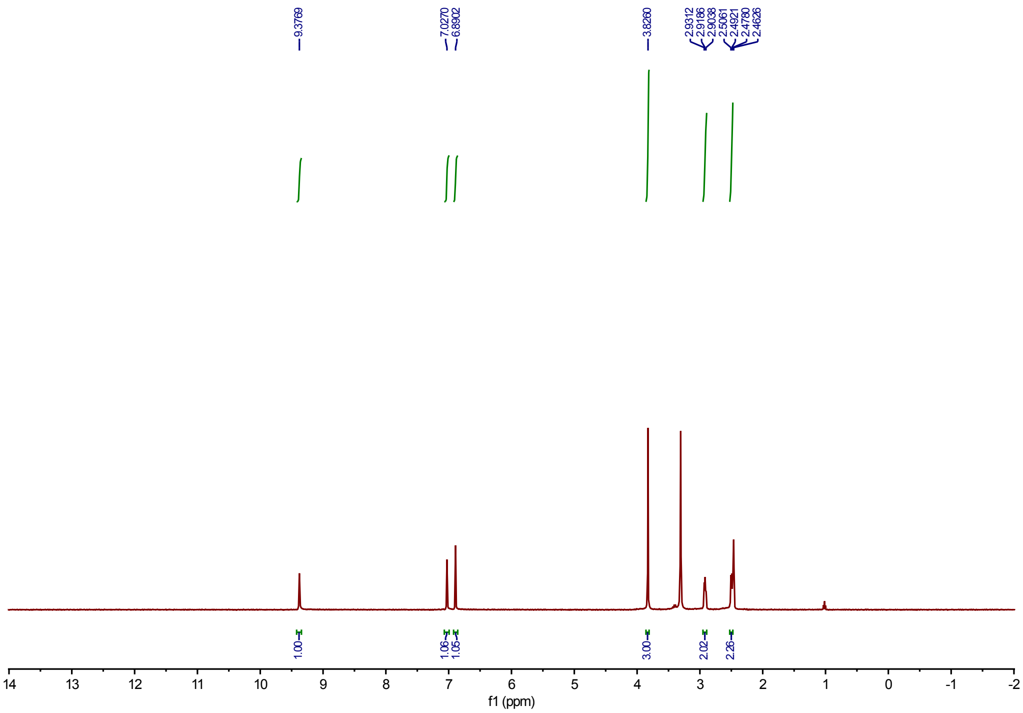


**Figure S28.** $^1$H NMR spectrum for compound **10** in $(CD_3)_2SO$ (400 MHz).

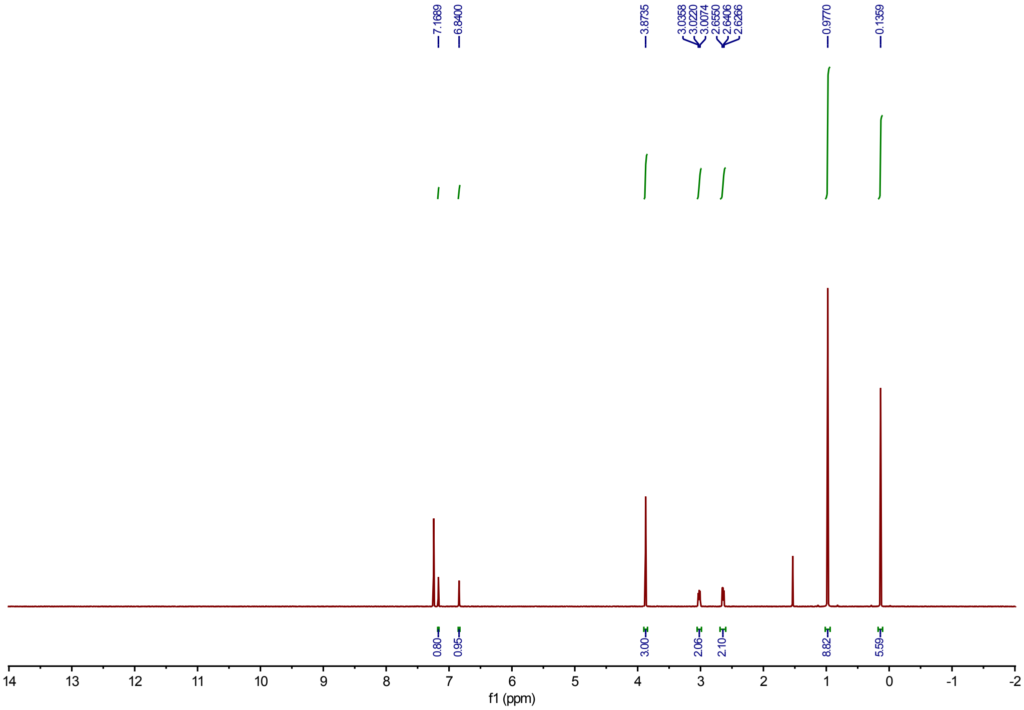


**Figure S29.** $^1$H NMR spectrum for compound **11** in $CDCl_3$ (400 MHz).

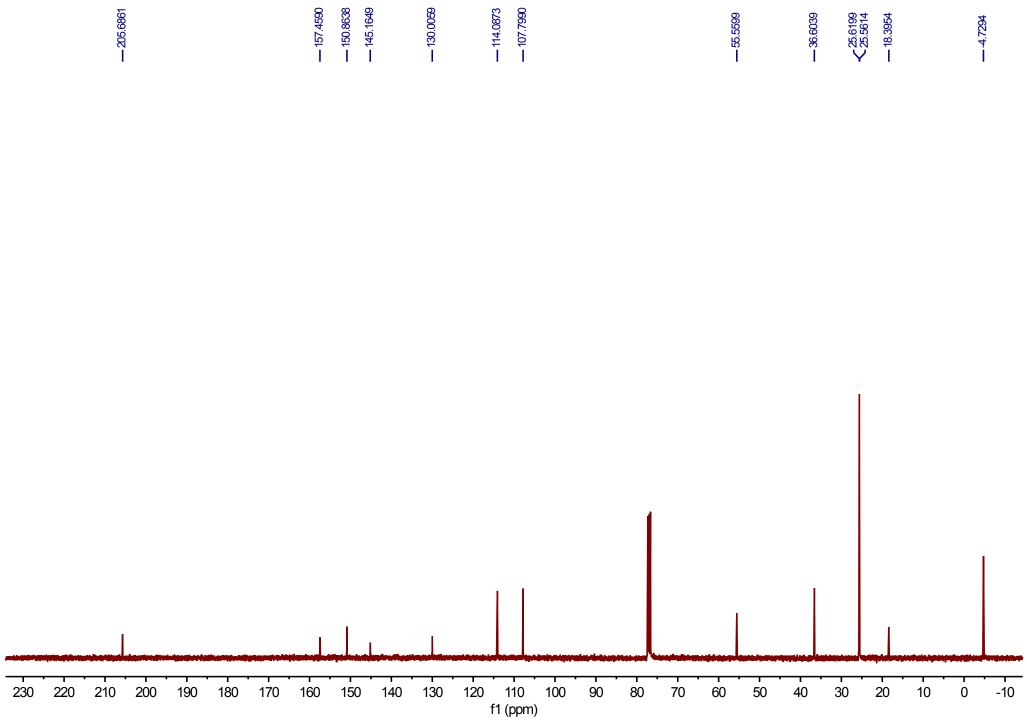

**Figure S30.** $^{13}C$ NMR spectrum for compound **11** in $CDCl_3$ (100 MHz).

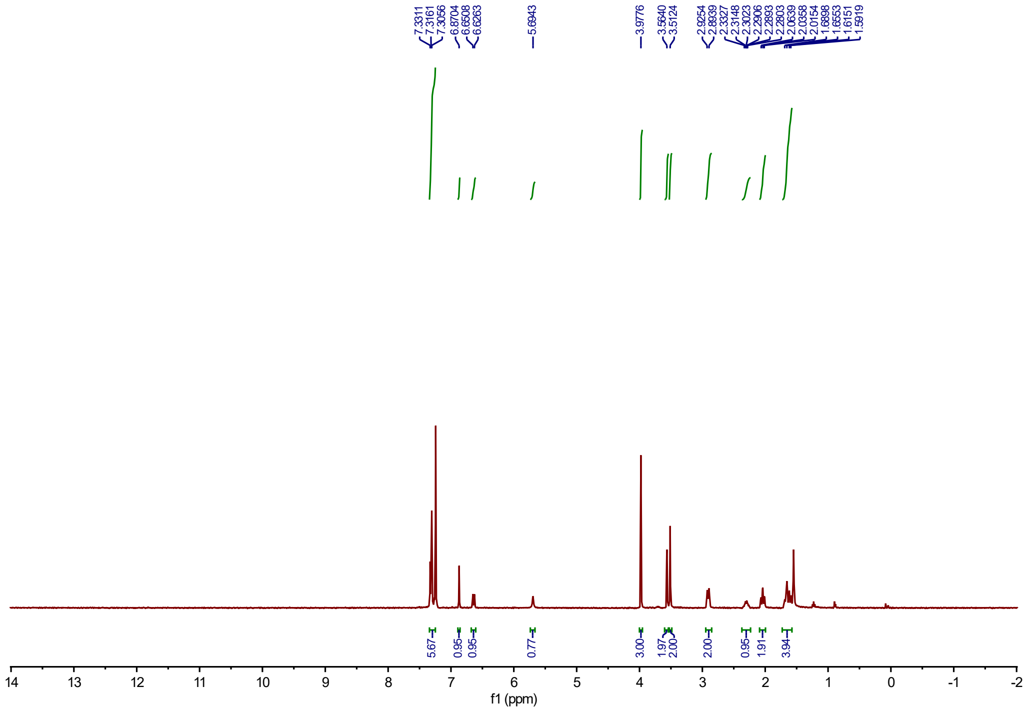

**Figure S31.** $^{1}H$ NMR spectrum for compound **13** in $CDCl_3$ (400 MHz).

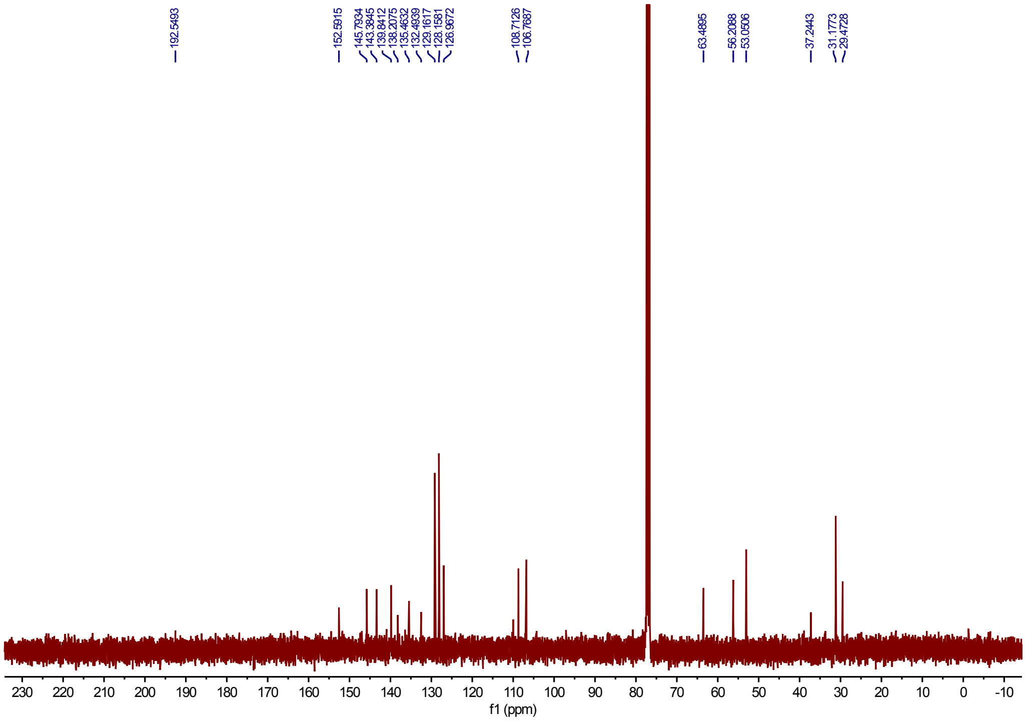


**Figure S32.** $^{13}$C NMR spectrum for compound **13** in $CDCl_3$ (100 MHz).

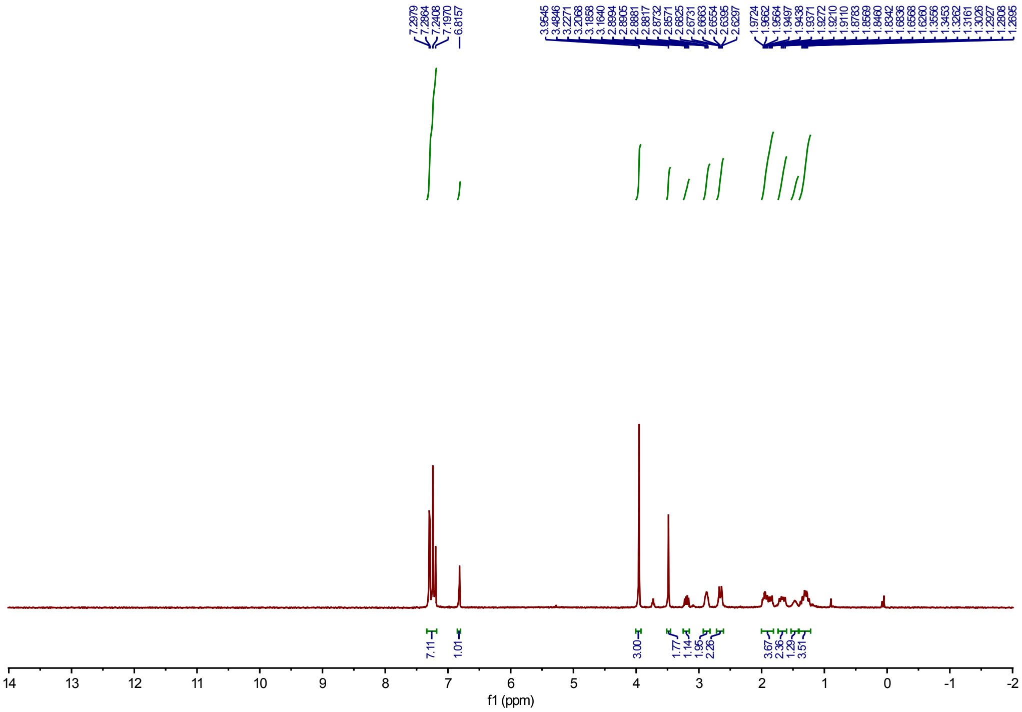


**Figure S33.** $^{1}$H NMR spectrum for compound **14** in $CDCl_3$ (400 MHz).

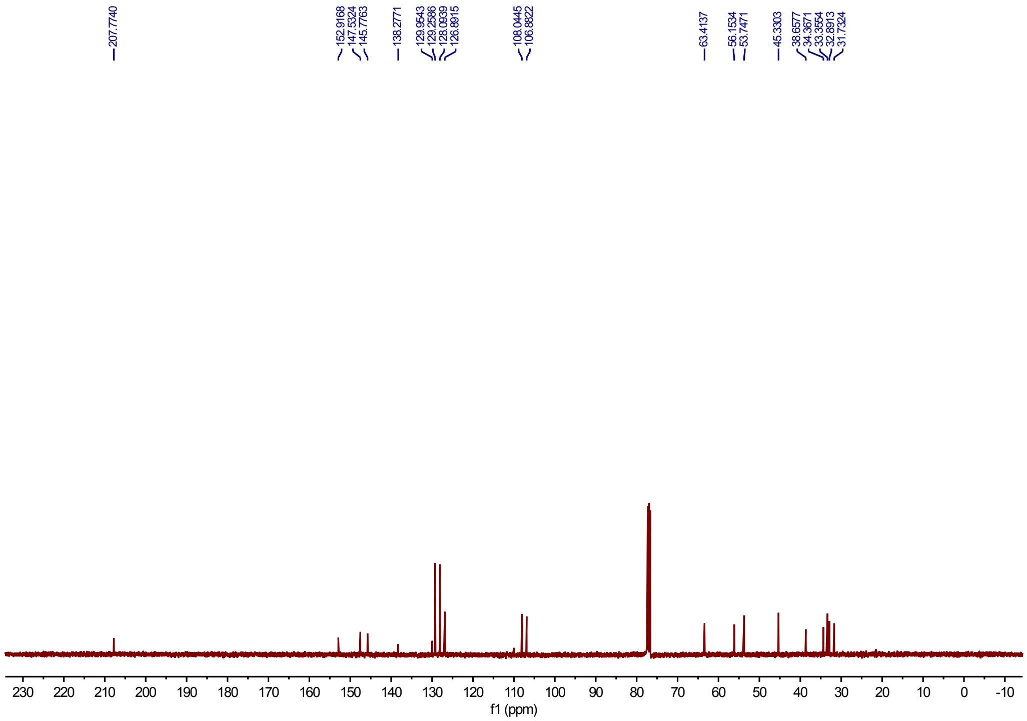

**Figure S34.** $^{13}C$ NMR spectrum for compound **14** in $CDCl_3$ (100 MHz).

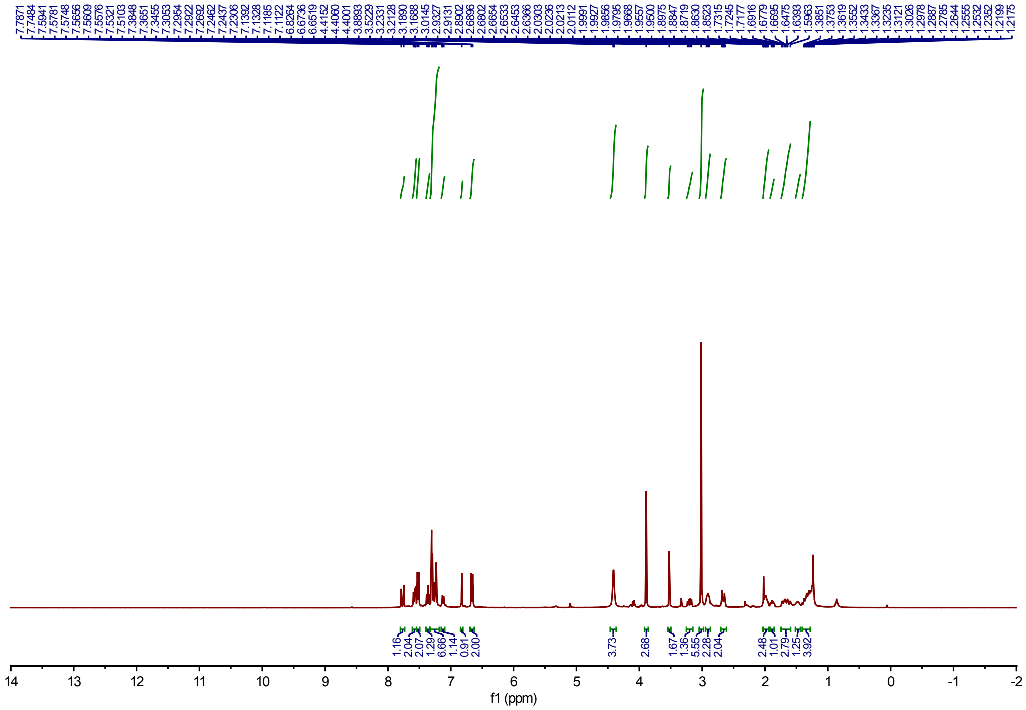

**Figure S35.** $^{1}H$ NMR spectrum for compound **15a** in $CDCl_3$ (400 MHz).

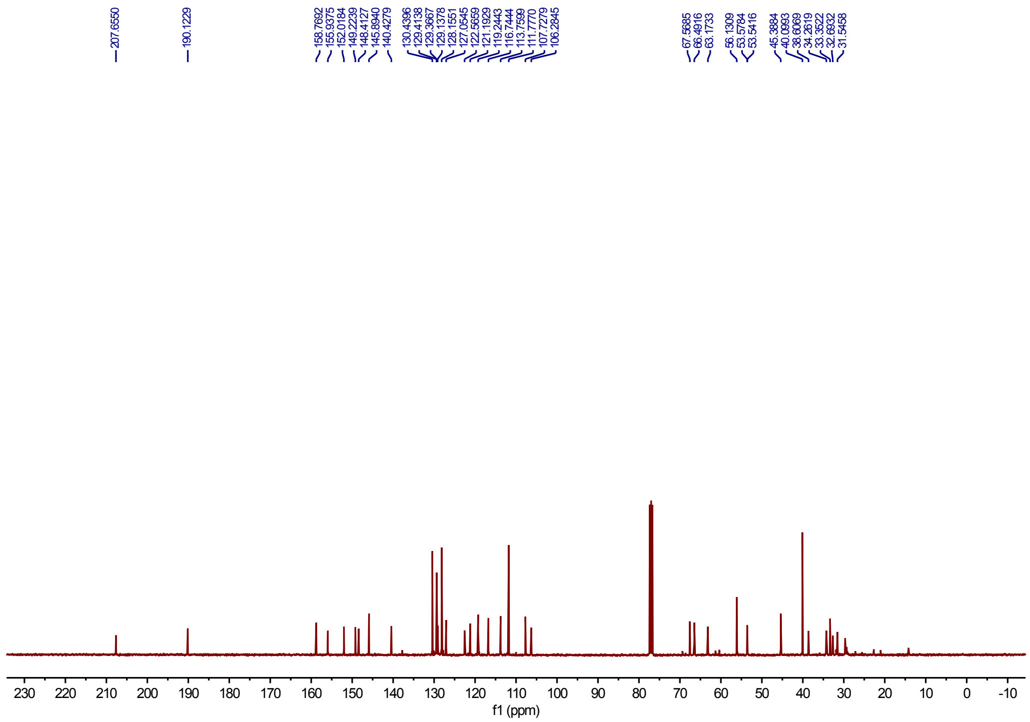

**Figure S36.** $^{13}C$ NMR spectrum for compound **15a** in $CDCl_3$ (100 MHz).

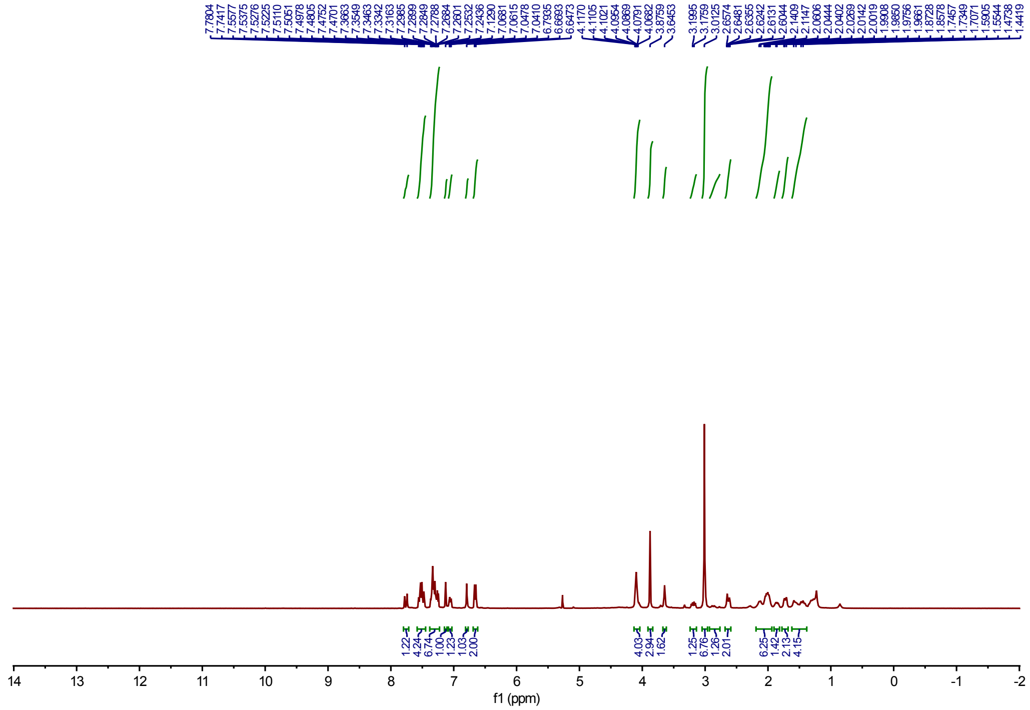

**Figure S37.** $^{1}H$ NMR spectrum for compound **15b** in $CDCl_3$ (400 MHz).

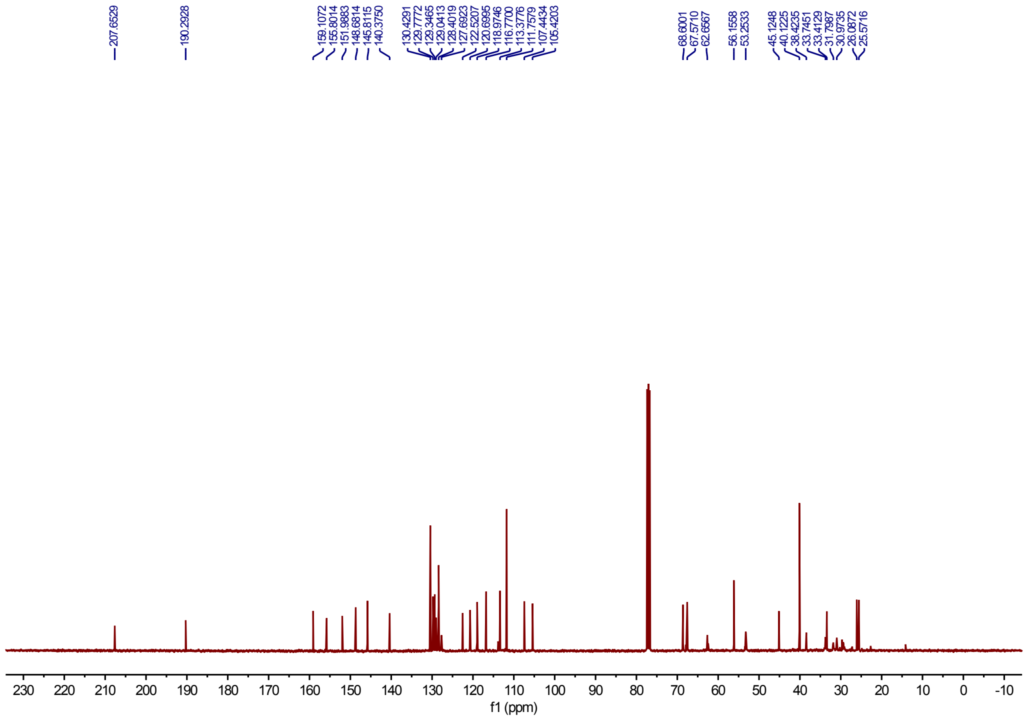

**Figure S38.** $^{13}$C NMR spectrum for compound **15b** in $CDCl_3$ (100 MHz).

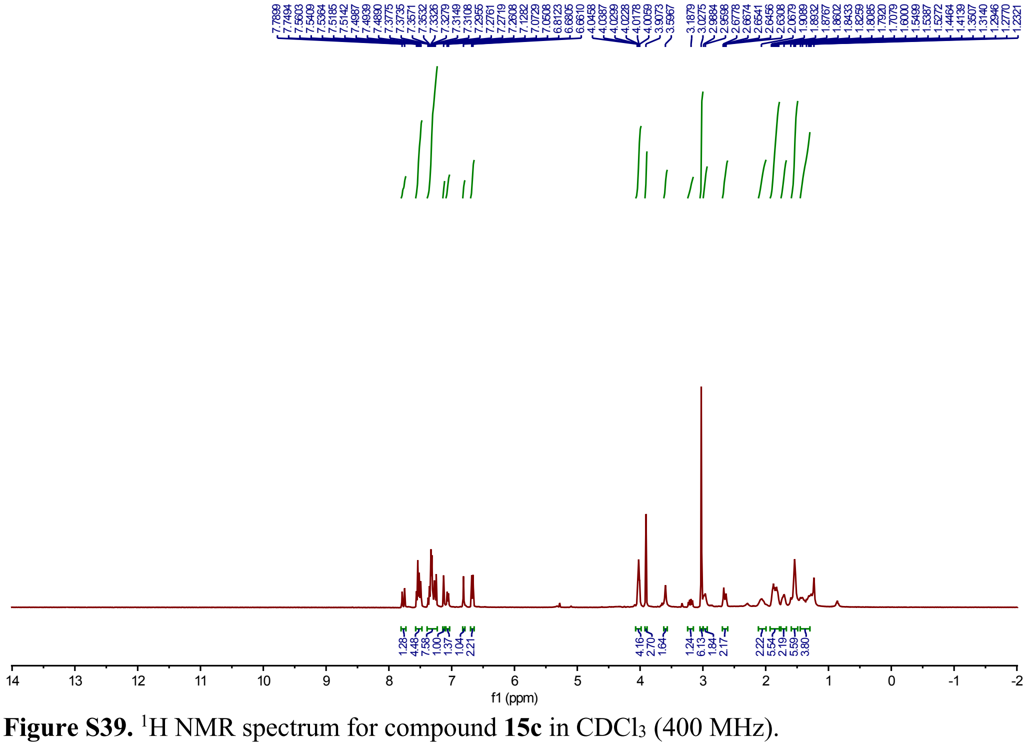

**Figure S39.** $^{1}$H NMR spectrum for compound **15c** in $CDCl_3$ (400 MHz).

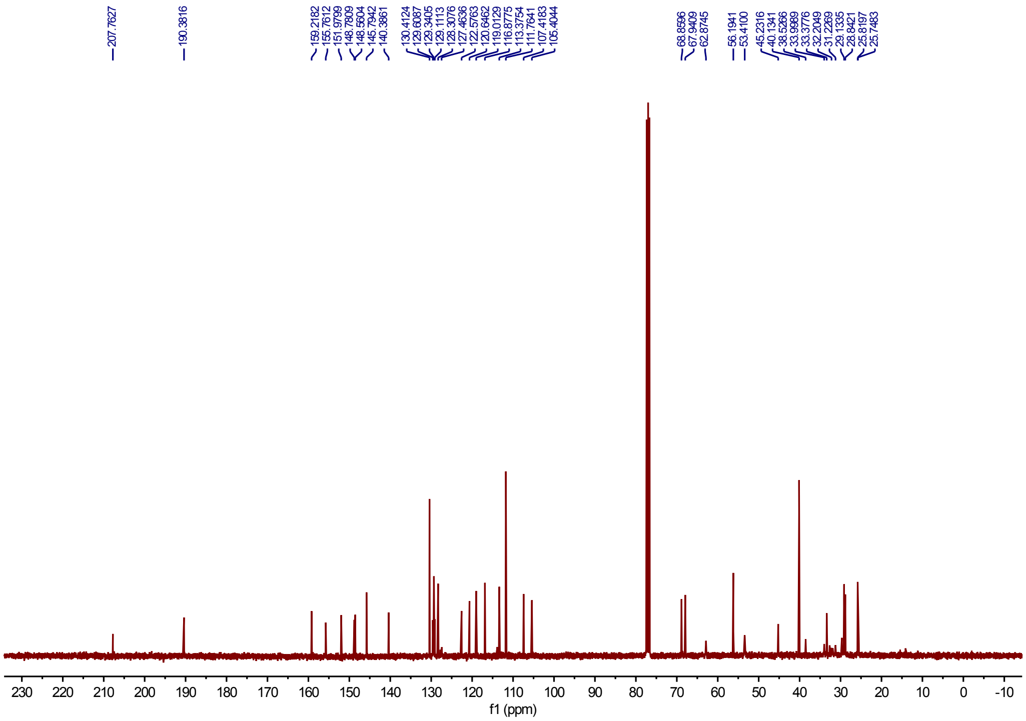

**Figure S40.** $^{13}$C NMR spectrum for compound **15c** in $CDCl_3$ (100 MHz).

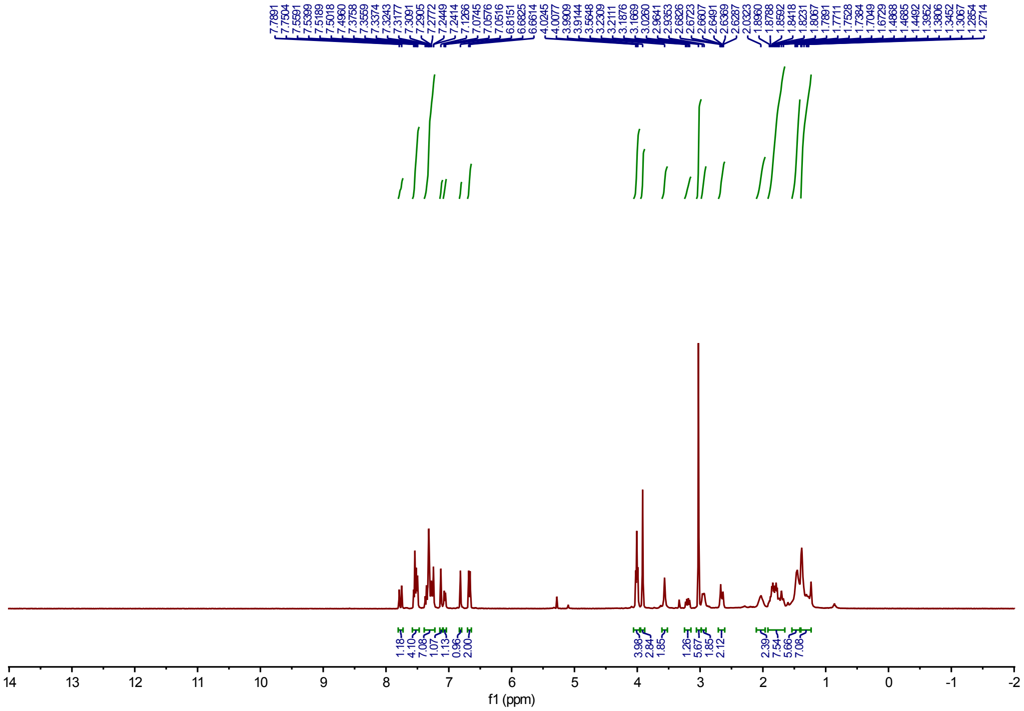

**Figure S41.** $^{1}$H NMR spectrum for compound **15d** in $CDCl_3$ (400 MHz).

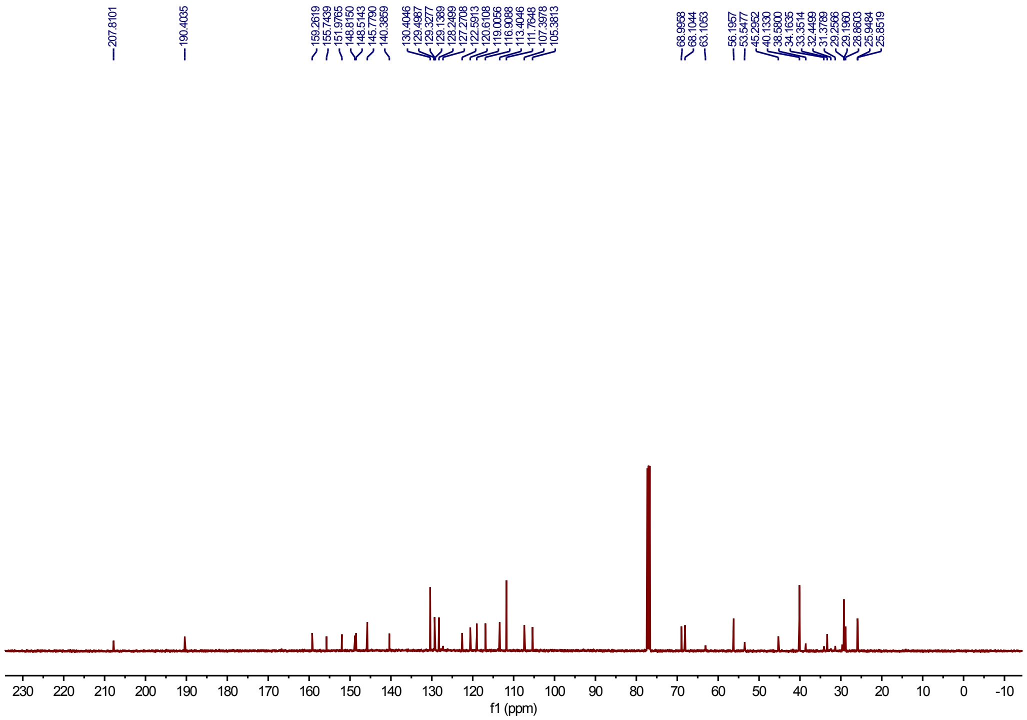

**Figure S42.** $^{13}$C NMR spectrum for compound **15d** in $CDCl_3$ (100 MHz).

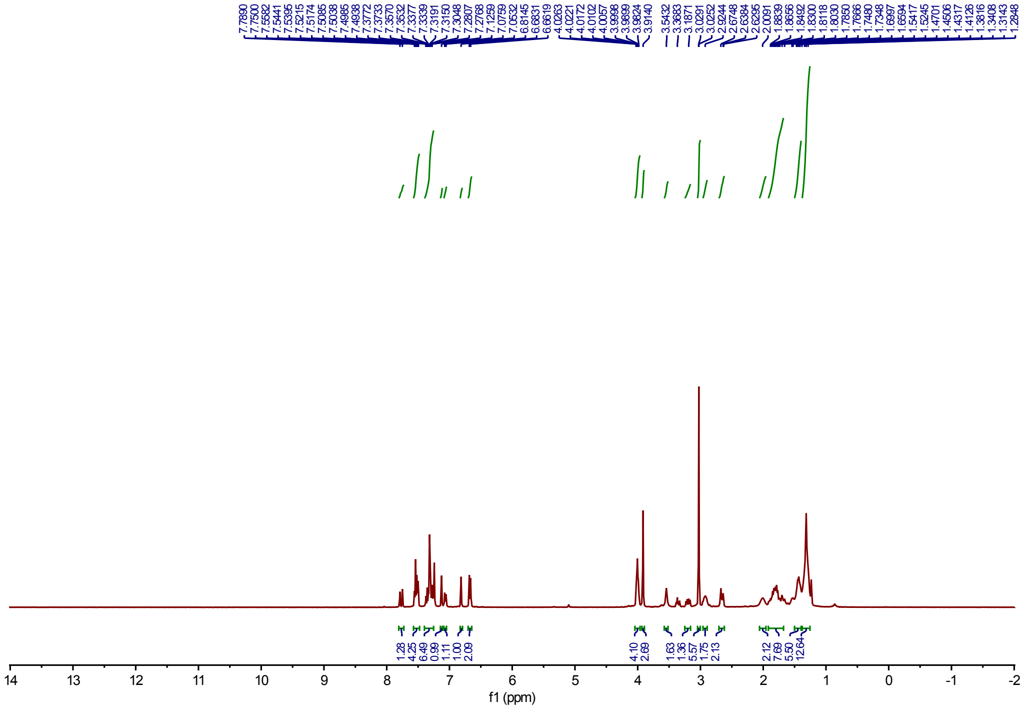

**Figure S43.** $^{1}$H NMR spectrum for compound **15e** in $CDCl_3$ (400 MHz).

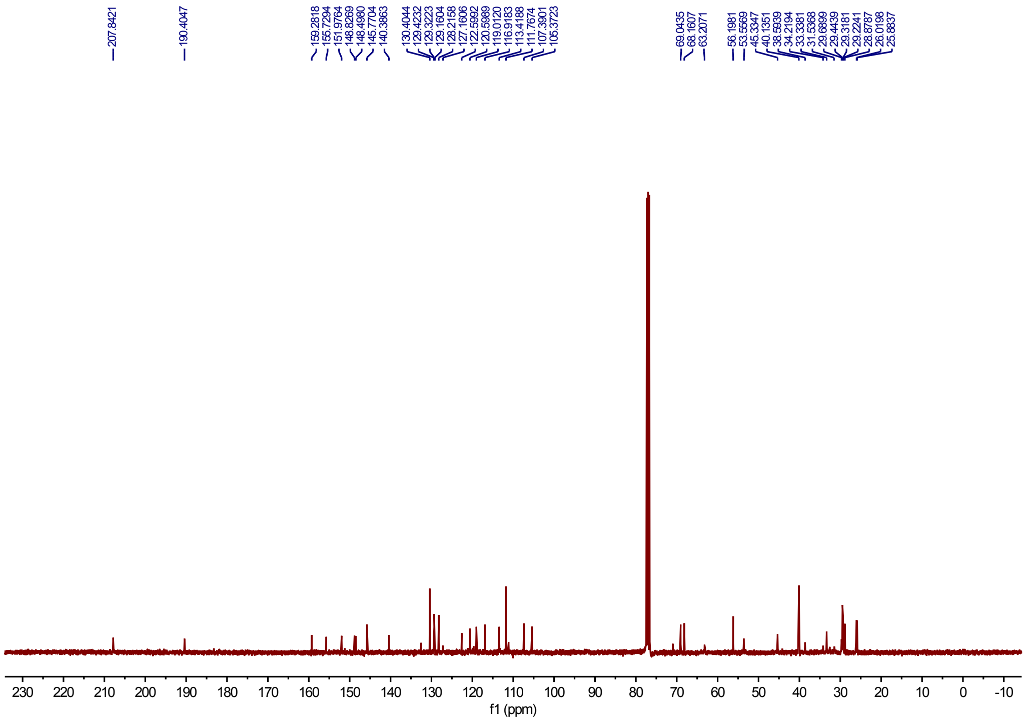

**Figure S44.** $^{13}$C NMR spectrum for compound **15e** in $CDCl_3$ (100 MHz).

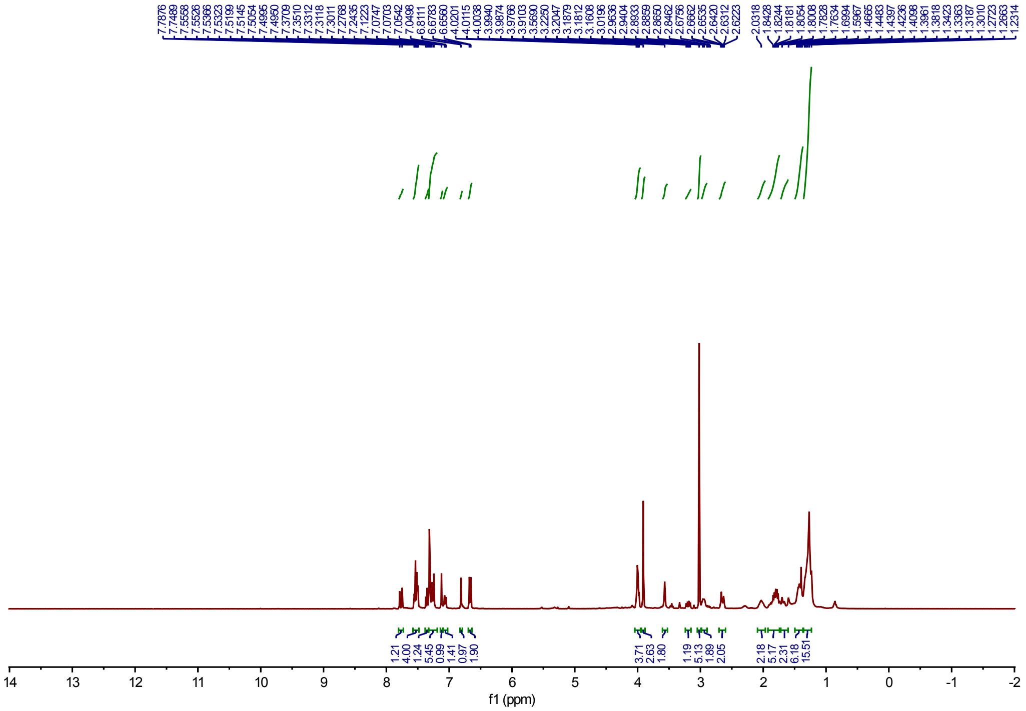

**Figure S45.** $^{1}$H NMR spectrum for compound **15f** in $CDCl_3$ (400 MHz).

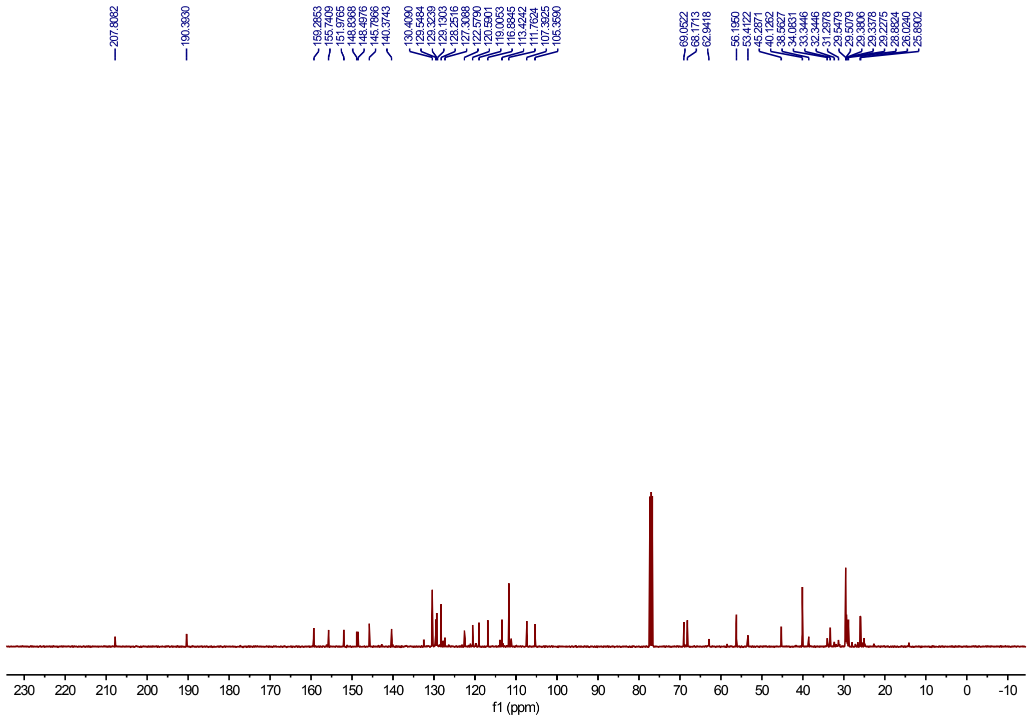

**Figure S46.** $^{13}$C NMR spectrum for compound **15f** in $CDCl_3$ (100 MHz).

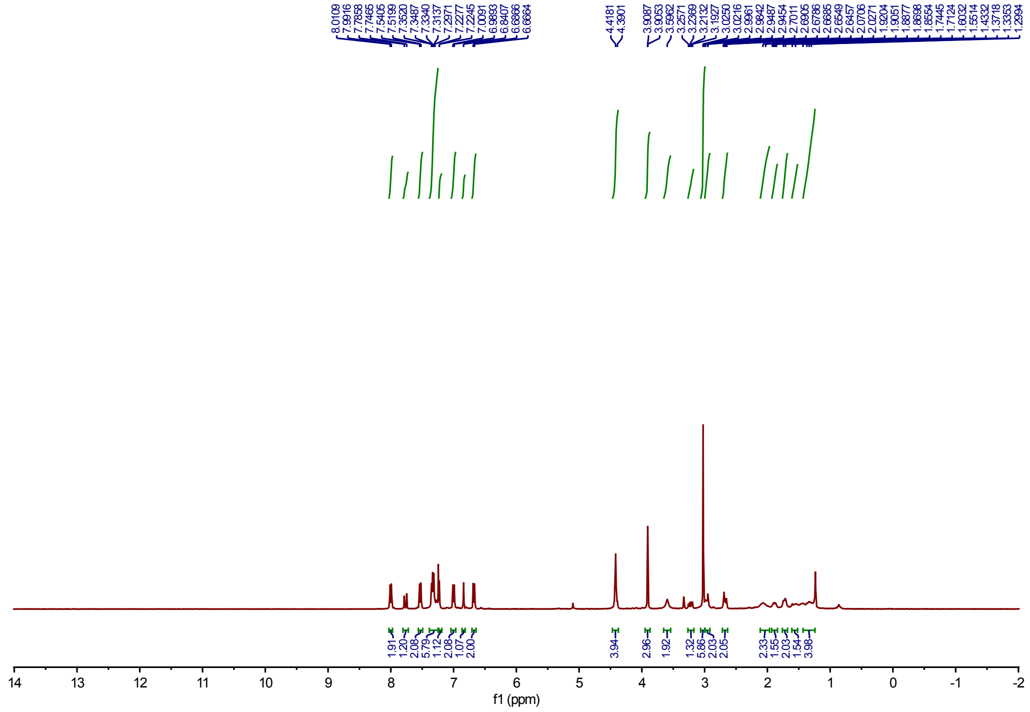

**Figure S47.** $^{1}$H NMR spectrum for compound **16a** in $CDCl_3$ (400 MHz).

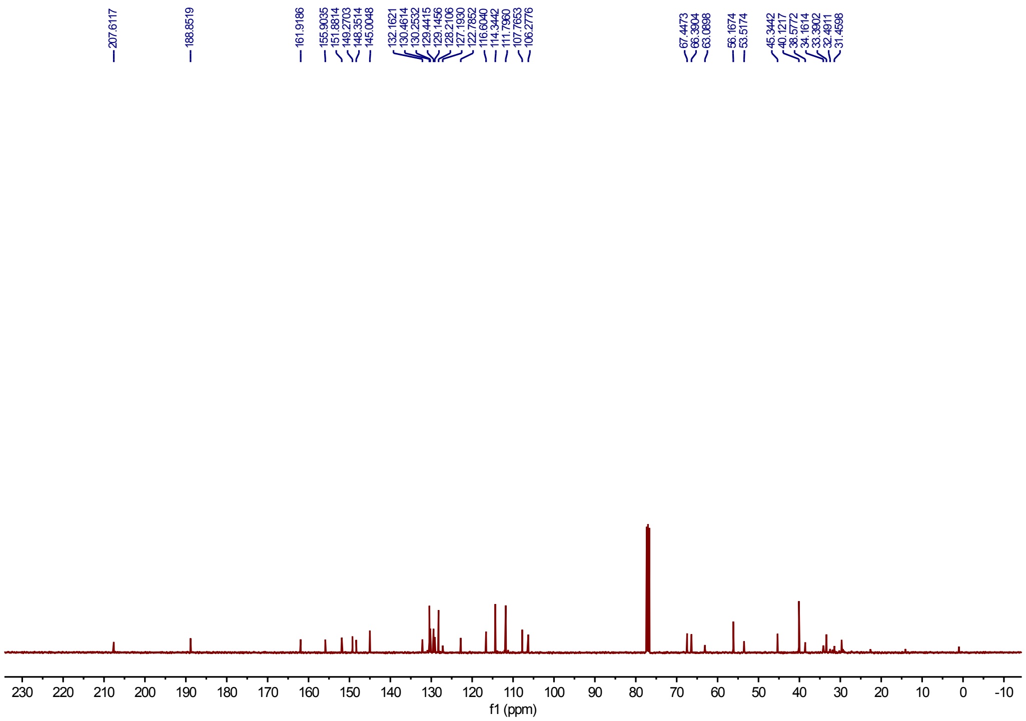


**Figure S48.** $^{13}$C NMR spectrum for compound **16a** in $CDCl_3$ (100 MHz).

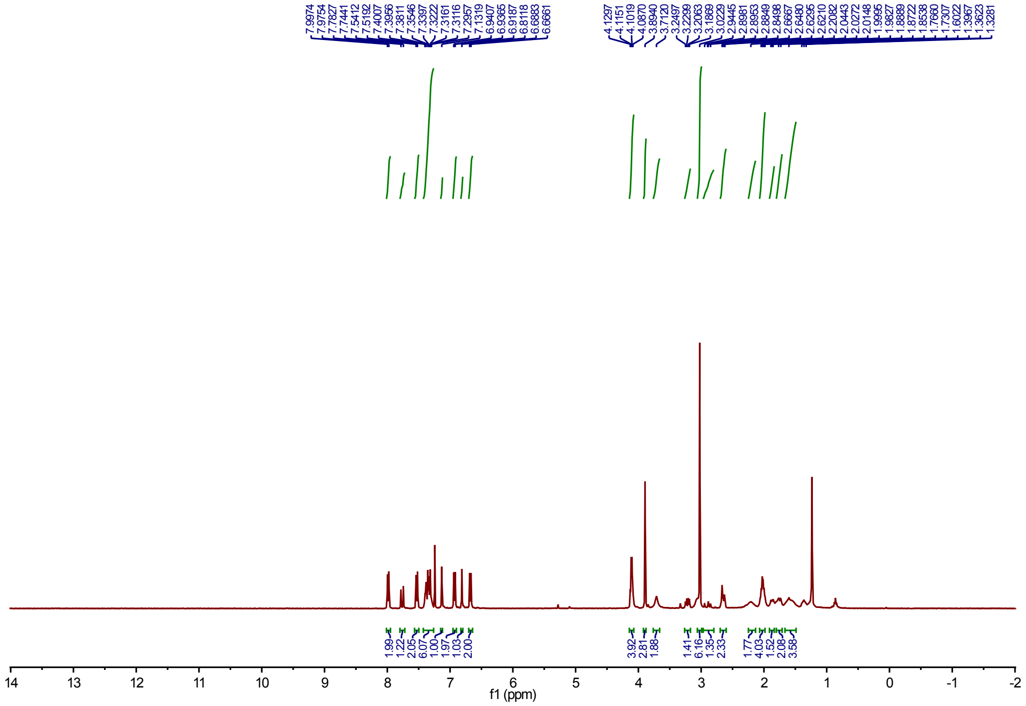


**Figure S49.** $^{1}$H NMR spectrum for compound **16b** in $CDCl_3$ (400 MHz).

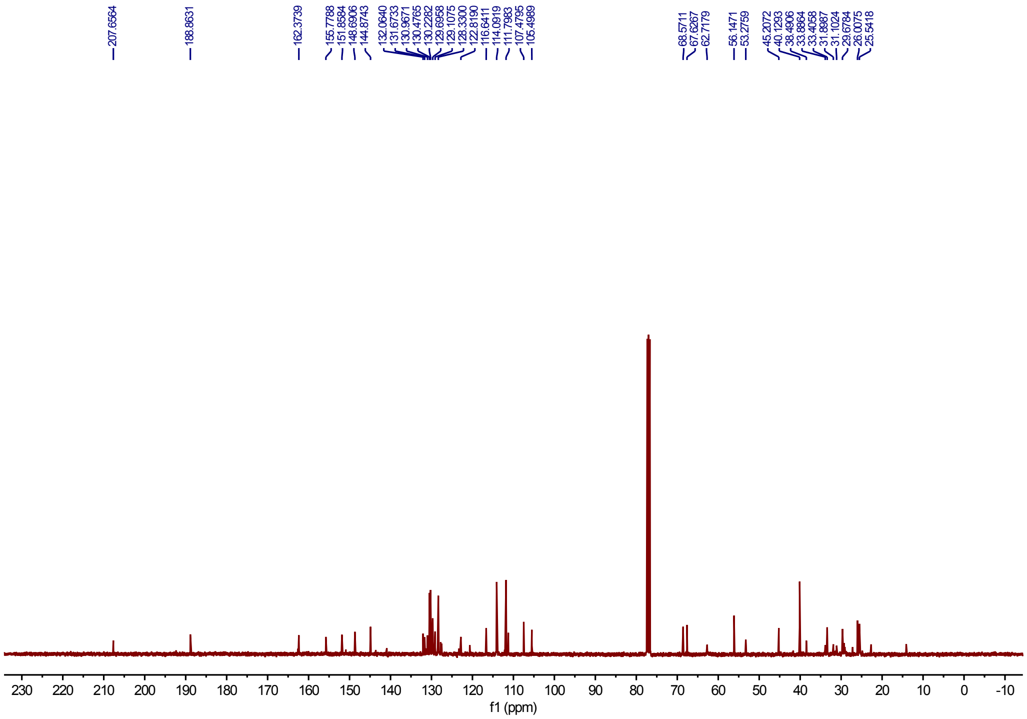


**Figure S50.** $^{13}C$ NMR spectrum for compound **16b** in $CDCl_3$ (100 MHz).

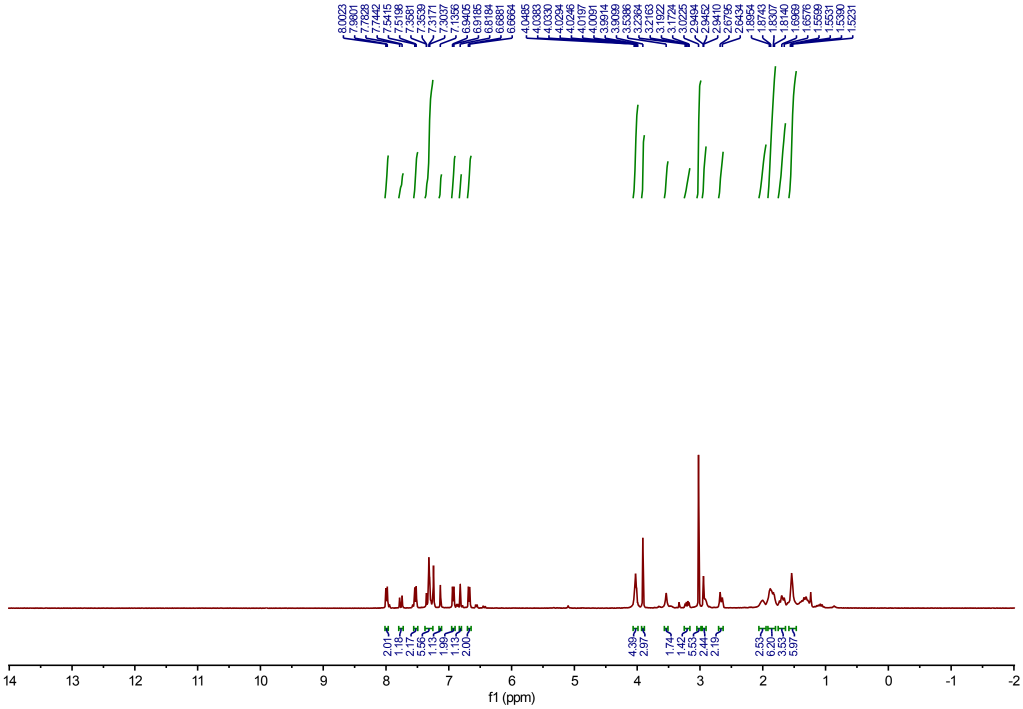


**Figure S51.** $^{1}H$ NMR spectrum for compound **16c** in $CDCl_3$ (400 MHz).

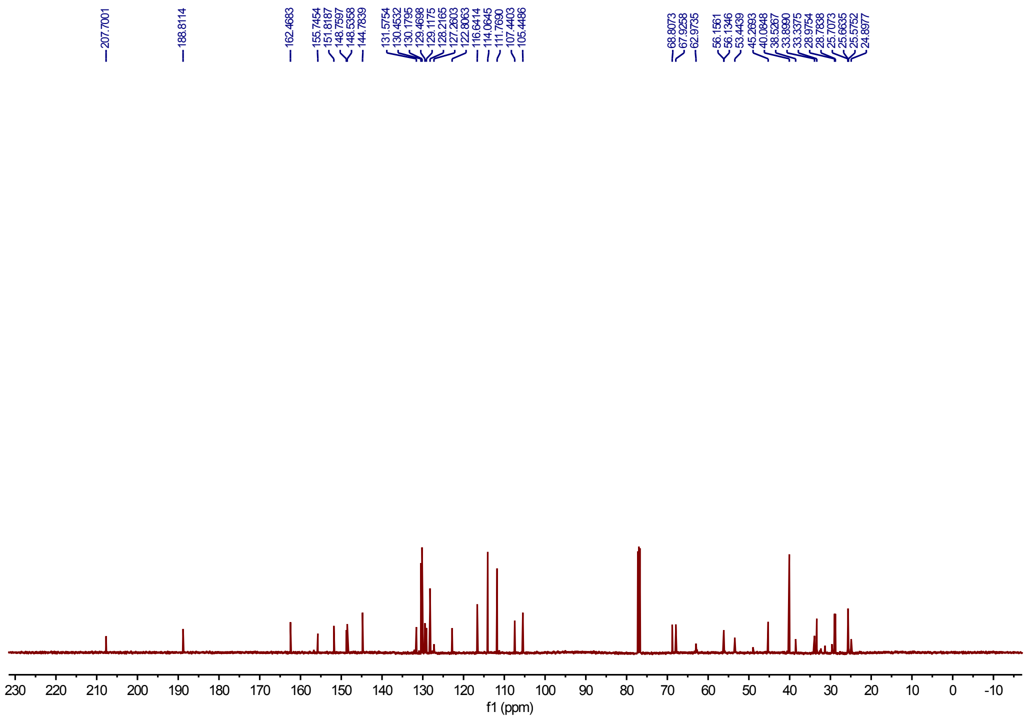

**Figure S52.** $^{13}$C NMR spectrum for compound **16c** in $CDCl_3$ (100 MHz).

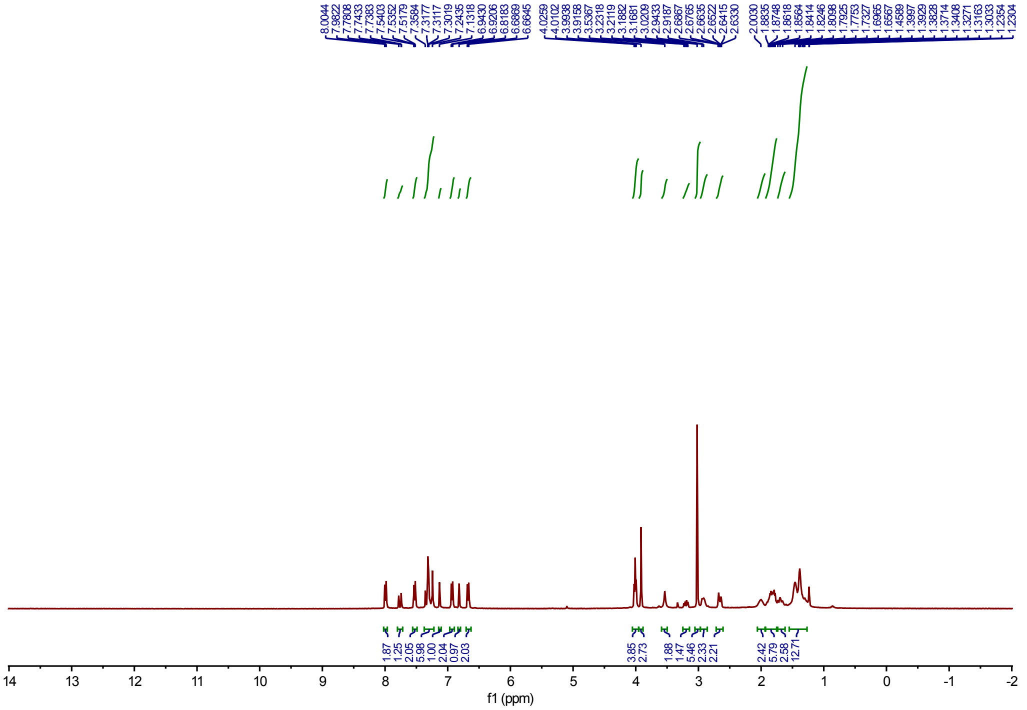

**Figure S53.** $^{1}$H NMR spectrum for compound **16d** in $CDCl_3$ (400 MHz).

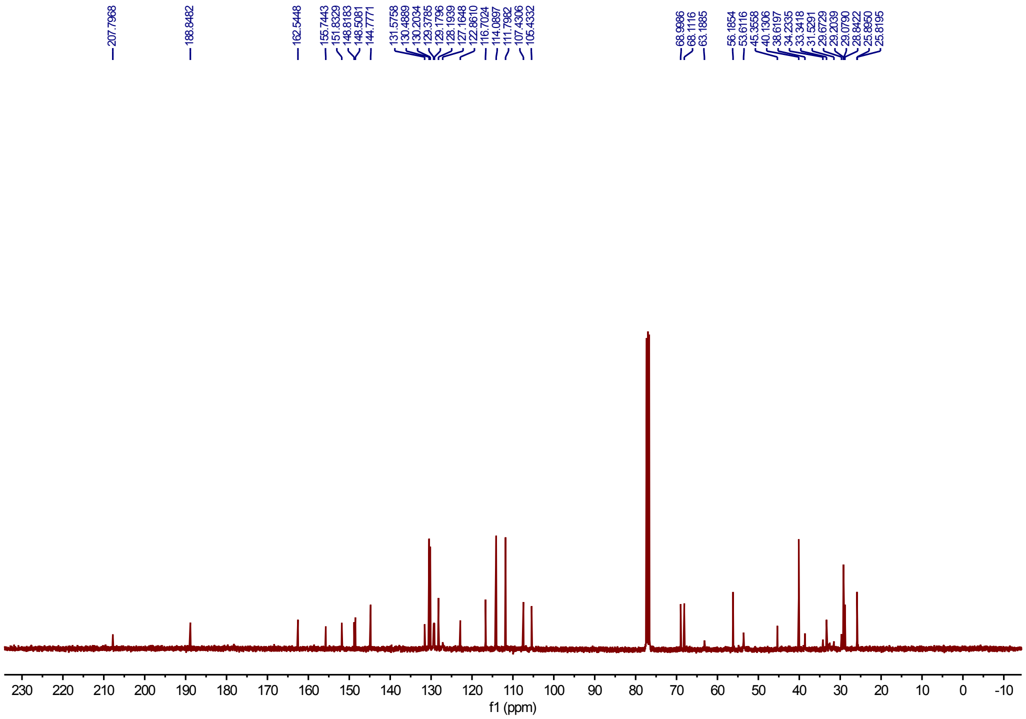


**Figure S54.** $^{13}$C NMR spectrum for compound **16d** in $CDCl_3$ (100 MHz).

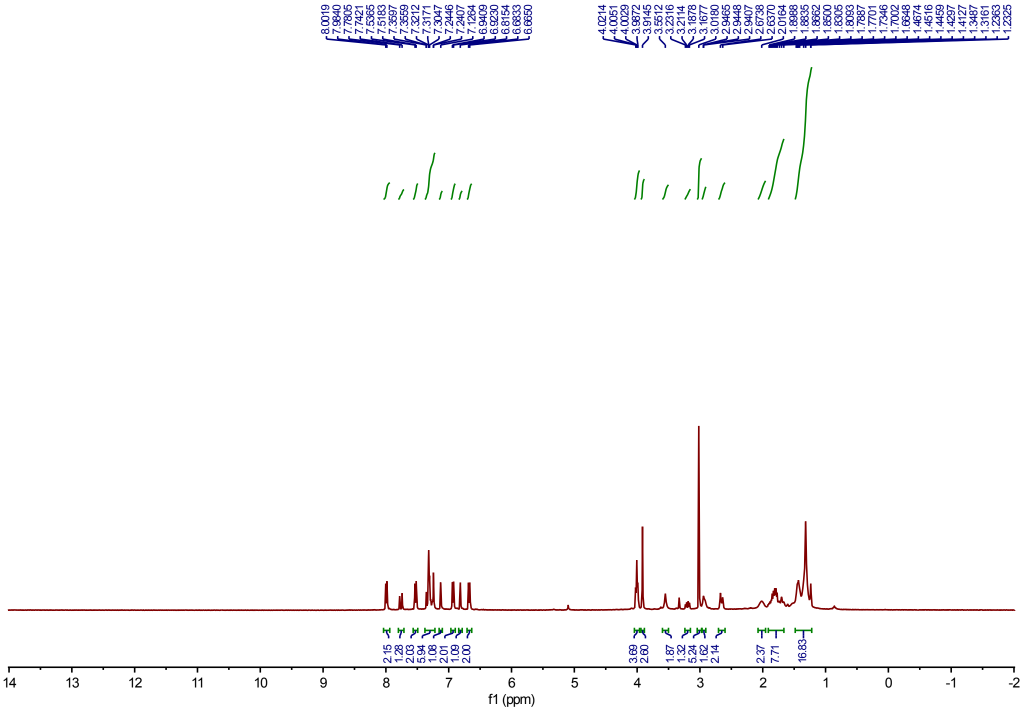


**Figure S55.** $^{1}$H NMR spectrum for compound **16e** in $CDCl_3$ (400 MHz).

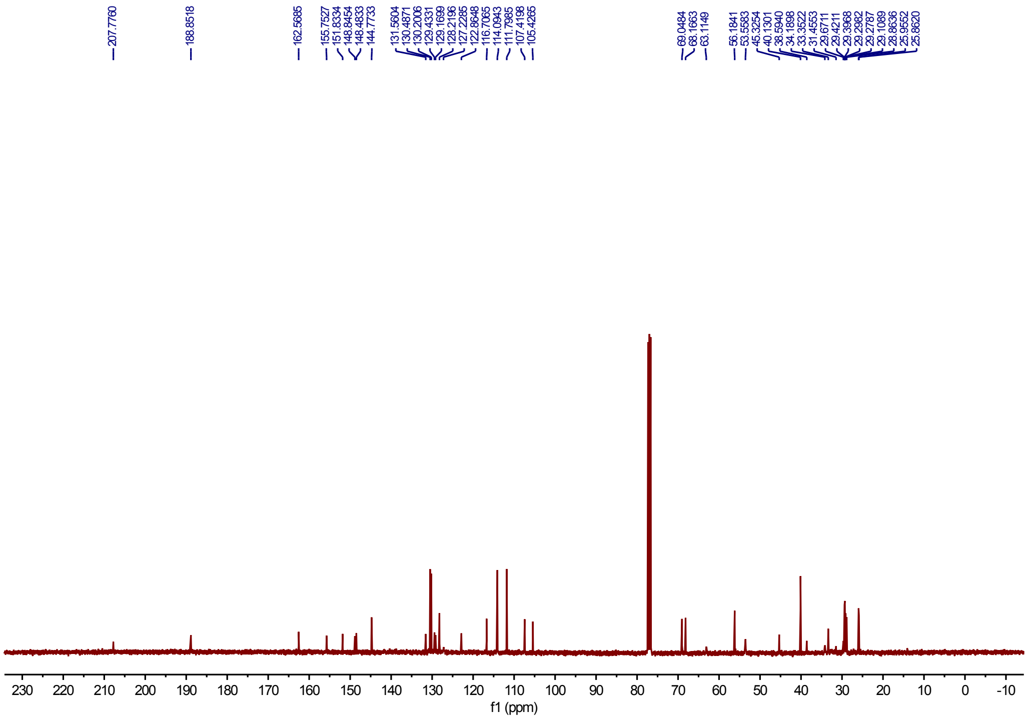


**Figure S56.** $^{13}$C NMR spectrum for compound **16e** in $CDCl_3$ (100 MHz).

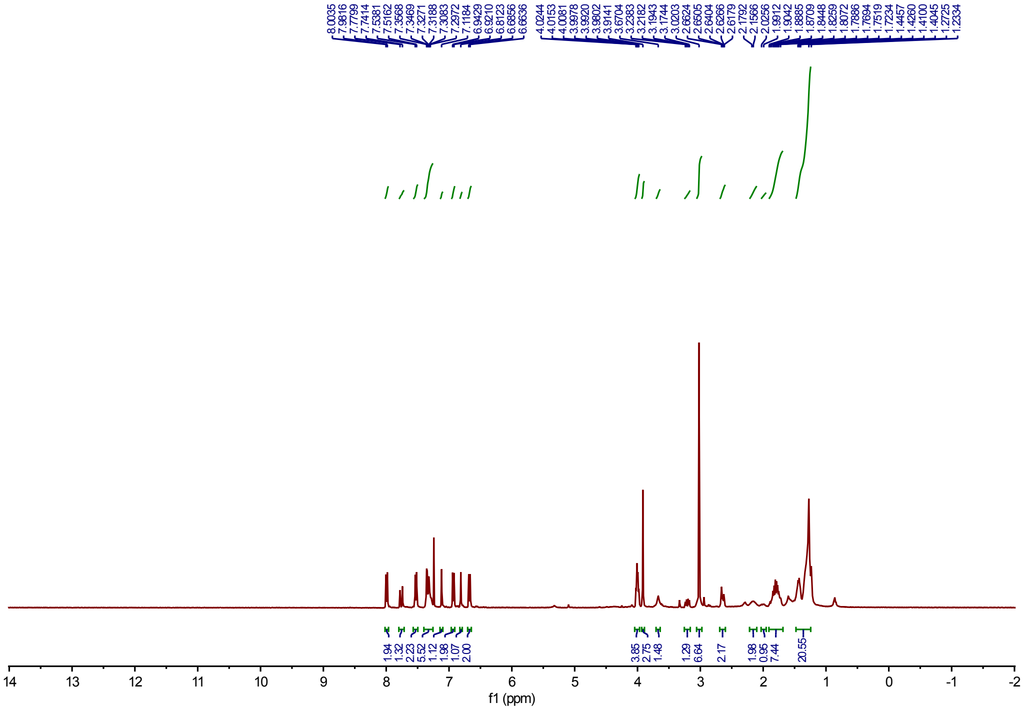


**Figure S57.** $^{1}$H NMR spectrum for compound **16f** in $CDCl_3$ (400 MHz).

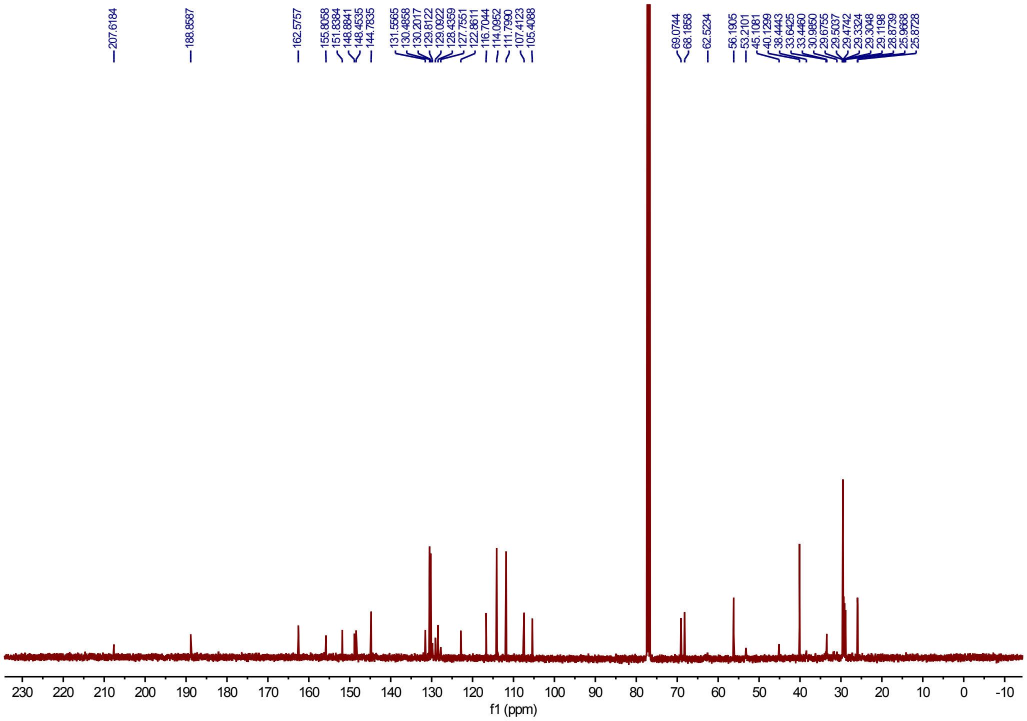

**Figure S58.** $^{13}C$ NMR spectrum for compound **16f** in $CDCl_3$ (100 MHz).

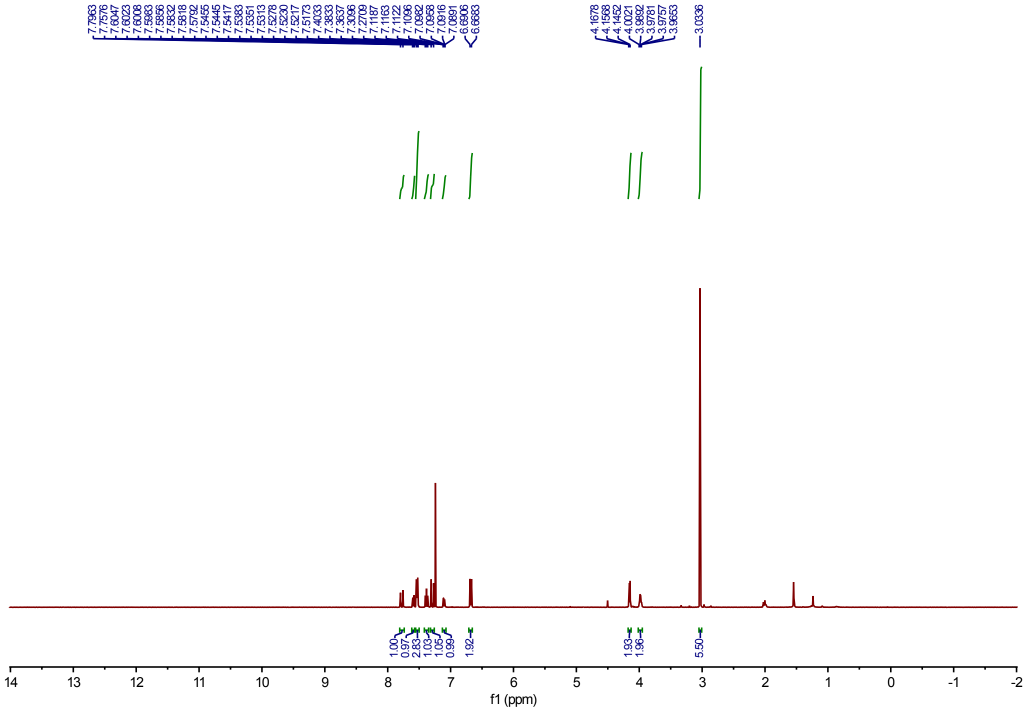

**Figure S59.** $^{1}H$ NMR spectrum for compound **17** in $CDCl_3$ (400 MHz).

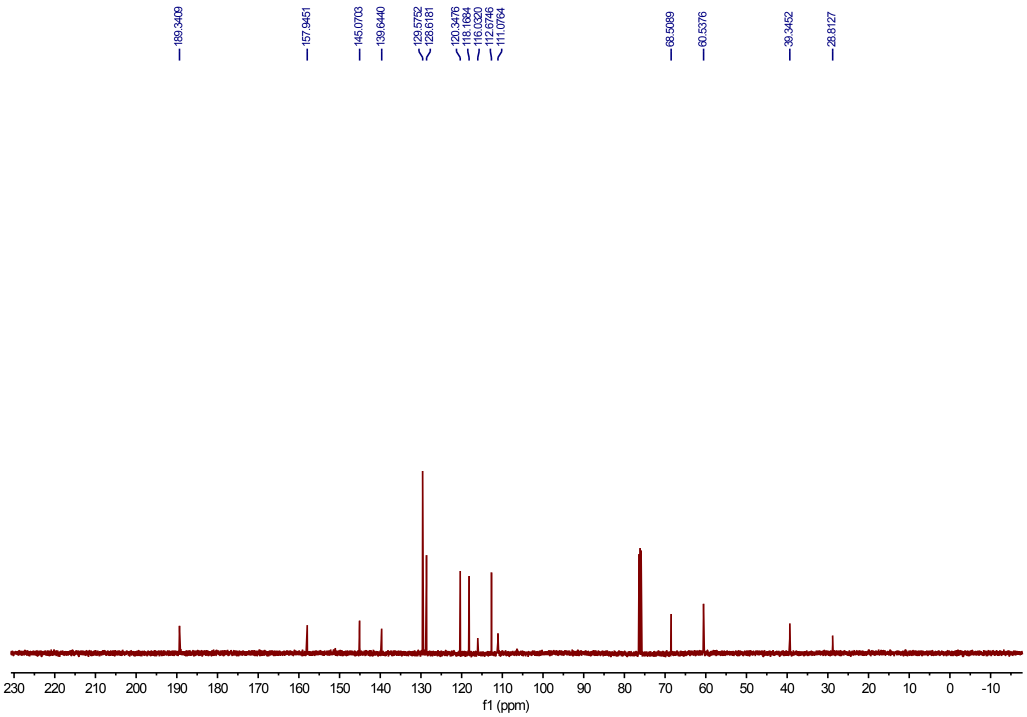

**Figure S60.** $^{13}$C NMR spectrum for compound **17** in $CDCl_3$ (125 MHz).

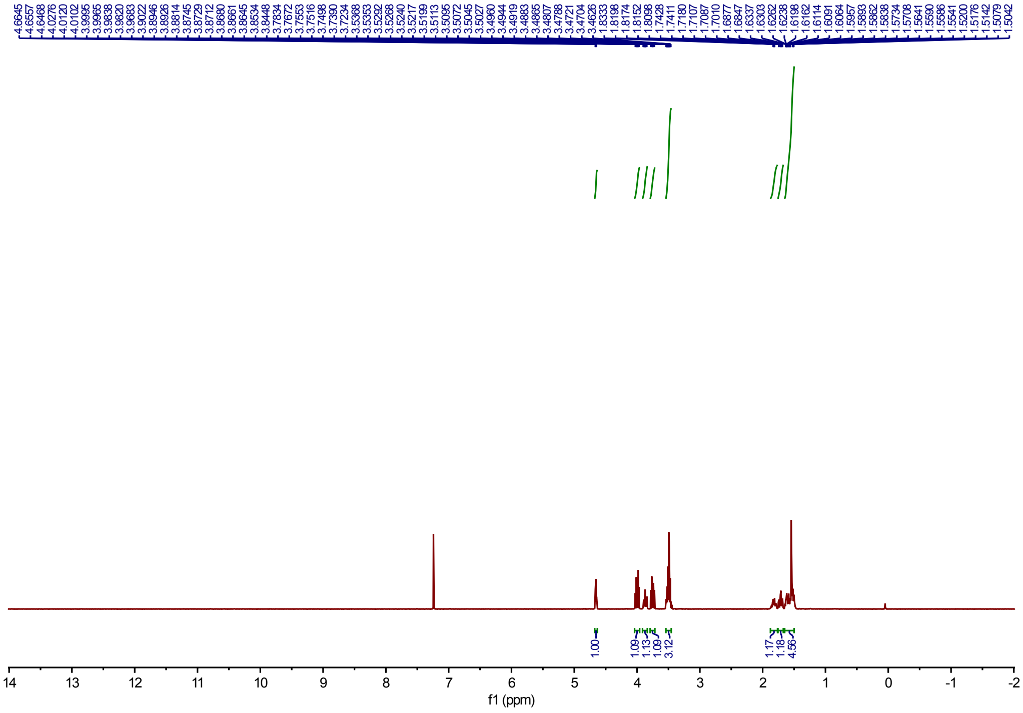

**Figure S61.** $^{1}$H NMR spectrum for compound **18** in $CDCl_3$ (400 MHz).

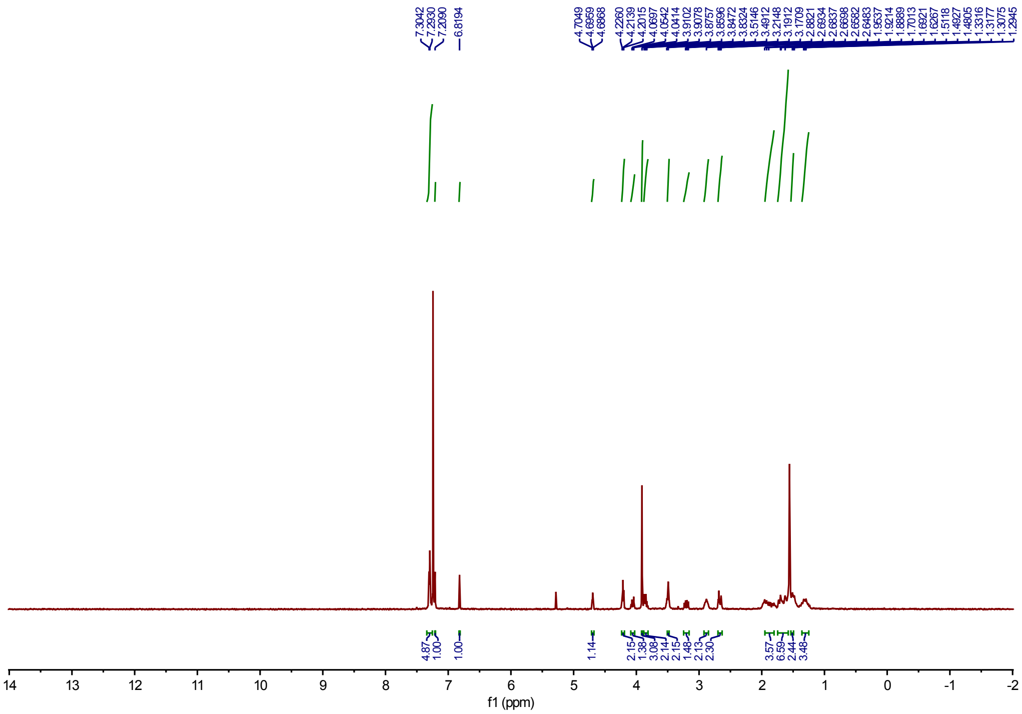

**Figure S62.** $^{1}H$ NMR spectrum for compound **19** in $CDCl_3$ (400 MHz).

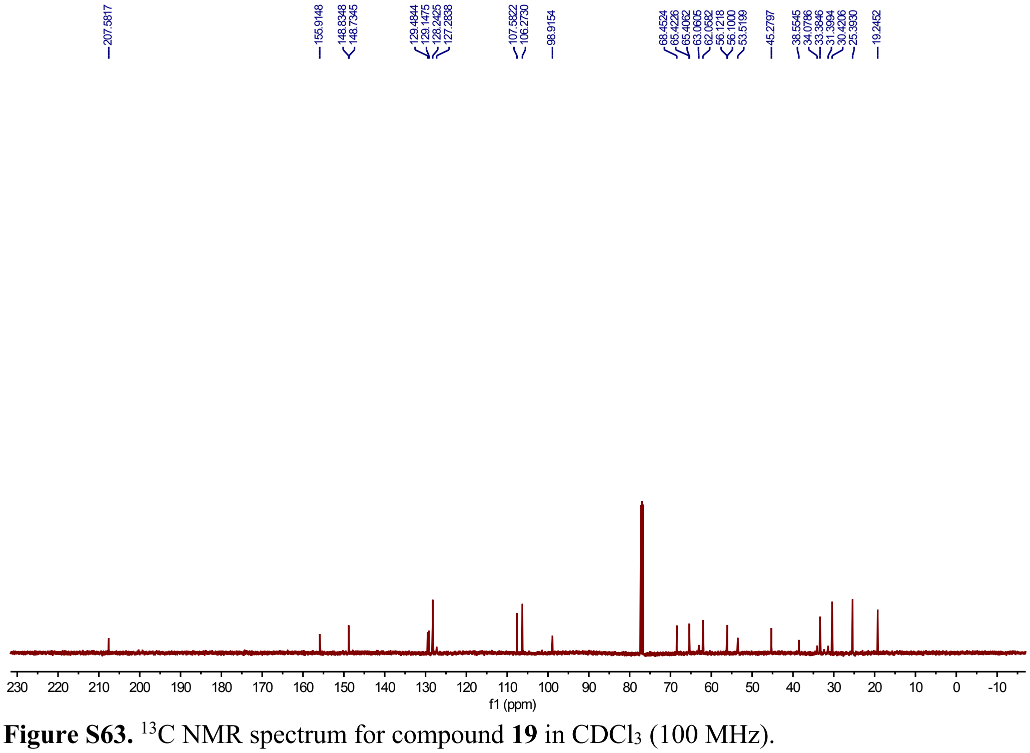

**Figure S63.** $^{13}C$ NMR spectrum for compound **19** in $CDCl_3$ (100 MHz).

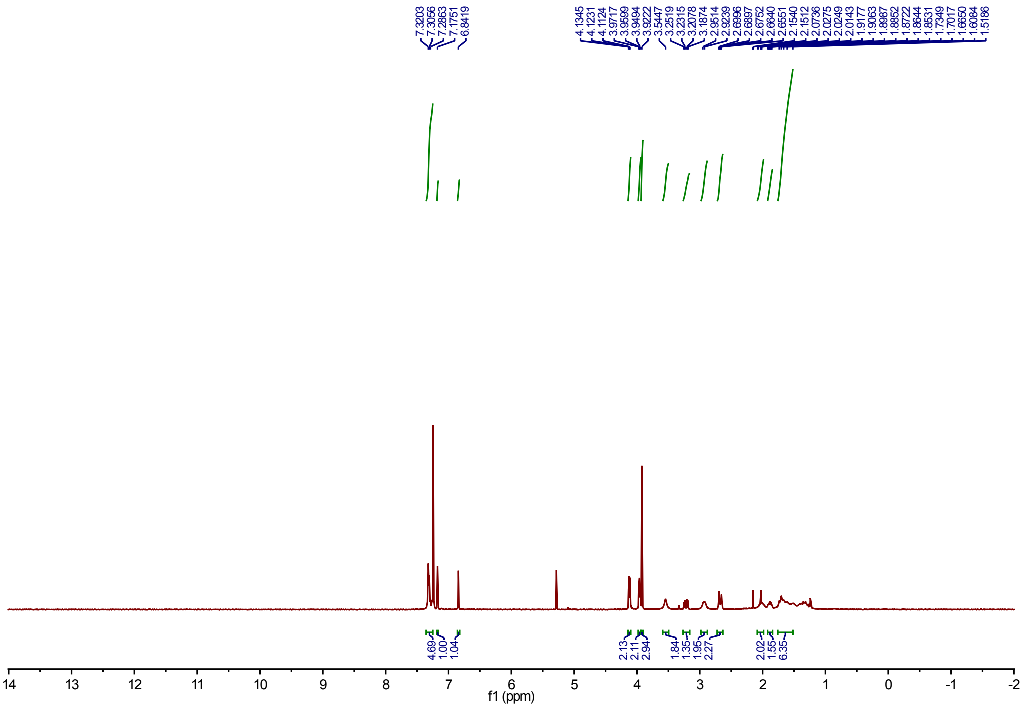

**Figure S64.** $^{1}H$ NMR spectrum for compound **20** in $CDCl_3$ (400 MHz).

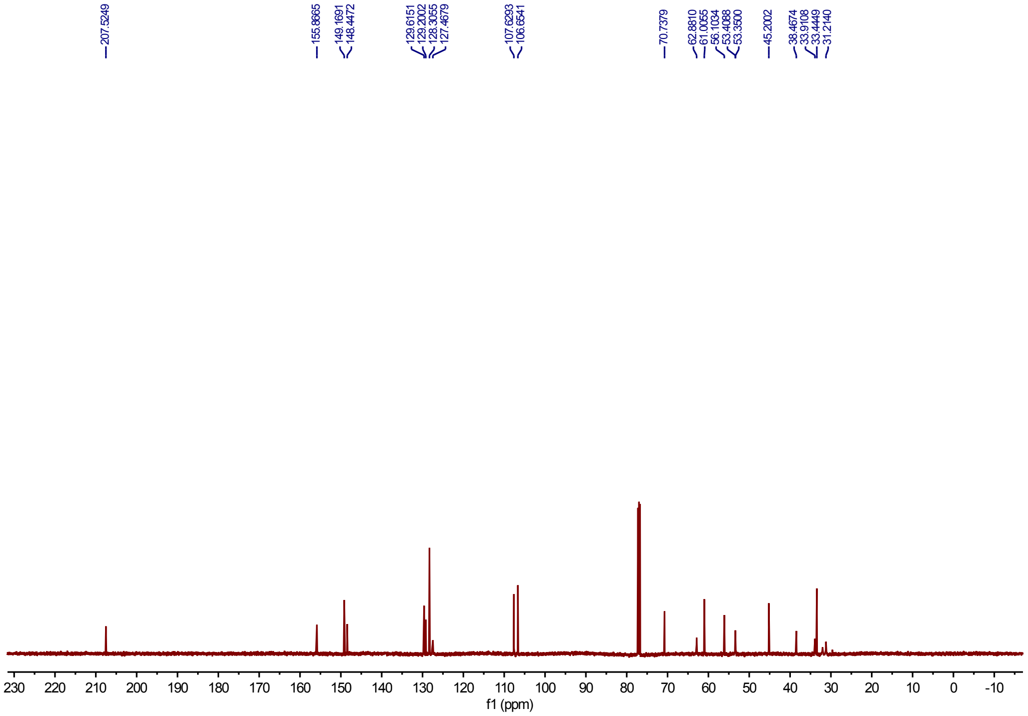

**Figure S65.** $^{13}C$ NMR spectrum for compound **20** in $CDCl_3$ (100 MHz).

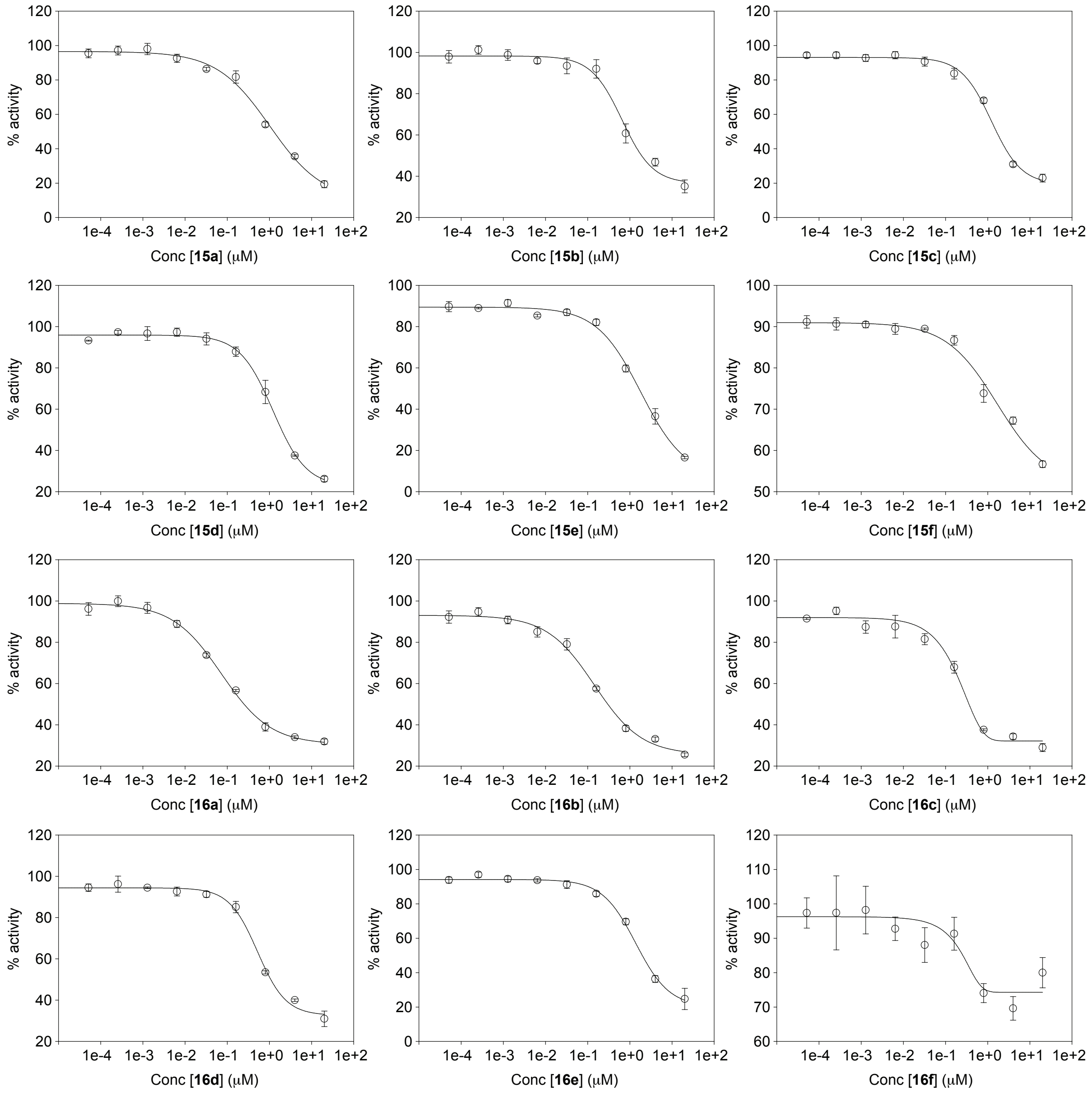


**Figure S66.** $IC_{50}$ curves for the inhibition of *Ee*AChE by chalcone-donepezil hybrids **15a**-**15f** and **16a**-**16f**.

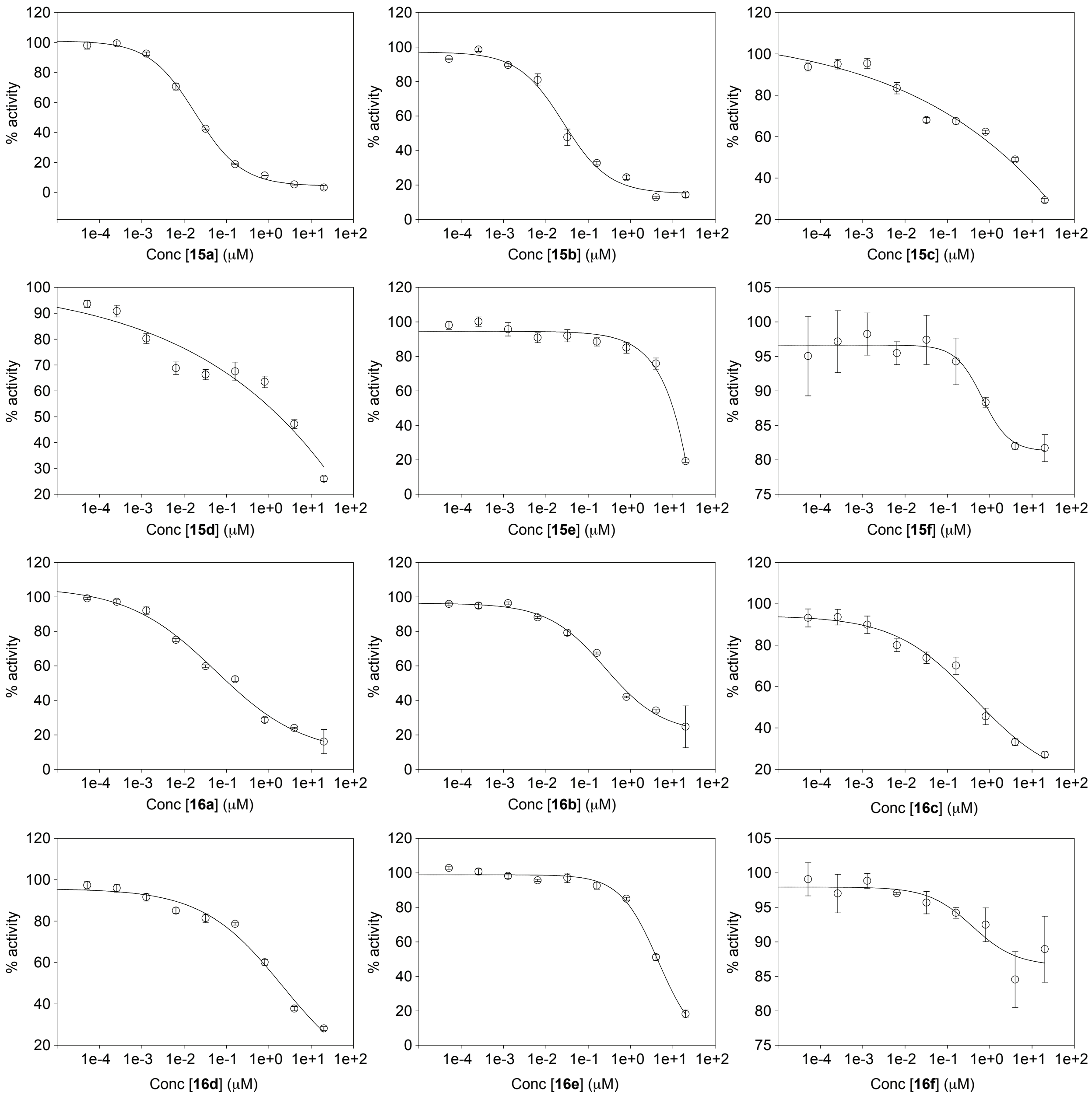


**Figure S67.** $IC_{50}$ curves for the inhibition of *Ef*BChE by chalcone-donepezil hybrids **15a-15f** and **16a-16f**.

| **Table S1.** $EC_{50}$ (μM) values of chalcones **3** and **6** as well as 1,3- and 1,4-chalcone-donepzil hybrids **15a-15f** and **16a-16f** against biotinyl-A$\beta_{(1-42)}$ (bioA$\beta_{42}$) oligomer assembly inhibition. | |
|---|---|
| **Cpd** | **$EC_{50}$**[a] |
| **3** | >50 |
| **6** | >50 |
| **15a** | 4.83 ± 0.47 |
| **15b** | 4.10 ± 0.17 |
| **15c** | 8.67 ± 1.15 |
| **15d** | 9.67 ± 1.15 |
| **15e** | 31.67 ± 15.95 |
| **15f** | 45.33 ± 4.51 |
| **16a** | 9.37 ± 2.10 |
| **16b** | 2.00 ± 0.20 |
| **16c** | 2.60 ± 0.46 |
| **16d** | 4.13 ± 0.15 |
| **16e** | 9.73 ± 0.55 |
| **16f** | 24.13 ± 5.43 |

[a] These values are presented as a bar graph in Figure 4.
